# The supportive care needs of Iranian couples during postpartum hospitalization: A protocol of design, implementation and evaluation of intervention

**DOI:** 10.1371/journal.pone.0350038

**Published:** 2026-06-03

**Authors:** Zahra Rastad, Shirin Shahbazi Sighaldeh, Zahra Behboodi Moghadam, Shahla Faal Siahkal, Elham Ebrahimi

**Affiliations:** 1 Department of Midwifery and Reproductive Health, School of Nursing and Midwifery, Tehran University of Medical Sciences, Tehran, Iran; 2 Midwifery and Reproductive Health Department, School of Nursing & Midwifery, Tehran University of Medical Sciences, Tehran, Iran; 3 Nursing and Midwifery Care Research Center, Department of Midwifery and Reproductive Health, School of Nursing and Midwifery, Tehran University of Medical Sciences, Tehran, Iran; 4 Department of Midwifery and Reproductive Health, School of Nursing and Midwifery, Tehran University of Medical Sciences, Tehran, Iran; 5 Department of Midwifery, Mara.C., Islamic Azad University, Marand, Iran; 6 Nursing and Midwifery Care Research Center, Department of Midwifery and Reproductive Health, School of Nursing and Midwifery, Tehran University of Medical Sciences, Tehran, Iran; 7 Al-Subtain University of Medical Science, International Branch of Tehran University of Medical Sciences in Iraq, Karbala, Iraq; Trinity College Dublin: The University of Dublin Trinity College, IRELAND

## Abstract

**Background:**

The immediate postpartum period is marked by significant physiological and hormonal changes, which may present mothers with various social, emotional, and functional challenges. The goal of optimal postpartum hospice care is to sustain and enhance the health of both mothers and newborns while fostering a supportive environment for families and communities to address diverse health and social needs. Implementing comprehensive supportive care programs that offer full coverage of services for women can ensure that their needs are adequately met during this critical postpartum phase. Consequently, this study aims to investigate the supportive care requirements of couples during the postpartum period using a mixed-methods approach, along with the design and implementation of a needs-based intervention to improve health services during this essential time.

**Methods:**

This study employs a multistage mixed-methods approach, structured in a sequential exploratory design consisting of three distinct phases. Initially, an exploratory qualitative study will be conducted utilizing a conventional content analysis framework to investigate the supportive care needs of couples during the postpartum hospitalization period. The second phase will involve a nominal group meeting in which the concerns and supportive care requirements identified by couples will be discussed in the presence of reproductive health specialists, policymakers, and experts. This collaborative effort will facilitate the prioritization of these needs. Following this prioritization, a comprehensive review of interventions and programs addressing couples’ supportive care needs during the postpartum hospitalization period on a global scale will be undertaken. Finally, after another panel of experts, the best intervention in this regard will be designed and consisted of a quantitative clinical trial aimed at evaluating the effectiveness of the intervention on the supportive care needs of Iranian couples during the postpartum period.

**Discussion:**

The results of this study can lead to the design of a comprehensive supportive care program with comprehensive coverage of couples’ needs during the postpartum hospitalization period. The study aims to ensure that couples’ needs are met in this critical period. If this program is effective, it can be included in postpartum health care guidelines. **Clinical trial registration No.** IRCT20110621006854N8 (2024-11-16)

## Background

The postpartum period is characterized by significant physiological and hormonal changes and presents mothers with specific social, emotional and functional challenges [1,2]. postpartum concerns commonly include anxiety, loneliness, low self-efficacy in parenting, difficulty accepting the maternal role, negative body image [1–6]. The goal of optimal postpartum care is to maintain and improve maternal and neonatal health by creating supportive conditions for the family unit to address its health-related and social needs [7]. However, studies indicate that a notable proportion of women report the care recieved in postpartum wards as inadequte [8–10]. Key barriers to optimal perinatal and postnatal health services include: perceived insensitivity of caregivers, dismissal of women’s concerns, delays in care provision, short hospitalization periods, and deficient counseling and supportive care measures [11].

Furthermore, a systematic review of women’s attitudes revealed a perception that postpartum care is deprioritized by healthcare providers, whose focus often remains on the newborn [12]. Participants frequently cited environmental and systemic factors, including overcrowded and chaotic postpartum units alongside insufficient flexibility to accommodate individual needs. Additional reported barriers encompassed structural and psychosocial dimensions: understaffing, excessive visitation, policies restricting immediate postpartum contact (and, in some cases, requiring extended neonatal hospitalization). Critically, women highlighted a profound lack of supportive counseling to address challenges in adapting to transformed intimate and family relationships, resuming sexual activity, developing parenting competencies, processing childbirth trauma, and managing concerns about pelvic floor health and body image. [12–19].

Fathers are seldom provided the opportunity or facilities to remain in hospital at night with their wives and children [12,13]. Furthermore, the specific sources of support valued by fathers—which may differ from those sought by mothers—remain under-researched. Evidence indicates the paternal poor mental health can affect the child’s emotional and cognitive development and marital relationship [20].

Additional systemic barriers include the receipt of care that falls below expected professional standards and contradictory recommendations from different caregivers [21]. These perceptions are further shaped by broader sociocultural factors. Predominant among these are the societal framing of childbirth, the progressive medicalization of pregnancy and birth, a dominant technocratic (biomedical) model of care, and the limited participation of women in their own healthcare decisions [22]. Consequently, hospitalization for childbirth has become a cultural norm, reinforced by a perception of childbirth as a high-risk, potentially damaging, and uncontrollable event [23]. This shift is widely attributed to the medicalization of birth, which can undermine women’s self-efficacy in childbirth and newborn care, particularly in contexts with limited access to alternative information sources [24]. Within this paradigm, maternal anxiety regarding the health of both themselves and their newborn, outside the context of immediate medical supervision, is a comprehensible response [24].

Respectful care is a fundamental expectation of mothers; however, significant disparities exist between this expectation and actual clinical experiences [23]. Disrespectful care manifests as verbal or physical abuse, coercion, or the assertion of dominance and power by healthcare providers. Research indicates that vulnerable populations including adolescents, women of low socioeconomic status, and HIV-positive women, are at a disproportionately higher risk of experiencing such disrespectful care [21].

Notably, postpartum healthcare in Iran has historically emphasized the prevention and management of immediate biomedical consequences, with a relative neglect of psychological and social support for parents [9]. This model stands in contrast to the comprehensive, holistic approach recommended by the World Health Organization (WHO), which advocates for care that is respectful, individualized, family-centered, and culturally appropriate. Consequently, within the Iranian healthcare context, the systematic management of common postpartum discomforts, emotional disturbances, and challenges in parental role adaptation remains inadequately addressed [25,26].

The inflexible, standardized length of postpartum hospital stay, which often fails to align with individual women’s needs, represents a significant barrier to patient-centered care. Over a decade ago, the UK Audit Commission cautioned against predefined hospitalization periods, advocating instead for flexible policies developed in consultation with women regarding both the duration of their stay and the nature of care received [27,28]. The Commission emphasized that any decision to shorten the postpartum stay must be contingent upon a thorough assessment of the mother’s and newborn’s physical and mental health status, as well as the mother’s satisfaction with care [28]. Therefore, developing alternative, comprehensive postpartum care models requires a foundational assessment from women’s own perspectives. A robust evaluation must measure the impact of such programs not only on the clinical health of women and newborns but also on parental psychosocial outcomes—such as anxiety and self-confidence—and on the economic implications for health services and families [13].

Collectively, the literature reveals a substantial mismatch between the current provision of healthcare services and the comprehensive support required by mothers, highlighting a persistent gap between expectations and the reality of perinatal and postnatal care [26]. Notably, research specifically addressing the experiences and needs of fathers during this period remains critically scarce [20,29].

Within Iran, the existing body of research on hospital-based postpartum care is limited and predominantly quantitative, primarily measuring women’s satisfaction and consistently reporting low levels of perceived care adequacy [9,10,30]. Furthermore, studies that do assess needs often focus narrowly on educational requirements, neglecting the multifaceted physical, emotional, and social dimensions of care for both partners, particularly during hospitalization [31]. An illustrative qualitative study by Sharifipour et al. examined primiparous mothers’ perceptions and identified comprehensive support as a paramount need, emphasizing the significant role of healthcare providers in delivering essential services [32]. This finding underscores the central importance of professional support systems, yet the study was confined to mothers’ perspectives.

Therefore, while the necessity for comprehensive, family-centered postpartum care is evident, there is a pressing need for research that:

- Simultaneously captures the perspectives of both mothers and fathers.
- Employs a mixed-methods design to explore the depth of experiences (qualitatively) and measure the priority and meeting of identified needs (quantitatively).
- Is conducted within the specific socio-cultural context of Iran to inform locally relevant interventions.

This protocol outlines such a study, designed to generate the foundational evidence required to bridge the identified care gap and guide the future development of tailored, effective support programs for couples in the postpartum period.

### Objectives

The objectives of study will be based on the quantitative and qualitative phases as follows:

**Objectives of the first phase: Qualitative study**

1. Explaining the supportive care needs of Iranian couples during postpartum hospitalization from the perspective of couples, health care providers, and key informants such as managers and policymakers, etc.
**2.** Explaining the facilitators and barriers of supportive care for couples during postpartum hospitalization

### Objectives of the second phase: Designing interventional program

1-Identify the key supportive care needs of couples during postpartum hospitalization, drawing from insights gained in the qualitative phase using a panel of experts

2-Conduct a comprehensive literature review to find all available interventions to manage the couples’ supportive care needs identified in the previous step.

3-Design an intervention based on the postpartum supportive care needs of couples during postpartum hospitalization using a panel of experts

4-Assess the content validity of intervention (if content design is needed in the intervention, content validity is done by experts)

### Objectives of the third phase: Quantitative study

• Implementing, evaluating, and determining the effectiveness of the intervention designed to meet the couples’ supportive care needs during postpartum hospitalization

## Methods/Design

The present research is a multistage mixed study that will be conducted in a sequential exploratory manner. The collection/analysis of qualitative data is done before the quantitative phase. This study will be carried out in educational hospitals affliated to Kermanshah University of Medical Sciences, Kermanshah, Iran in 2024–2026.

### Ethics and publication

This study received ethical approval from the Ethics Committee of Tehran University of Medical Sciences, Tehran, Iran in 2024-02-27 (code: IR.TUMS.FNM.REC.1402.238). Sampling will be performed with the permission of the Research Administration of the Faculty of Nursing and Midwifery of this university. Any change in the protocol of the study will be subject to ethical approval. Data confidentiality will be guaranteed, participants will be ensured of voluntary participation in and withdrawal from the study, and informed consent will be obtained from all of them. This study was registered in the Iranian Registry of Clinical Trials (code: IRCT20110621006854N8). Study findings will be provided to participants and their families through healthcare centers and will be provided to healthcare providers and researchers through conferences and publication in academic journals.

First, an exploratory qualitative study will be conducted through a conventional content analysis to explain the couples’ supportive care needs during postpartum hospitalization.

Second, The findings from the qualitative phase directly inform the quantitative component. Specifically, the identified need categories are transformed into structured items and entered into a nominal group technique (NGT). In this phase, a panel of experts quantitatively rates and prioritizes the needs based on predefined criteria (e.g., importance, feasibility, and urgency). Then, with the main priority (set in the previous phase) in mind, the interventions and programs that aimed to meet the couples’ supportive care needs during postpartum hospitalization in Iran and other countries will be reviewed. In fact, the literature will be reviewed on interventions to meet the high-priority needs of couples in the postpartum period. Then, the related interventions and programs will be reviewed in another expert panel, with the research team and a group of interested experts. Accordingly, the most appropriate intervention will be developed.

Third, a quantitative study will follow, which aims to test the effect of the designed intervention on Iranian couples’ supportive care needs during postpartum hospitalization. It is noteworthy that the type of quantitative study and other related details will be decided on in this phase based on the type of intervention after prioritizing the needs and reviewing the literature. Having conducted the intervention, quantitative data will be analyzed in SPSS.

### Phase I: Qualitative study

In this phase, an initial qualitative study will be conducted with a conventional content analysis to explain the couples’ supportive care needs during postpartum hospitalization period.

### Participants in qualitative phase

The research population will be women receiving postpartum care in hospitals, their husbands, and care providers, womens’ at least one experience of childbirth (vaginal delivery or caesarean section) as well as postpartum care. Reproductive health service providers are midwives working in inpatient departments, gynecologists, resident doctors, psychologists, and nurses with at least two years of experience in serving the above-mentioned population, as well as managers and policy makers will be selected by through a logical and purposive sampling with maximum variety. The sampling and interviews will continue until data saturation.

### Inclusion criteria

The participants in this research will have the following characteristics:

- A couple with minimum level of literacy (reading and writing) and ability to communicate and interview
- Iranian nationality and ability to understand and speak Persian language
- Experience of at least one childbirth and postpartum care
- Being in the postpartum period (up to 15 days after normal vaginal delivery or caesarean section)
- Hospitalization in the postpartum ward of a hospitals affiliated with Kermanshah University of Medical Sciences

**Exclusion criteria**

- Couples with known mental illnesses and mood disorders
- Mother with any physical illness or complication during pregnancy and the postpartum period
- Fetal or infant death or abnormality in the current pregnancy
- Lack of willingness to participate in the study

### Sampling procedure

The participants will be selected purposefully to hold interviews with a maximum variety. The maximum variety involves the age group, level of education, socio-economic status, level of pregnancy, type of childbirth, baby’s sex, wanted or unwanted pregnancy, the time elapsed since childbirth, level of participant’s support, and so on in the reproductive health service provider sector, and the staff with different work experience. Purposive sampling means the researcher seeks to select participants experienced in the key concepts of interest to research. Interviews will be held at the time and place preferred by the participants, where the interviewer can be present wholeheartedly (e.g., hospitals affiliated with Kermanshah University of Medical Sciences, home, etc.).

### Data collection process

After the final approval of the research proposal by the post-graduate education council of the relevant specialized university and a scientific and ethical approval from Tehran University of Medical Sciences, a written consent will be gained from the vice-chancellor of education and the vice-chancellor of healthcare. The sampling will begin with the researcher visiting the hospitals affiliated with Kermanshah University of Medical Sciences. After contacting the participants, the researcher will introduce herself, explain the objectives of study, and decide on the time and place of interview. After clarifying the purpose of study, a consent form will be signed by all participants. We will use a deep semi-structured interviews to explain the couples’ supportive care needs during postpartum hospitalization period. To interview the participants, a friendly relationship will be established and they will be assured of the confidentiality of information they provide. The interviews will start with a number of open-ended questions. Some of the questions designed to ask the couple were as follows: “Describe your experience and feelings about the care you received after giving birth”; “What do you think should have been considered in this care?”; “What were your expectations from the doctor, nurse, and midwives?”. Initial questions to ask the healthcare staff included the following: “Can you describe a typical day in your role providing postpartum care?”; “What types of physical, informational, and emotional support and care do you offer during the postpartum period? In your opinion, is this support and care adequate, or is it lacking? Please explain.”; “What issues and concerns related to couples’ health arise during the postpartum period, and what are the reasons for these issues? Please elaborate”. Next, based on the initial answers and based on the interview guide, supportive care needs during postpartum hospitalization period will be enquired about and suggestions will be made to meet those needs. Moreover, when necessary, probing questions will follow such as “What do you mean?” or “Can you please explain more?”. According to the interview guide, simple and more general questions are asked at the beginning, According to the participants’ answers and experiences, the interview continues with more detailed questions. The interview questions are flexible, and if needed, new questions will be added to the interview guide. In the current research, during the interviews, observation and note-taking will be used to examine how the service providers act and interact with women who have given birth as well as with their husbands. Attention will be paid to the interviewees’ movements, emotional and behavioral reactions and the immediate environment. At the end, the recorded interviews and the notes taken are analyzed. The interviews will be recorded and then transcribed verbatim after gaining permission from the participants and ensuring that all interviews are confidential. To substantiate the validity, the interview transcripts will be given to the participants to read and confirm the content.

### Data analysis

In the upcoming study, a conventional content analysis will be used to analyze data. In this method, the data are collected directly from the participants and the categories are not determined in advance, yet are extracted from the textual data. Therefore, the data analysis will begin with recurrent readings of all data so that the researcher be immersed in the data and get an overview. Then the data will be read verbatim to highlight the words within the text that contain the key concepts and, thus, extract the codes. Then the researcher jots down the first interpretations of the text and does the initial analysis. As this process continues, the labels emerge for codes that reflect more than one key concept and are usually taken directly from the text and later become the initial coding map. The codes are then placed into categories based on how the different codes are related to each other. These emergent categories are used to organize and group the codes into meaningful clusters. Ideally, the number of categories is between 10–15, so that it can be big enough to cover a large number of codes [33]. A larger number of categories will be combined into a smaller number of sub-categories based on their interrelationship. A tree diagram will help organize these categories into a hierarchical structure. Then definitions will be created for each category, subcategory and code. When reporting the findings, examples of each code and category of data will be provided [33]. In this study, after each interview, the transcription will be done as soon as possible and preferably on the same day as the interview. After each interview, the supervisors and advisors read the interview transcripts again. The resulting codes are managed in MAXQDA 24.

The conventional content analysis in this study will be done in eight steps as suggested by Zhang and Wilmos (2016):

1- Preparing data for qualitative content analysis
2- Deciding on the unit of analysis
3- Doing the classification
4- Testing the coding in a sample of text
5- Extending the coding test process to the entire text
6- Achieving coding stability
7- Drawing conclusions from categorized or coded data
8- Reporting the results

The next step involves testing the codes and their consistency in the text through reviewing the codes and the majority of research team members’ agreeing on the codes. To check the consistency of coding, two experienced experts outside the research team will check the codes assigned to the categories and sub-categories. The next step is to make conclusions about the correct categorization of data and given codes, and to analyze the characteristics and relationships within and between categories. Each category and sub-category will be analyzed in the interview transcripts and finally the formed categories will be interpreted and reported [34,35].

### Trustworthiness and reliability of qualitative data

To ensure the trustworthiness (rigor or goodness) of findings, the four criteria (credibility, dependability, transferability and confirmability) proposed by Streubert and Carpenter (2011) [36] will be used.

### Credibility

In the present study, the researcher will take the following steps to check the credibility of findings.

1- Deep interviews are conducted at different times and places.

2- A combination of several data collection methods will be used such as interviews and field notes.

3- On the two ends of the range of research population, the participants will be selected with a maximum variety of age, social, economic and cultural status.

4- The review method will be used by the participants to confirm the credibility of extracted data and codes or to modify them. After coding each interview, it will return to the participants to ensure the credibility of codes and their interpretations and to correct the codes that do not represent their point of view.

5- The data will be checked by supervisors, advisors and experts to match and ensure that the categories match the participants’ statements.

6- The researcher’s ideas and presuppositions are specified in advance to prevent their impact on data analysis.

### Dependability

In this study, to test the dependability of findings, an external observer will be consulted to assess the possible similar cases and settle down the points of departure with the researcher. The primary codes (extracted from the participants’ experiences) will be interpreted, examples of how to extract themes and excerpts from the interviews will be provided for each theme.

### Transferability

To test the transferability of findings, the results will be presented to individuals who did not participate in the research. They are to judge the existence of similarities between the research findings and their experiences. Also, expert reviews will be used to serve this purpose.

### Confirmability

To test the confirmability of findings in this research, the transcripts of a number of interviews, extracted codes and categories will be provided to the researcher’s colleagues and members of the implementational board who are familiar with the nature of qualitative research and have not participated in the research. They will be asked to confirm the accuracy of the data coding process.

### Phase II: Intervention development

In the second phase of study, an intervention will be designed to explain Iranian couples’ supportive care needs during postpartum hospitalization in two parts:

#### *Part I: panel of experts* (formation of nominal group) to prioritize the needs explained in the qualitative part of study.

To design the intervention and link the qualitative and quantitative phases of study, the couples’ issues and supportive care needs will be prioritized in the presence of reproductive health experts, reproductive health policy makers, supervisors, and reviewers in a nominal group.

According to the supportive care needs explained in the first phase of study, the most important relevant needs will be prioritized by the research team and a group of experts and health planners (policy makers, reproductive health service providers, supervisors, advisors, etc.). NGT is used to generate ideas and reach a consensus. It is a well-structured brainstorming process that encourages all group members to share their ideas equally. This decision-making technique can generate ideas, solve problems or make decisions [37]. A facilitator asks participants to individually identify ideas and contribute to the creation of a list in response to a specific question, thus preventing participants from dominating the discussion and allowing everyone in the group to express their opinions equally [38].

In this study, at the beginning of the meeting, after an introduction to the purpose of meeting, the results extracted from the qualitative part of study will be presented. Then, the invited guests will be asked to jot down their opinions about the couples’ supportive care needs during postpartum hospitalization. Then a list will be made of the ideas of each group member in a table to share. Finally, the ideas will be voted and the most common needs will be picked out.

### Part II: Literature review

In order to develop the intervention, a review will be made of the existing national and international interventions and programs to meet couples’ supportive care needs. The literature on couples’ top-priority needs in the postpartum period will be reviewed. To this aim, SID, Magiran, Iran Medex, ProQuest, Pubmed, Google Scholar Embase, and web of science will be searched along with specialized websites such as WHO, International Planned Parenthood Federation (IPPF), and United Nations Population Fund (UNFPA) between the years 2000 and 2023. All available interventions regarding our needs will extract in details. Then, we will design a table contain the name of interventions, methods, and results of those studies to present in the second panel of experts.

### Part III: panel of experts

In this stage, all extracted interventions and programs will be reviewed using another NGT to find the most appropriate intervention.

### Intervention design

Based on the result of NGT, the research team will design the intervention. Additionally, its validity and reliability will be established according to the specific type of research conducted.

### Phase III: Quantitative study

#### Type and direction of the quantitative study.

This clinical trial was registered in Iranian Registry of Clinical Trials with trial registration No. IRCT20110621006854N8 in 2024-11-16 (URL:https://irct.behdasht.gov.ir/trial/79987).The quantitative phase of the present mixed research will be conducted as a clinical trial with two groups, an intervention and a control based on the result of the previous phases of the study. This phase will aim to test the effect of the designed intervention on Iranian couples’ supportive care needs in the postpartum period before discharge of hospital. It is noteworthy that the type of quantitative study and other related details will be determined in this phase of study based on the type of educational intervention after prioritizing the needs and reviewing the literature.

### Design

The clinical trial will have the two intervention and control group, which will be randomly selected from the hospitals of Kermanshah University of Medical Sciences. The current protocol has been structured in accordance with the “Standard Protocol Items: Recommendations for Interventional Trials” (SPIRIT) checklist [39].

### Date of the study

The first participant will be recruited in January 2026.

### Sample size

The sample size will be calculated based on the type of selected intervention. Consistent with the principles of exploratory research, we intentionally avoid premature assumptions; including fixed a priori numerical specifications for sample size. The primary aim of this study is to inductively elucidate Iranian couples’ supportive care needs. Predetermining a definitive quantitative sample before the exploratory phase is completed would impose confirmatory assumptions on an inherently inductive design and risk compromising methodological coherence. Therefore, rather than specifying a fixed number at this stage, we delineate the statistical framework that will guide sample size determination once the qualitative phase provides the necessary empirical parameters.

The exploratory findings will identify: (1) the primary outcome(s), (2) the appropriate unit of intervention (mother, father, or couple), (3) plausible effect sizes, (4) variance estimates, and (5) relevant correlation structures. Depending on these findings, three analytical scenarios are anticipated:

1. Individual-Level Intervention (Mother-Only or Father-Only): If the intervention is delivered and analyzed at the individual level, sample size for continuous outcomes will be calculated as:


$$\begin{array}{c} n=\frac{\text{2}\left(z_{\text{1}-\frac{a}{\text{2}}}+z_{\text{1}-\beta }\right){\text{2}\sigma }^{\text{2}}}{\Delta^{\text{2}}} \end{array}$$

or, using standardized effect size:


$$\begin{array}{c} n=\frac{\text{2}{\left(z_{\text{1}-\frac{a}{\text{2}}}+z_{\text{1}-\beta }\right)}^{\text{2}}}{d^{\text{2}}} \end{array}$$

For repeated-measures designs analyzed via mixed-effects models or GEE:


$$\begin{array}{c} n=\frac{\text{2}{\left(z_{\text{1}-\frac{a}{\text{2}}}+z_{\text{1}-\beta }\right)}^{\text{2}}\sigma^{\text{2}}(\text{1}-p)}{{m\Delta }^{\text{2}}} \end{array}$$

where (m) denotes the number of measurements and (*p*) the within-subject correlation. Parameters will be derived from exploratory estimates.

2. Couple-Based (Dyadic) Intervention

If supportive care needs are conceptualized as inherently relational, the couple will constitute the unit of analysis. In this case, within-dyad correlation will be accounted for using a design effect:


$$\begin{array}{c} \text{DE}=\text{1}+(\text{k}-\text{1})\text{IC}{\text{C}}_{\text{dyad},}k=\text{2} \end{array}$$

with the adjusted sample size:


$$\begin{array}{c} {\text{n}}_{\text{adj}}=\text{n}\times \text{DE} \end{array}$$

The dyadic intraclass correlation coefficient will be informed by exploratory findings.

3. Cluster-Level Intervention

Should organizational determinants (e.g., ward or hospital factors) emerge as central, cluster randomization may be required. The design effect will then be:


$$\begin{array}{c} \text{DE}=\text{1}+(\text{m}-\text{1})\text{IC}{\text{C}}_{\text{cluster}} \end{array}$$

where (m) is the average cluster size. The intracluster correlation will be estimated based on exploratory evidence and relevant literature.

#### Methodological justification.

The qualitative phase is epistemologically foundational to this study. It defines construct boundaries, determines the appropriate intervention unit, and generates the statistical parameters required for valid power analysis. Accordingly, the final quantitative sample size will be calculated only after empirically grounded estimates are obtained, ensuring methodological rigor while preserving the integrity of the exploratory sequential design.

### Settings

**Fig 1:**
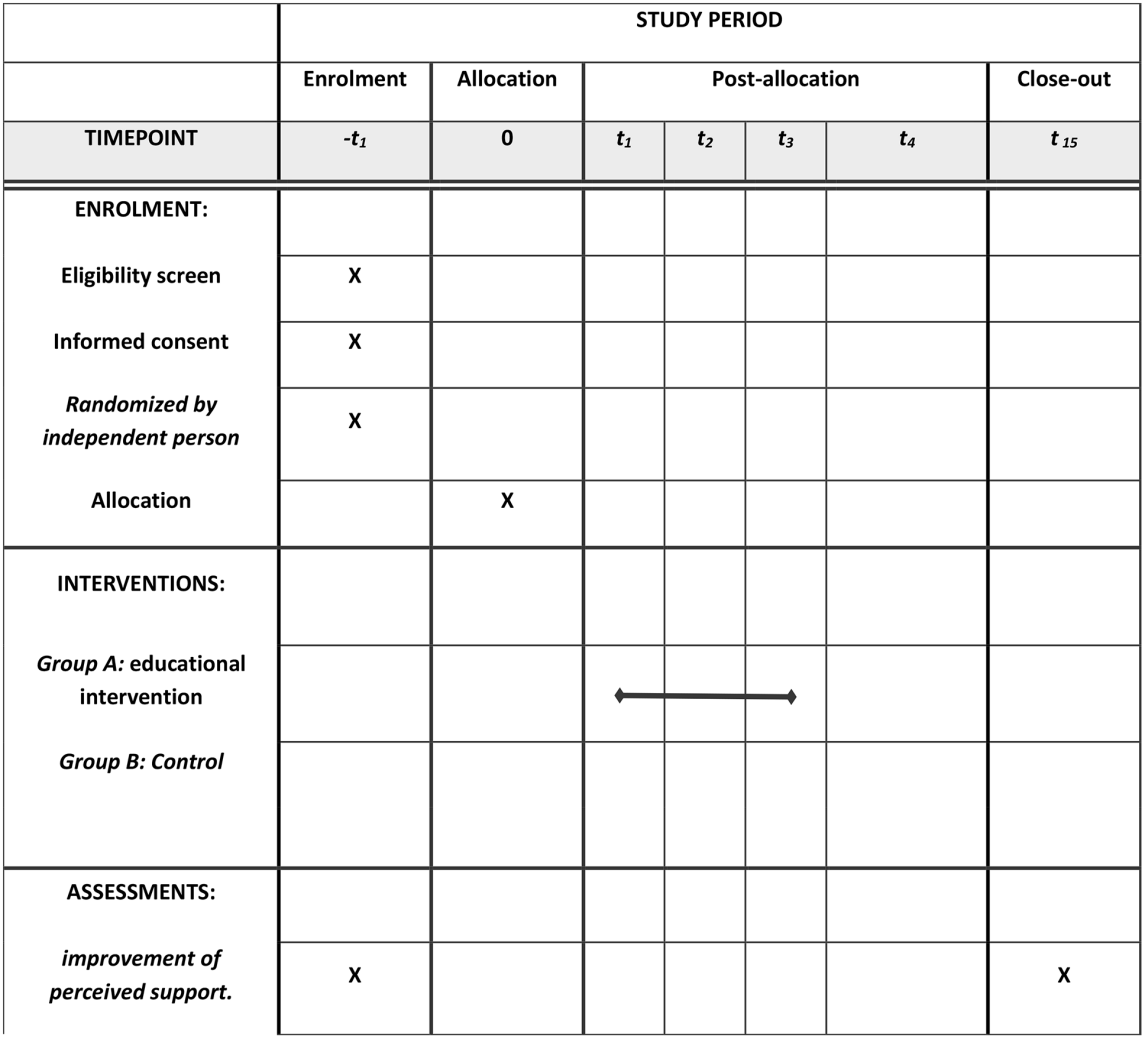
Spirit schedule of enrolment, interventions, and assessments.

The research environment in this study will be selected hospitals under the supervision of Kermanshah University of Medical Sciences. All post-partum women who are eligible to enter the study were included in the study after providing full explanations about the purpose of the study and obtaining informed written consent. And finally, they were randomly assigned to two intervention and control groups. Fig 1 shows the Spirit schedule of enrolment, intervention, and assessment.

### Participants/Inclusion and exclusion criteria

Inclusion criteria will be women aged 18–49 with Iranian literacy, can read and write, are healthy, have experienced at least one delivery, and who are in the postpartum period. Exclusion criteria will be women who have a known history of mental illness and mood disorders or the death of a fetus or baby during the recent delivery process or if the baby has a known abnormality, or if they have had a history of an accident in the past 6 months, they will not enter.

### Intervention groups

Consisting of the intervention group (education and counseling in the field of the needs of the postpartum period) and the control group. The control group received only routine interventions.

### Outcome

The main outcome of the study will be improvement of perceived support.

### Randomization and Allocation Conceral

To minimize selection bias and ensure comparable groups, a randomization procedure with strict allocation concealment will be implemented.

**1-Sequence Generation**

An independent statistician, not involved in recruitment or intervention delivery, will generate the allocation sequence. A blocked randomization method with varying block sizes (e.g., 4, 6) will be used to ensure balanced group sizes throughout the enrolment period. The sequence will allocate participants to either the Intervention Group or the Control Group.

**2-Allocation Concealment**

The sequentially numbered, opaque, sealed envelope will be used to conceal the allocation sequence until the moment of assignment. Based on the generated sequence, the independent statistician will prepare a set of consecutively numbered, opaque, and sealed envelopes. Each envelope will contain a card specifying the assigned group. The envelopes will be delivered to the principal research coordinator at the study site.

**3-Implementation**

After a post-partum woman is confirmed eligible and provides written informed consent, the enrolling research assistant (who will be blinded to the sequence) will open the next consecutively numbered envelope in the presence of the participant. This action irrevocably assigns the participant to the group indicated on the card inside. The enrolling assistant will then inform the relevant intervention provider.

**4-Blinding**

Due to the nature of the educational interventions, participants and intervention providers cannot be blinded to group assignment. This is an inherent limitation of the trial design. However, to minimize assessment bias, outcome assessors and data analysts will be kept blinded to group allocation throughout the study. The potential for performance and detection bias resulting from the lack of participant/provider blinding will be explicitly acknowledged in the study limitations.

### Data instrument

After successfully passing both the qualitative assessment and the expert panel review, we will select the appropriate instrument based on the chosen intervention method.

### Data analysis

Following the coding process, the gathered data will be securely stored on password-protected computers to maintain confidentiality. Data will be entered into the SPSS software (version 23.0) by trained personnel using assigned codes to maintain blinding. To ensure accuracy, double data entry will be performed, with two independent personnel entering the same data and discrepancies resolved by the primary investigator. Because randomization occurs at the cluster level and the intraclass correlation coefficient (ICC) is expected to inflate standard errors, analyses will use linear mixed-effects models (for continuous outcomes) or generalized linear mixed models (for non-normal outcomes). The basic model will include random intercepts for clusters to capture between-center variability. Covariates will include baseline perceived support, maternal age, education, parity, and other a priori relevant factors. If the ICC is negligible, a generalized estimating equation (GEE) with an exchangeable correlation structure will be considered as a sensitivity analysis, recognizing its limitations with a small number of clusters.

Analysis of covariance (ANCOVA) will be used to compare groups at follow-up, adjusting for baseline values and predefined covariates. Specifically:

- Primary model: follow-up outcome = group + baseline outcome + baseline perceived support + maternal age + education + parity + site (center) as a random effect (if clustering is retained in the analysis) or as a fixed effect if appropriate.
- Covariates to adjust (pre-specified): baseline value of the outcome, baseline perceived support, maternal age, education, parity, and any other a priori relevant factors collected at baseline.
- Interaction terms: test for a Group × Time interaction if there are repeated measures; otherwise focus on a single post-intervention follow-up ANCOVA.
- Assumptions and checks: assess linearity between covariates and the outcome, homogeneity of regression slopes, and normality of residuals. If assumptions are violated or the outcome is non-normal, consider a transformation or a generalized linear model with an appropriate link (e.g., Gaussian with identity link for continuous outcomes, or a different GLM as warranted).

## Discussion

This study aims to explain the supportive care needs of couples in the postpartum period through mixed mehod approach. By foregrounding the voices and experiences of both mothers and fathers, this research will generate a nuanced framework of their requirements.

The postpartum period is critical for the long-term health of mothers, infants, and families. Despite its importance, global maternal mortality remains unacceptably high, with more than one-third of deaths attributable to postpartum complications [40,41]. While facility-based childbirth has improved the management of immediate life-threatening conditions like hemorrhage and sepsis, it often fails to address the comprehensive and ongoing physical, mental, social, and emotional needs of couples transitioning to parenthood [41].

A significant proportion of women report dissatisfaction with postpartum care, perceiving it as insufficient and fragmented [8–11]. This inadequacy stems from multifaceted barriers within health systems, including a predominant technocratic (biomedical) model of care that neglects psychosocial support, lack of respectful and individualized care, insufficient patient engagement in decision-making, and systemic issues related to insurance, policies, and socio-cultural contexts [21,22,41]. Consequently, there is a well-documented gap between the provision of postpartum services and the experienced needs and expectations of new parents [13,42].

Current literature extensively outlines systemic problems but provides limited insight into the specific, nuanced support needs as articulated by couples themselves within specific cultural settings. To address this complex issue comprehensively, a mixed methods research design is essential. This approach allows for a deeper and more complete understanding than either qualitative or quantitative methods alone can provide [43]. The qualitative component will explore the depth, context, and meaning of couples’ experiences and needs, while the quantitative component will assess the prioritization and selection of the best intervention of meeting these needs across a broader population.

## Supporting information

S1 File Mixed Methods Appraisal Tool (MMAT), version 2018. (PDF)

S2 File Fillable-SPIRIT-Outcomes-2022-Checklist-with-SPIRIT-2013. (PDF)

S3 File Protocol document in Persian language. (DOCX)

S4 File Protocol document in English language. (PDF)

## Abbreviations

WHO: World Health Organization
NGT: nominal group technique
UNFPA: United Nations Population Fund
IPPF: International Planned Parenthood Federation
