# Supplementary material for: The supportive care needs of Iranian couples during postpartum hospitalization: A protocol of design, implementation and evaluation of intervention
Source: PLoS One. 2026 Jun 3;21(6):e0350038. doi: 10.1371/journal.pone.0350038 (PMC13232809; doi:10.1371/journal.pone.0350038)
Supplement: S3 File — (DOCX) [file pone.0350038.s002.docx]

**Title :** Exploring the supportive care needs of Iranian couples during postpartum period: Designing, Implementing and Evaluating the Intervention

**Tracking Code:** 70979

**Researcher :** Zahra rastad

**Specialty:** -

**Project Code:** 40111151006

**Ethics Code:** IR.TUMS.FNM.REC.1402.238

**Date of Registration:** 2024/02/01 17:51:02

**Postage date:** 2024/02/01 22:15:03

**Edit date:**

**The second target center:** School of Nursing and Midwifery

**The second target center:**

**The third target center:**

General specifications and design abstract

**English Title**

Exploring the supportive care needs of Iranian couples during postpartum period: Designing, Implementing and Evaluating the Intervention

**Educational level**

Ph.D Thesis

**Student number**

40111151006

**KEYWORDS**

Supportive care needs - couples - postpartum period

**Thesis type**

Basic-Applied

**Type of study**

other

**Summary of the Necessity of Thesis Implementation**

The postpartum period is marked by significant physiological and hormonal changes, presenting mothers with social, emotional, and functional challenges. Factors associated with women’s dissatisfaction with postpartum care often reflect their lived experiences. Key root causes of dissatisfaction include the sensitivity of caregivers, the extent to which women’s concerns are taken seriously, the haste in delivering care by staff, the length of hospital stay after childbirth, and the lack of counseling and supportive care measures. These factors are considered major barriers to achieving optimal health in perinatal and postpartum services.

Given the need for precise planning and new evaluations to ensure the adequacy of postpartum care, approaches must be adopted to comprehensively address the challenges faced by parents in accessing holistic postpartum care. This should be done through a supportive and comprehensive care approach, rather than limiting care to the brief services provided to women after hospital discharge. This necessitates qualitative research to identify the needs and challenges of couples during this critical period.

Moreover, since no qualitative research on this topic has been conducted in Iran to date, and previous studies have only quantitatively assessed the quality of postpartum care, this study aligns with the research priorities of the Midwifery and Reproductive Health Department at Tehran University of Medical Sciences. The study aims to design, implement, monitor, and evaluate interventions to reduce maternal and neonatal morbidity postpartum. Using a mixed-methods approach, it seeks to explore the supportive care needs of couples during the postpartum period and design a needs-based intervention to enhance postpartum health services.

**Summary of the Methodology and Analysis Approach**

The present research is a multi-stage mixed-methods study, consisting of three phases: qualitative, intervention design, and quantitative. It will be conducted during the academic year 2023-2024 at Kermanshah University of Medical Sciences. The study population includes postpartum women receiving care in the maternity wards of hospitals affiliated with Kermanshah University of Medical Sciences (who have experienced either vaginal delivery or cesarean section), women visiting healthcare centers within eight weeks postpartum, their spouses, reproductive healthcare providers, managers and policymakers in the fields of reproductive and women's health, psychologists, and midwives working abroad.

***First Phase: Qualitative Study***

An exploratory sequential qualitative study will be conducted using conventional content analysis to identify the supportive care needs of mothers and fathers during the postpartum period. The qualitative research will employ a content analysis approach, beginning with semi-structured in-depth interviews with women, their spouses, and healthcare providers. Sampling will be purposive, with maximum variation in terms of age, pregnancy rank, occupation, type of delivery, education level, etc. The researcher will use conventional content analysis, following the method proposed by Graneheim and Lundman, to categorize the data.

***Second Phase: Intervention Design***

Based on the findings from the qualitative phase, the supportive care needs and issues of women and their spouses will be prioritized in a session involving reproductive health specialists, policymakers, supervisors, and reviewers, using the nominal group technique. Subsequently, a literature review will be conducted to identify an appropriate intervention for the quantitative phase of the study.

***Third Phase: Quantitative Study***

In the quantitative phase, an evidence-based and needs-driven intervention will be designed, guided by the qualitative data, the priorities identified in the nominal group session (serving as the link between the qualitative and quantitative phases), and an extensive literature review. The designed intervention will then be implemented and evaluated to address the supportive care needs of mothers and fathers during the postpartum period. Finally, the quantitative data will be analyzed using SPSS software**.**

Record

**General, Specific, and Practical Objectives**

**Main Objectives of the Project:**

1. Identifying the supportive care needs of Iranian couples during the postpartum period.

2. Designing, implementing, and evaluating an intervention based on the most critical supportive care needs of Iranian couples during the postpartum period.

**Objectives of the Qualitative Study:**

1. Identifying the supportive care needs of Iranian couples during the postpartum period.

2. Identifying the supportive care needs from the perspective of service providers (physicians, midwives, nurses, etc.) during the postpartum period.

3. Identifying the supportive care needs from the perspective of experts (key informants such as managers and policymakers).

4. Identifying the facilitators and barriers to supportive care for couples during the postpartum period.

**Objectives of the Quantitative Study:**

1. Determining the most critical supportive care needs of couples during the postpartum period based on the results extracted from the qualitative study and literature review, using expert panels.

2. Designing an intervention based on the supportive care needs of couples during the postpartum period.

3. Determining the content validity of the designed intervention (if content design is required in the intervention, content validity will be assessed by specialists).

4. Implementing and evaluating the intervention based on the supportive care needs of couples during the postpartum period.

These objectives will be further refined in subsequent stages.

**Practical Objectives of the Research:**

The intervention designed in this study, which is based on the supportive care needs of Iranian couples during the postpartum period, will be provided to policymakers and health managers in case of its effectiveness, to be used in improving the quality of postpartum care.

**Introduction – Problem Statement**

The postpartum period is characterized by significant physiological and hormonal changes, which can present mothers with social, emotional, and functional challenges (1, 2). Although motherhood is a unique and often joyous experience, the transition to motherhood can also be accompanied by stress and tension. This is due to the rapid and numerous intra- and interpersonal changes across various dimensions that women must adapt to during this time (3).

During the postpartum period, women may face a range of risk factors, including physical and biological (poor physical health), psychological (postpartum depression and grief, stressful life events), childbirth and infant-related factors (unintended pregnancy, emergency cesarean section, stress induced during natural childbirth, and birth trauma), socio-demographic (young maternal age), and cultural factors (low social support) (4). At the same time, they need to share their experiences of this life stage with others and receive reassurance that they are understood (4). Identifying the supportive needs and expectations of new mothers is crucial for their well-being and recovery during the postpartum period. Supportive needs, which address various physical, cognitive, emotional, and psychological aspects of individuals in extraordinary and unique life circumstances, are the primary focus of woman-centered care and aim to improve the quality of life for women postpartum. Key dimensions of supportive care include pain and symptom management, emotional and psychological support, provision of necessary information, improved communication, decision-making ability, addressing practical needs (financial concerns, access to resources, etc.), respect for dignity and autonomy, and the delivery of coordinated and integrated care (5).

The concerns of women in the postpartum period encompass universal themes such as anxiety, fear, feelings of loneliness, worries about their competence and ability in parenting and accepting the role of motherhood, negative body image, the ability to establish satisfying sexual relationships, and rebuilding relationships with their spouse and other family members, among other important issues. These concerns are notably more pronounced and significant in first-time mothers compared to those who have previously experienced motherhood. These themes are reflected in national and international evidence and literature (1, 2, 6-9).

The primary goal of optimal postpartum care is to preserve and promote the health of the mother and her newborn, and to create an environment that supports the family and community in addressing a wide range of health and social needs. It is essential that the content of postpartum care encompasses the concept of optimal health and aligns with all dimensions of health as defined by the World Health Organization (10).

However, evidence indicates that some women perceive the care they receive during their stay in the postpartum ward as inadequate. For instance, in a study conducted by Simbar et al. on women admitted to selected hospitals affiliated with Kurdistan University of Medical Sciences, the care provided was evaluated as weak in terms of psychological and emotional support from the women’s perspective (11). Similarly, in another descriptive study by Simbar et al., the quality of postpartum care in hospitals affiliated with Shahid Beheshti University of Medical Sciences was reported as poor (12). Ali Dosty et al., in their assessment of women’s satisfaction with postpartum services and facilities in the maternity ward of Hajar Hospital in Shahrekord, found that satisfaction with human services was low. They noted that the high workload of staff reduced their focus on providing better services (13).

Overall, factors associated with women’s dissatisfaction with postpartum care reflect their experiences. The sensitivity of caregivers, the extent to which women’s concerns are taken seriously, the rush in providing care by staff, the length of hospital stay after childbirth, and the lack of counseling and supportive care measures (14) are the most significant root causes linked to dissatisfaction. These factors are considered the main barriers to achieving optimal health in the context of birth-related and postpartum services (14).

In a review study aimed at examining women's perspectives on hospital-based postpartum care, women perceived postpartum care as having a lower priority compared to other types of care. (Postpartum care is not prioritized among the services provided by care providers) (15). Other issues raised by women in the studies included the overcrowding and occasional chaos in postpartum wards, as well as the lack of flexibility in addressing individual women's needs. Limitations such as insufficient staffing, a high number of visitors, restrictions on mothers holding and bonding with their newborns immediately after delivery (and in certain cases, prolonged hospitalization of the newborn), and supportive challenges such as the absence of counseling facilities for issues like adapting to changes in intimate and family relationships, resuming sexual activity, developing parenting skills, coping with birth trauma, and addressing concerns like genital injuries, body image, and others were cited as barriers to providing woman-centered care (15-22).

Additionally, it is rare for fathers to have the opportunity or facilities to stay overnight in the hospital with their spouse and newborn during the postpartum period (15, 16). They are also seldom allowed to participate in counseling or care discussions (to receive counseling care alongside their spouse). However, the postpartum period is a critical time for fostering parental collaboration in newborn care. Men desire greater involvement in childcare, but this requires support in their transition to fatherhood. Few studies have elaborated on what constitutes "support" for fathers, which may differ from the support mothers seek. Perinatal mental health issues occur at similar rates among men as they do among women, but men are often less engaged with healthcare services. As is well-established, poor mental health in fathers adversely affects the emotional and cognitive development of the child and their relationship with their partner. In fact, studies indicate that fathers require support across all levels of the ecosystem, including policy changes, socio-cultural aspects, and family and spousal support, particularly in healthcare services and care (23).

Other issues raised include the lack of access to high-quality professional care at the expected level, inconsistencies and incompatibilities in the advice provided by different caregivers, the absence of counseling facilities, and specialized care (24).

However, negative experiences or perceived gaps in care have not led women to want to leave the hospital earlier. Most women prefer to stay in the hospital until they are confident in their ability to care for their newborn. They use this time to learn skills related to breastfeeding and overall infant care, build their confidence in these areas, and exchange experiences with other women, especially multiparous women who have more expertise in these matters. They also seek necessary guidance from caregivers. Based on available evidence, addressing these issues and allocating sufficient time by caregivers is one of the main concerns of women in the postpartum period. In fact, women are eager to participate in classes and utilize post-discharge support services (e.g., breastfeeding clinics, mental health promotion centers, individual counseling, sexual counseling, etc.). Findings suggest that for many women, staying in the hospital for the days following birth seems to be the only safe option for themselves and their newborns (8, 9, 16).

There is a consistent perspective that the physical presence and availability of professional support and supportive care significantly reduce concerns and address the needs of women in all desired dimensions.

It appears that there are several factors that can contribute to women's perception of the hospital as the safest and most appropriate place after childbirth. These factors include the extent of comprehensive measures to address and respond to various dimensions of women's postpartum needs, as well as the induction of a sense of receiving adequate support from staff and service providers (16). Conversely, certain social factors can have a negative impact on women's perspectives. The primary factors revolve around the social infrastructure of childbirth in the country, the medicalization of the birth and pregnancy process, the technocratic (biomedical) model of the healthcare system, and the lack of women's participation and involvement in medical decision-making within the healthcare process (25).

The concept of going to the hospital to give birth and staying there postpartum has culturally evolved into a social norm, suggesting that childbirth is a dangerous, harmful, and uncontrollable process (26). The authors argue that this construction, linked to the medicalization of birth and the increasing erosion of women's sense of competence regarding childbirth and newborn care, has occurred particularly due to the lack of information institutionalized in the culture (27). Given this background, it seems entirely expected and natural for mothers to harbor fears about their own health and that of their newborn in the absence of expected medical supervision and support (27).

In fact, the manner in which care is provided by healthcare providers (particularly midwives) and their interactions with mothers after childbirth have created the perception among women that accessing hospitals with 24-hour medical support during and after childbirth is safe and secure. However, in practice, there is a significant gap between expectations and reality (26). What emerges from structured interviews with women in the postpartum period is that they do not find healthcare providers (midwives and nurses) sufficient to address their concerns and cannot rely on them to meet their needs. Measures such as the medicalization of childbirth may have led women to feel the need for healthcare professionals and doctors to be present around them to alleviate their anxieties or fears during the transition to motherhood and parenting (16).

Moreover, according to existing evidence, women prefer to receive healthcare services in settings where providers interact with them respectfully and deliver care optimally. From their perspective, respectful care includes supportive care tailored to their needs (24).

Their descriptions of disrespectful care include verbal or physical abuse or the imposition of a sense of dominance and power by the caregiver. Studies indicate that vulnerable women (adolescents, women with low socioeconomic status, and HIV-positive women) are more likely to receive disrespectful care (24).

Perinatal care provided by healthcare providers, given its somewhat invasive nature, has the potential to cause harm or retraumatize women (18). Maternity care often involves responding to sensitive questions, requiring clothing changes, and conducting examinations or procedures that may be perceived as invasive. These factors have the potential to trigger flashbacks and retraumatization. Such women may exhibit high levels of anxiety, emotional distress, and dissociation during maternity care. Additionally, given the high prevalence of disrespectful behavior, the need for supportive and soothing responses becomes inevitable (18, 28).

Harris and Ayers’ study also found that the strongest predictor of birth-related PTSD was interpersonal issues with healthcare providers and a lack of support (28). While providers may consider their actions and interactions routine, some women perceive them as harmful (28). This phenomenon has led to the introduction of the legal term “obstetric violence” in some countries. Walmar and colleagues noted that women used the term “birth rape” to describe their traumatic childbirth experiences. Qualitative studies examining women’s experiences of psychological trauma during childbirth highlight that the nature of interactions with healthcare providers is a more significant factor than medical interventions or the type of delivery (28).

Given the multiple realities and the needs highlighted, it can be concluded that the healthcare system in the country has relatively neglected the psychological and social support of mothers and fathers during the postpartum period. There is significant evidence of a mismatch between the healthcare services provided and the support mothers expect, with a persistent gap between what mothers anticipate in terms of care during and after childbirth and what is actually delivered. Additionally, studies specifically focusing on fathers in this context are notably scarce (29).

The American College of Obstetricians and Gynecologists (ACOG) recommends routine mental health screening as part of comprehensive postpartum follow-up. However, mood and emotional well-being are only one of the nine recommended areas for coverage and assessment during prenatal and postpartum visits. The other recommended areas focus on maternal and infant care and maternal physical health and recovery. Concerns related to maternal mental health and psychosocial experiences may not receive adequate time and attention. Moreover, mental health screening is not universally implemented and is sometimes conducted in ways that do not align with recommended guidelines (29).

Another issue is the need for flexibility in postpartum care. Women desire a variety of care options, particularly first-time mothers. Women who have given birth to their second or subsequent child may require less postpartum support and education. This perspective aligns with their increased experience and confidence in caring for subsequent children and their reduced need for continuous professional security and support. First-time mothers may not recognize or demand different postpartum care options, as their perspective is often based on the assumption that "what is available" is the best option. In contrast, women who have previously given birth express higher expectations regarding the diversity, coverage, and adequacy of supportive care in various dimensions. This underscores the need for flexibility in postpartum care options, recognizing that women’s needs may vary based on parity and prior experiences (30). To address these needs, various models of postpartum care have been implemented worldwide. For example: In Australia, hospitals are required to ensure adequate postpartum care for women, infants, and their families based on clinical and psychosocial needs. This includes at least one home visit or another form of follow-up contact for all women after hospital discharge; at least two or more home visits for women and infants with specific and diverse needs, such as women with substance abuse issues, recent immigrants, young single mothers, and women with disabilities. Additional home visits are provided for women with complications arising from childbirth immediately postpartum or after discharge, particularly to address family concerns and provide other supportive care (31).

Similarly, in Switzerland’s healthcare system, the shortened hospital stay after childbirth is compensated by home visits conducted by community midwives. Women and families can receive up to 16 visits from self-employed community midwives within the first 56 days postpartum for their first child or in complex conditions, and up to 10 visits for subsequent children. The average number of visits in 2018 was 7.5. During these home visits, midwives assess the physical health of women and infants and provide personalized support on breastfeeding, infant care, and mental health (32).
In the Netherlands, where a system of maternity care assistants exists to support women for up to eight days, women may feel more confident in choosing to return home earlier. Currently, in countries such as Iran, such a system for implementation does not exist and requires the development of an evaluated framework as well as changes in funding mechanisms (33).

The issue of inflexible length of stay that does not align with women’s needs is an important topic of discussion. A decade ago, the UK Audit Commission warned that the time women spend in the hospital should not be predefined or standardized. Instead, it suggested that policies should be flexible and emphasized the necessity of consulting women regarding the length of stay and the nature of care they receive (34). It is crucial that any move toward a shorter postpartum stay be based on an assessment of the physical and mental health of both the mother and the newborn, as well as the mother’s satisfaction with the care received.
Apart from developments in clinical practice, reducing the average length of hospital stay is considered a way to achieve efficiency and cost-effectiveness. Indeed, shorter stays reduce the cost per patient. Early discharge after childbirth appears to be safe in controlled studies with post-hospital follow-up programs. In this regard, several countries, such as France and the UK, have reduced their bed capacity in postnatal services and instead focused on post-discharge care. In Switzerland, postnatal visits are fully reimbursed under the mandatory health insurance scheme outlined in the Swiss law LAMAL, thus ensuring near-universal access to services. Nurses and social care professionals may provide additional services during this period. When families experience social distress, suffering from loneliness, poverty, inadequate housing, or a general lack of support, community midwives tend to offer more extensive services. They coordinate with other professionals, such as social workers, but also take direct action when necessary (35).

If alternative arrangements for postnatal care are introduced, and comprehensive supportive care programs with full coverage for women are adopted, it is possible to ensure that the needs of women during the critical postpartum period are met. This requires conducting assessments, examining concerns, and exploring the various dimensions of women’s postpartum challenges from their own perspectives, as well as evaluating the impact of such programs on the health of women and infants, parental anxiety and confidence, and the economic implications for women’s health services and their families (16).

Since precise planning and new evaluations are necessary to ensure the adequacy of postnatal care, approaches must be adopted that fully address the challenges parents face in accessing comprehensive postnatal care, with a focus on holistic supportive care. Care should not be limited to the minimal services provided to women after hospital discharge (16). This necessitates qualitative research to elucidate the needs and challenges of couples during this period. Moreover, given that no qualitative research on this topic has been conducted in Iran to date, and previous studies have only quantitatively assessed the quality of postnatal care, this study, in line with the research priorities of the midwifery and reproductive health department at Tehran University of Medical Sciences—which focuses on designing, implementing, monitoring, and evaluating interventions to reduce maternal and neonatal morbidity after childbirth—aims to use a mixed-methods approach to explore the supportive care needs of couples in the postpartum period and design and implement a needs-based intervention to enhance postnatal healthcare services. If the designed intervention proves effective, it will be made available to policymakers and health managers to improve the quality of postnatal care.

**Literature Review**

**Quantitative Studies**

1- Darakhshanpour et al. conducted a clinical trial titled "The Effect of a Psychological Educational Intervention on Postpartum Health" on 80 pregnant mothers hospitalized in the maternity ward of Shahid Sayyad Shirazi Hospital, Gorgan. The subjects were randomly divided into two intervention groups (40 people) and control groups (40 people). The subjects in the intervention group and their spouses received postpartum mental health education 48 hours before the estimated date of delivery. The control group did not receive any education. Data were collected using a demographic questionnaire and the General Health Questionnaire-28 (GHQ-28) at baseline and in the second and fourth weeks after delivery. The data were analyzed using SPSS software (version 18) at a significance level of 0.05. The mean age in the intervention group was 26.3±5.6 years and in the control group was 27.9±5.1 years. There were no significant differences between groups in terms of age, pregnancy order, number of miscarriages, type of delivery, and history of addiction at baseline. The GHQ-28 total score decreased significantly from baseline to the fourth week postpartum in both groups (P=0.001). There was no significant difference between groups in terms of reduction in the GHQ-28 total score (P>0.05). There was also no significant difference between groups in terms of change in GHQ-28 subscale scores over time.

The findings of this study suggest that a short-term educational intervention 24 to 48 hours before delivery is not effective in improving mental health in the postpartum period. Therefore, educational interventions should be provided for a longer period, a few weeks before or after delivery in the form of supportive care. (36).

2- Chen et al., in a study titled "Effects of a Support Group Intervention on Postpartum Distressed Women: A Controlled Study in Taiwan," randomly assigned 60 postpartum anxious women to two support groups (n = 30) and control groups (n = 30). Women assigned to the support group participated in four support group sessions that included discussions about transitioning to motherhood, postpartum stress management, communication skills, and life planning. Beck Depression Inventory (BDI) and Perceived Stress Scale (PSS) scores decreased in those who participated in the support sessions, and Interpersonal Support Inventory (ISEL) scores also increased at the end of the fourth weekly session. In contrast, no significant change was observed in the control group during this period. This controlled study provides evidence that participation in support groups provides measurable psychosocial benefits for postpartum anxious women.

This study has similarities with the present study in that it addressed women's concerns in the postpartum period, made these themes the focus of support group sessions, and addressed the topic of postpartum support. Therefore, it will be used in the discussion and analysis of the results (37).
3-Glavin et al. conducted a clinical trial entitled "Supportive counseling by public health nurses for women with postpartum depression". The study population consisted of postpartum women living in two municipalities in Norway who had given birth to a live child between June 2005 and December 2006. A total of 228 women were included in the study. Public health nurses in one of the municipalities were trained in the identification of postpartum depression and in providing supportive counseling. Pre-testing was performed using the Edinburgh Postpartum Depression Scale at 6 weeks postpartum. Post-testing was performed using the same scale at 3 and 6 months postpartum. The results showed that the depression score in the experimental group was significantly reduced compared to the comparison group at 3 and 6 months postpartum. The researchers noted that supportive counseling provided by public health nurses is an effective treatment for postpartum depression. Further research is needed to manage postpartum depression in primary health care.

This study, since it deals with supportive counseling in its intervention, which is one of the gaps in care in our country, is important and worthy of attention, so it will be used and analyzed in the design of the intervention and discussion. This study differs from the present study (healthy individuals) in that it considers people with postpartum depression as the target group of the study (38).

4- Kocak et al. conducted a study titled "Development of a postpartum mobile phone support application and the effect of the application on symptoms of maternal anxiety and depression" between July 2017 and February 2020, which was a pre-test-post-test controlled trial, in which those who had healthy infants constituted the study group. Data were collected using a demographic information form, the STAI State and Persistence Anxiety Scale, and the Edinburgh Postpartum Depression Scale. Mixed ANOVA analysis, t-test in dependent groups, t-test, and chi-square analysis in independent groups were used to analyze the data. The findings indicated that most mothers who used this software exclusively breastfed their babies and felt adequate about breastfeeding. The depressive symptoms of mothers who used the postpartum mobile phone support program were lower than those of mothers in the control group. However, it was found that this program alone was not sufficient to reduce anxiety levels and depressive symptoms (P>0.05).

This study is noteworthy in terms of using new methods of post-discharge follow-up (mobile phone application) and will therefore be discussed and examined in the intervention design (39).

5- Baqerzad et al., in a clinical trial entitled "The effect of home care on husband's support during the postpartum period", randomly assigned 64 women who had given birth in Isfahan hospitals to two intervention and control groups. A researcher-made questionnaire was used to collect data and the validity and reliability of the questionnaire were evaluated. Descriptive statistics, independent t-test, Fisher's exact test, chi-square, and Mann-Whitney U test were used to analyze the data. The significance level was considered to be less than 0.05. In the results section, the researchers noted that after receiving home care, husband support in the intervention group was significantly different from the control group (p=0.001). There was also a significant difference between the intervention and control groups in the areas of trust in the wife, trust in her, attention to her care needs, financial support, trying to solve her problems, buying gifts, and helping to care for the child (P<0.05). According to the results of this study, the home care program can effectively increase husband support, which leads to a safe postpartum period. Also, providing home services requires careful and appropriate planning and management by midwives.

This study is noteworthy and valuable in that it addresses the dimensions of social support and care by the spouse as an intervention, and unlike other studies, it targets the dimension of care at home by family members, which will be used in the intervention design of the present study (40).

6- Fenwick et al. (2010) in a study entitled “Western Australian women’s perceptions of the style and quality of postnatal midwifery care in hospital and at home” used a self-report cross-sectional survey to describe the different dimensions of informational, practical and emotional support provided by midwives in the postpartum period. A questionnaire specifically designed for the study population was completed by 2699 women in the first 8 weeks after delivery. . Data were analyzed using descriptive statistics, t-test and chi-square. The results of the study showed that women were satisfied with most aspects of midwifery care related to the provision of practical advice and assistance regarding newborn care and their physical recovery. The areas that received less positive ratings were related to the provision of ongoing advice, availability of midwives, emotional care, immunisation and contraception, and provision of information about maternal health needs. The researchers reported that primiparous mothers generally rated the style and quality of care lower than multiparous mothers, and that there was a trend toward private hospital care among women who rated midwifery care less favorably. In their study, home-based midwifery care was rated very positively, significantly better than hospital care. Although the majority of women in this study were satisfied with the components of physical care and the provision of information and assistance with feeding, sleeping, and settling the baby in the short term, there was less satisfaction with emotional care and preparation for life at home with a new baby and adjustment to parenting (41).

This study adds to our understanding of women's experiences and expectations in the early postpartum period and provides information on which improvements can be made in postpartum services and care, which is a strength of the study. The large sample size, which provides the basis for the generalizability of the findings, the examination of various and multiple components of women's care needs (personal care, newborn care, physical needs, and emotional needs), and the diversity of sampling based on pregnancy status, which provides the basis for understanding the differences in perceptions of primiparous and multiparous women about various aspects of care, are other strengths of the study. In this study, the researchers used a quantitative approach to examine the quality of postpartum care from the perspective of the women studied, while studies of this kind are better to qualitatively assess the individuals under study. This study was used to state the problem and necessity of the research, and its results will be used in designing interview questions and analyzing and interpreting the results.

7- Simbar et al. (2005) in a descriptive study entitled "Investigating the quality of postpartum care in hospitals affiliated with Shahid Beheshti University of Medical Sciences and Health Services" studied 60 women referring to health centers after natural childbirth. The mean age of the samples was 24.3 years, and the pregnancy and number of live births were one to two in 78% and 83% of the samples, respectively, and the majority of them had no cases of infant mortality or miscarriage. The researchers stated that the quality of care provided in the postpartum ward was poor in the majority of cases, and given the importance of this care in preventing postpartum complications, they recommended conducting further studies and research on the causes of the low quality of this care (12).

The failure to specify the variables studied in order to measure the quality of postpartum care is one of the weaknesses of this study. (The researchers did not mention the items and criteria with which they measured the quality of postpartum care). Another weakness of this study is that the quality of postpartum care was measured through a quantitative study, while determining the dimensions of the quality of care and its challenges requires considering the individual's perspective, experiences, emotions, and feelings, and social interactions, which must be achieved through a qualitative study. This study was used to state the problem and the necessity of the research, and its results will be used in the analysis and interpretation of the results.

8- Ghobadi et al. (2018) in a descriptive-analytical study titled "Investigating the level of satisfaction with the experience of natural childbirth and its related factors in women in Rasht" studied 126 women hospitalized in the postpartum ward. The samples were selected using the convenience method and the data collection tool was a researcher-made questionnaire and included three sections: human factors, environmental factors, and measures related to the labor and delivery process. The research data were analyzed using Spearman, Pearson, and t-test correlation tests. According to the findings reported by the researchers, the average score of mothers' satisfaction with the natural childbirth experience was 59.03, which was moderate. The highest level of satisfaction was reported with environmental factors (68 percent). Mothers' satisfaction with access to labor facilities and receiving sufficient information about the stages of labor and delivery had the same effects on mothers' satisfaction with the childbirth experience. The respectful attitude of labor personnel was the most important factor in attracting mothers' satisfaction with the childbirth experience. In fact, the quality of relationships between women and caregivers is an important determining factor. Advancing prenatal care towards woman-centered obstetric care and establishing appropriate relationships with women are among the most effective measures to improve the quality of obstetric care and improve women's satisfaction with natural childbirth (42).

Investigating the different dimensions involved in women's satisfaction with the natural childbirth experience by separating environmental, human, and childbirth process factors is one of the strengths of this study

**Qualitative Studies**

9- Forster et al. (2008) conducted a qualitative study titled “The Early Postpartum Period: Exploring Women’s Perspectives, Expectations and Experiences of Care Using Focus Groups” in urban and rural Victoria, Australia, and interviewed 52 people in eight focus groups. Participants included eight pregnant women, 42 postpartum women, and two of their partners. The interviews were designed to explore participants’ experiences and expectations of postpartum care in hospital and at home, with an emphasis on length of hospital stay, professional and social support, and continuity of care. General themes extracted from the interviews included anxiety/fear, and the transition to motherhood and parenting. The researchers stated that the needs of first-time mothers were different from those of mothers who had previously experienced motherhood, and that women in this study were generally concerned about the safety of their new baby, and were unsure of their ability to care for their newborn as a new mother. They emphasized in their research results that, based on existing views, the physical presence and availability of professional support helped to reduce these concerns, and this is especially true for women giving birth to their first baby, and care providers should be aware of these issues. It is very important that women's concerns and needs are taken into account when planning the provision of services. If anxiety about new parenthood is the dominant view, care providers should recognize this need and ensure that care is individualized and provided to address the specific concerns of each woman/family (16).

In a criticism of the above study, it can be noted that the researchers aimed to examine people's experiences and expectations of postpartum care, while some of the samples under study were pregnant people. Therefore, given that people who are in the postpartum period can have a better understanding of their expectations in this period, it is recommended that people in the postpartum period be used in such studies. In addition, in order to achieve a more accurate and comprehensive view of fathers' opinions, if the number of male samples is increased, the results of the study will be more reliable. Separating the results of the study from the perspectives of primiparous women and women with previous pregnancy experience is considered one of the strengths of this study. This study has something in common with the present study due to the qualitative nature of the methodology and the participation of fathers as part of the study participants. This study was used to state the problem and necessity of the research, and due to the qualitative approach, this study can be used in the section of entry criteria and its results in designing interview questions and analyzing and interpreting the results.

10- McLeish et al. (2020) conducted their research entitled "A qualitative study of primiparous mothers' experiences of postpartum social support from health professionals in the UK" using a phenomenological method using in-depth and semi-structured interviews with 32 mothers with different backgrounds. Using inductive thematic analysis, they reached four general themes in different dimensions of social support (emotional, evaluative, informational, and practical). In their study, there were nine themes related to social support, with the strongest mapping to the dimensions of appraisal and informational support.

Themes related to the appraisal dimension included praise and validation, criticism and undermining, and creating a sense of helplessness; and themes related to the informational support dimension included preventive measures, education about danger signs and abnormal conditions, and clearing up confusion about postpartum care. The emotional support dimension included feeling understood and heard, impersonal care (lack of attention to individuality in care), and being ignored; and the practical support dimension included empowering sexual partners to provide support. The researchers concluded that health professionals can play an important role in helping primiparous mothers in the postpartum period by providing appropriate and personalized care, building self-confidence, conducting appropriate individual assessments, and providing emotional and informational social support alongside clinical care, and this requires professional training and support to ensure that all health professionals are able to provide social support alongside their other services (43).

Women's support needs in the postpartum period have various and extensive dimensions, and this study only examined the social support dimension, which is considered a weakness of this study. Therefore, this study has something in common with our study by examining one of the dimensions of women's support needs after childbirth. Therefore, in the upcoming study, we will try to obtain more comprehensive results by examining different dimensions of couples' support needs. Also, considering the results mentioned regarding primiparous mothers, a separate study of primiparous and multiparous mothers in the upcoming study can confirm the difference in the support needs of these two groups of women as valuable results for us. The results of this study will be used in designing research questions and analyzing the results.

11- Pollack et al. (2005) conducted a qualitative study titled “Fathers with First-Time Parenting Experience and Postpartum Stressors” on a convenience sample of 19 participants, including fathers who were parenting for the first time between the ages of 18 and 45. Data were collected through telephone interviews. A survey and interviews with fathers were used to measure stressors, and the Everyday Stress Index (ESI) was used to assess the nature and severity of stressors. The results showed that factors such as feeling like they did not have enough time for many responsibilities, financial issues, and concerns about the health of the child and other family members were sources of stress. The researchers reported that, with the exception of minor differences in ratings, everyday stressors among fathers who were parenting for the first time were very similar to stressors previously reported among primiparous mothers. Although the sample size was small and the results may not be generalizable, these findings suggest that educational efforts by health care professionals can be beneficial for fathers and mothers during pregnancy and postpartum (21).

The researchers used accessible samples for the study and limited them to men who had become fathers for the first time. While using a sampling approach with maximum diversity increased the generalizability of the findings, and using the opinions of fathers who had previously fathered children could provide more useful information by comparing their opinions with those of fathers who had their first experience of fathering children. One of the strengths of the study is the comparison of the results by the researchers with the results of similar studies in primiparous mothers, which provides the reader with useful information and a comprehensive perspective. This study is similar to the present study in that it examined fathers, so in examining the support needs of fathers in the postpartum period, the questions used by these researchers in the interview can be used as a model, and the results of their study are compared with the upcoming study in the discussion and conclusion section. This study was used in the research proposal in writing the research necessity and problem statement.

12- Adams et al. in a qualitative study in 2023 titled "Assessing Postpartum Care Needs: Women's Perceptions of Postpartum Care, Barriers and Educational Needs" conducted eight focus group discussions among 54 women in the postpartum period in four health centers in the Sagnarigo Tamale District of Ghana. After transcribing and translating the recorded data and thematic analysis, six main themes were extracted, including: newborn-focused postpartum care, postpartum practices, inadequate awareness of postpartum danger signs, barriers to accessing postpartum care, poor mental health experiences, and the need for postpartum education. According to the researchers, postpartum care for women in this study was primarily considered as caring for the newborn after delivery and obtaining key information about physical and mental health care for the mother, and lack of access to information in these areas can lead to poor compliance with the postpartum period and lack of knowledge about danger signs for common causes. Postpartum morbidity and mortality are critical issues. Therefore, future research needs to understand how to communicate important information about postpartum mental and physical health to better protect mothers (44). This study is aligned with the qualitative part of the present study, with the overall goal of assessing women’s needs and perceptions of postpartum care, and will be compared and analyzed in the design of research questions and in the discussion and conclusion section. The intervention design, implementation, and evaluation sections of the present study are in line with the research proposal of the researchers of this study.

**Review Studies**
13- Lithgow et al. (2021) conducted a systematic review of qualitative evidence in a study titled “How women’s experiences and perceptions of care influence uptake and use of postnatal care: a qualitative systematic review across sub-Saharan Africa” between 2009 and 2019. Their review included all qualitative texts in English that “explored women’s perceptions and experiences of the quality of postnatal care they received (respectful and non-respectful) and how this influenced their decisions to access postnatal care.” Thematic analysis was conducted to extract sub-themes and main themes. Fifteen studies were included with data from interviews with 985 women in eight countries. In these studies, respectful care was defined as providers being kind, supportive, and attentive to women’s needs, and disrespectful care was defined as verbal/physical abuse and power imbalances between women and providers (powerful actions by providers). The study results acknowledged that women prefer to receive health care services where health care providers interact respectfully, and evidence suggested that vulnerable women (adolescents, women from low socioeconomic status, and HIV-positive women) are more likely to receive disrespectful care. This systematic review explains how aspects of respectful and disrespectful maternal care influence women’s perceptions and experiences and decisions to access postpartum care services. There is a need for renewed focus to prioritize respectful maternity care and to sustainably provide good-quality postpartum care for all women and their newborns in a way that meets their expectations and health needs (24).

Despite the inclusion of studies from different countries in this review, which increases the generalizability of the findings as a result of the diversity of the overall sample, the limited number of studies reviewed is one of the criticisms that can be made of the study. Considering the results mentioned regarding vulnerable women and the emphasis on them, which is considered one of the strengths of this study, in order to increase the diversity in sampling and increase the generalizability and breadth of the research findings in the present study, an attempt will be made to include these target groups of women in the study. Therefore, by considering the demographic variables of the studies reviewed in this systematic review, an attempt will be made to maximize diversity in sampling. The results of this study will also play a role in the analysis and interpretation of the results. 14- Warren et al. (2022) conducted a systematic review of qualitative and mixed studies in a study entitled "Fathers' perceptions and experiences of support as a parenting partner during the perinatal period". A systematic search was conducted through various databases. In general, 23 articles were included in the study. According to the results of the study, men were willing to be supported and undergo a rite of passage to adapt to the transition to parenthood. This transitional process helps men to express their commitment to the role of father and prepare to act as a role model for future children. Fathers need comprehensive support at all levels of the ecosystem, including policymaking, socio-cultural changes, as well as changes in the workplace and understanding and support from colleagues, family, peers and especially health care providers. In fact, developing parental involvement requires committed approaches at all levels of micro, meso and macro (23).
Given that the researchers have examined mixed studies in addition to qualitative studies in their review, this can be considered a strength of this study. Although this study examined fathers' perceptions of support, the results did not mention which categories are considered support from the fathers' perspective, which is one of the gaps in the results of the review of the aforementioned studies. The results of this study regarding fathers' perceptions of "support" are in line with the variable under study (support needs) in the upcoming study. Therefore, they will be compared and analyzed in the design of the research questions and in the discussion and conclusion section.

The aforementioned studies add to our understanding of women's experiences and expectations in the early postpartum period and provide information on which improvements can be made in postpartum services and care.

The results of the studies indicated that women were more satisfied with the aspects of midwifery care related to providing practical advice and assistance regarding newborn care and their physical recovery, and in fact, the physical aspects of maternal health and newborn care receive the main focus of postpartum care, while women's support needs in the postpartum period have different and extensive dimensions, and caregivers devote less time and attention to areas related to psychosocial care, providing ongoing counseling, emotional care, and providing information about maternal health needs (such as immunization and contraception).

In addition, the studies conducted in terms of only addressing some aspects of postpartum women's support needs or only involving men or women who have experienced fatherhood for the first time have gaps that we are trying to fill in the upcoming study because we need to examine different aspects of couples' support needs to obtain more comprehensive results. In addition, using the opinions of fathers or mothers who have previously had a history of having children can provide more useful information while comparing the opinions of these people.

Therefore, in the upcoming study, we will try to study the various dimensions involved in couples' satisfaction with the natural childbirth experience, by using maximum diversity in the samples in terms of age, childbearing status, etc., and by including fathers in the study along with their wives (as couples) in order to investigate what categories are considered support from their perspective and have not been addressed in previous studies, as well as by addressing vulnerable groups of women. It should be noted that domestic studies have not addressed the category of supportive care needs in postpartum wards and health centers in a qualitative manner, and the existing studies in this field are quantitative. Given that no qualitative research has been conducted on this issue in Iran so far, and previous studies have only measured the quality of postpartum care quantitatively, this study aims to explain the supportive care needs of couples in the postpartum period with a mixed approach and design and implement a needs-based intervention to improve health services in the postpartum period.

**Definition of the words**

**Need**

**Theoretical definition:** Need refers to a situation in which the existing or present situation is far from the desired situation. The desired situation includes ideals, norms, preferences, expectations, and different perceptions of what should be (45).

Practical definition: In this study, the supportive care needs of couples in the postpartum period are extracted through in-depth semi-structured interviews.

**Supportive care**

**Theoretical definition:** A type of care that addresses various informational, physical, emotional, and psychological aspects of the needs of individuals in unusual and special life circumstances. The primary goal of supportive care is to improve the quality of life of individuals. Key dimensions of supportive care include pain and symptom management, emotional and psychological support, improved communication, decision-making ability, addressing practical needs (financial concerns, access to resources, etc.), respect for dignity and autonomy, and the provision of coordinated and integrated care (5).

**Practical definition:** In this study, supportive care is defined as the care provided to mothers and their partners in various informational, physical, psychological, and emotional aspects during the postpartum period by healthcare staff in inpatient and outpatient settings, and is consistent with the theoretical definition. This concept will be elicited through in-depth, semi-structured interviews.

**Postpartum period:**

**Theoretical definition:**

The postpartum period, also known as the puerperium or fourth trimester, refers to the first few weeks after birth. When physiological and anatomical changes associated with pregnancy return to the non-pregnant state (46).

**Practical definition**: In this study, the practical definition is consistent with the theoretical definition and includes the sampling period of 8 weeks after delivery.

**Research questions and hypotheses**

**Research hypotheses or questions (according to the project objectives):**

**Research questions:**

1- What is the explanation of Iranian couples about the need for supportive care in the postpartum period?

2- What is the explanation of health service providers about the need for supportive care in the postpartum period?

3- What is the explanation of experts (key informants, managers and policymakers) about the need for supportive care in the postpartum period?

4- What are the most important supportive care needs of couples in the postpartum period according to the expert panel?

5- Based on the literature review and the expert panel, what intervention is appropriate in the field of supportive care needs of couples in the postpartum period?

6- What are the facilitators and barriers to supportive care of couples in the postpartum period?

7- Is the designed intervention effective in meeting the care needs of couples?

**Research hypotheses:**

Implementation of the intervention is effective in meeting the needs of couples regarding support and postpartum care.

**references**

1. Infurna MR, Bevacqua E, Costanzo G, Falgares G, Giannone F. Psychosocial Risk Factors and Psychopathological Outcomes: Preliminary Findings in Italian Pregnant Women. Women. 2023;3(1):121-31.
2. Austin M-P, Committee MSPSA. Marcé International Society position statement on psychosocial assessment and depression screening in perinatal women. Best Practice & Research Clinical Obstetrics & Gynaecology. 2014;28(1):179-87.
3. Asadi M, Noroozi M, Alavi M. Identifying women’s needs to adjust to postpartum changes: a qualitative study in Iran. BMC Pregnancy and Childbirth. 2022;22(1):115.
4. Slomian J, Emonts P, Vigneron L, Acconcia A, Glowacz F, Reginster J-Y, et al. Identifying maternal needs following childbirth: A qualitative study among mothers, fathers and professionals. BMC pregnancy and childbirth. 2017;17(1):1-13.
5. Chambers EJ, Brown E, Germain M. Supportive care for the renal patient: OUP Oxford; 2010.
6. El-Khoury F, Sutter-Dallay A-L, Panico L, Charles M-A, Azria E, Van der Waerden J, et al. Women’s mental health in the perinatal period according to migrant status: the French representative ELFE birth cohort. The European Journal of Public Health. 2018;28(3):458-63.
7. Simsek A, Balkan E, Caliskan E. Determination of mothers' thoughts and adaptation behaviors regarding the infant: A descriptive study. Pediatrics & Neonatology. 2022;63(3):276-82.
8. McKellar LV, Pincombe JI, Henderson AM. Insights from Australian parents into educational experiences in the early postnatal period. Midwifery. 2006;22(4):356-64.
9. Rudman A, El-Khouri B, Waldenström U. Evaluating multi-dimensional aspects of postnatal hospital care. Midwifery. 2008;24(4):425-41.
10. Health WHOR. Medical eligibility criteria for contraceptive use: World Health Organization; 2010.
11. سیمبر م, غفاری ف, ترک‌زهرانی ش, علوی‌مجد ح. کیفیت ارایه مراقبت های مامایی به زنان بستری در بخش زایمان بیمارستان های منتخب، دانشگاه علوم پزشکی کردستان. فصلنامه پایش. 1388:11.
12. سیمبر م, علی‌زاده‌دیبازری ز, عابدسعیدی ژ, علوی‌مجد ح. بررسی کیفیت مراقبت های پس از زایمان در بیمارستان های تابعه دانشگاه علوم پزشکی و خدمات بهداشتی و درمانی شهید بهشتی در سال 1382. نشریه پژوهنده. 1384;10(1):9-15.
13. علیدوستی م, طهماسبی م, رئیسی م. بررسی رضایت مندی زنان پس از زایمان از خدمات و امکانات زایشگاه بیمارستان هاجر شهرکرد. مجله بالینی پرستاری و مامایی. 1392;2(1):1-8.
14. Brown SJ, Davey M-A, Bruinsma FJ. Women's views and experiences of postnatal hospital care in the Victorian Survey of Recent Mothers 2000. Midwifery. 2005;21(2):109-26.
15. Forster D, McLachlan H, Yelland J, Rayner J, Lumley JP, Pin C. A review of in-hospital postnatal care in Victoria. Final report Melbourne: La Trobe University. 2005.
16. Forster DA, McLachlan HL, Rayner J, Yelland J, Gold L, Rayner S. The early postnatal period: exploring women's views, expectations and experiences of care using focus groups in Victoria, Australia. BMC pregnancy and childbirth. 2008;8(1):1-11.
17. Arefadib N, Shafiei T, Cooklin A. Barriers and facilitators to supporting women with postnatal depression and anxiety: A qualitative study of maternal and child health nurses’ experiences. Journal of Clinical Nursing. 2023;32(3-4):397-408.
18. Sachdeva J, Yang SN, Gopalan P, Worley LL, Mittal L, Shirvani N, et al. Trauma Informed Care in the Obstetric Setting and Role of the Perinatal Psychiatrist: A Comprehensive Review of the Literature. Journal of the Academy of Consultation-Liaison Psychiatry. 2022;63(5):485-96.
19. Finlayson K, Crossland N, Bonet M, Downe S. What matters to women in the postnatal period: A meta-synthesis of qualitative studies. PloS one. 2020;15(4):e0231415.
20. Geuens S, Polona Mivšek A, Gianotten W. Midwifery and Sexuality: Springer Nature; 2023.
21. Pollock MA, Amankwaa LC, Amankwaa AA. First-time fathers and stressors in the postpartum period. The Journal of perinatal education. 2005;14(2):19-25.
22. Swanson V, Hannula L. Parenting stress in the early years–a survey of the impact of breastfeeding and social support for women in Finland and the UK. BMC Pregnancy and Childbirth. 2022;22(1):699.
23. Leahy‐Warren P, Philpott L, Elmir R, Schmied V. Fathers’ perceptions and experiences of support to be a parenting partner during the perinatal period: A scoping review. Journal of Clinical Nursing. 2022.
24. Lythgoe C, Lowe K, McCauley M, McCauley H. How women's experiences and perceptions of care influence uptake of postnatal care across sub-Saharan Africa: a qualitative systematic review. BMC Pregnancy and Childbirth. 2021;21(1):1-11.
25. Hui D, De La Cruz M, Mori M, Parsons HA, Kwon JH, Torres-Vigil I, et al. Concepts and definitions for “supportive care,”“best supportive care,”“palliative care,” and “hospice care” in the published literature, dictionaries, and textbooks. Supportive Care in Cancer. 2013;21:659-85.
26. Fisher C, Hauck Y, Fenwick J. How social context impacts on women's fears of childbirth: a Western Australian example. Social science & medicine. 2006;63(1):64-75.
27. Reibel T. Normal birth: a thing of the past or the new future for primary health care’. Primary Health Care Research & Development. 2004;5(4):329-37.
28. Reed R, Sharman R, Inglis C. Women’s descriptions of childbirth trauma relating to care provider actions and interactions. BMC pregnancy and childbirth. 2017;17:1-10.
29. Albanese AM, Geller PA, Sikes CA, Barkin JL. The importance of patient-centered research in the promotion of postpartum mental health. Frontiers in Psychiatry. 2021;12:720106.
30. van Teijlingen ER, Hundley V, Rennie AM, Graham W, Fitzmaurice A. Maternity satisfaction studies and their limitations:“What is, must still be best”. Birth. 2003;30(2):75-82.
31. Continuity of Care. Australia: Victorian Department of Human Services; 2004.
32. Perrenoud P, Chautems C, Kaech C. “Whatsapping” the continuity of postpartum care in Switzerland: A socio-anthropological study. Women and birth. 2022;35(3):e263-e74.
33. Van Teijlingen E. Maternity home care assistants in the Netherlands. Midwifery and the Medicalization of Childbirth: Comparative Perspectives; van Teijlingen, E, Lowis, G, McCaffery, P, Porter, M, Eds. 2004:163-72.
34. Delivery FC. Improving Maternity Services in England and Wales. Audit Commission. 1997.
35. Lefèvre M, Van den Heede K, Camberlin C, Bouckaert N, Beguin C, Devos C, et al. Impact of shortened length of stay for delivery on the required bed capacity in maternity services: results from forecast analysis on administrative data. BMC Health Services Research. 2019;19(1):1-9.
36. Derakhshanpour F, Kashani L, Taghavi S, Salimi Z, Shahini N. Effect of a Psycho education Interventions on Postpartum Health in North of IRAN. Journal of Clinical and Basic Research (JCBR). 2020;4(4).
37. Chen C-H, Tseng Y-F, Chou F-H, Wang S-Y. Effects of support group intervention in postnatally distressed women: A controlled study in Taiwan. Journal of psychosomatic research. 2000;49(6):395-9.
38. Glavin K, Smith L, Sørum R, Ellefsen B. Supportive counselling by public health nurses for women with postpartum depression. Journal of advanced nursing. 2010;66(6):1317-27.
39. Koçak V, Ege E, İyisoy MS. The development of the postpartum mobile support application and the effect of the application on mothers' anxiety and depression symptoms. Archives of psychiatric nursing. 2021;35(5):441-9.
40. Baghersad Z, Mokhtari F, Bahadoran P. Effect of home care on husband’s support during the postpartum period. Journal of Holistic Nursing And Midwifery. 2019;29(4):210-7.
41. Fenwick J, Butt J, Dhaliwal S, Hauck Y, Schmied V. Western Australian women's perceptions of the style and quality of midwifery postnatal care in hospital and at home. Women and birth. 2010;23(1):10-21.
42. Ghobadi M, Ziaee T, Mirhaghjo N, Pazandeh F. Evaluation of satisfaction with natural delivery experience and its related factors in Rasht women. Journal of Health and Care. 2018;20(3):215-24.
43. McLeish J, Harvey M, Redshaw M, Alderdice F. A qualitative study of first time mothers’ experiences of postnatal social support from health professionals in England. Women and Birth. 2021;34(5):e451-e60.
44. Adams YJ, Miller ML, Agbenyo JS, Ehla EE, Clinton GA. Postpartum care needs assessment: women’s understanding of postpartum care, practices, barriers, and educational needs. BMC Pregnancy and Childbirth. 2023;23(1):502.
45. Fathi Vajargah K. Educational need assessment: models and techniques. Tehran: Abeeze publication. 2005.
46. Berens P. Overview of the postpartum period: Normal physiology and routine maternal care. UptoDate. 2020;15:1-34.
47. Mengshoel AM. Mixed methods research–so far easier said than done? Manual Therapy. 2012;17(4):373-5.
48. حسنی م. در آمدی به روش شناسی تلفیقی در پژوهش های میان رشته ای علوم اجتماعی. فصلنامه مطالعات میان‌رشته‌ای در علوم انسانی. 1389;2(4):137-53.
49. Creswell JW, Creswell JD. Research design: Qualitative, quantitative, and mixed methods approaches: Sage publications; 2017.
50. Teddlie C, Tashakkori A. Foundations of mixed methods research: Integrating quantitative and qualitative approaches in the social and behavioral sciences: Sage; 2009.
51. Creswell JW, Clark VLP. Designing and conducting mixed methods research: Sage publications; 2017.
52. ضیایی م, زندی ا, عباس‌پور ن, عبدی م. توسعه‌ی پایدار گردشگری از دیدگاه دو مکتب ایده‌آلیسم و پراگماتیسم. برنامه ریزی و توسعه گردشگری. 1393;3(8):11-30.
53. Elo S, Kyngäs H. The qualitative content analysis process. Journal of advanced nursing. 2008;62(1):107-15.
54. Lauri S, Kyngas H. Developing nursing theories. Vantaa, Finland: Werner Söderström, Dark Oy. 2005.
55. Hsieh H-F, Shannon SE. Three approaches to qualitative content analysis. Qualitative health research. 2005;15(9):1277-88.
56. Graneheim UH, Lindgren B-M, Lundman B. Methodological challenges in qualitative content analysis: A discussion paper. Nurse education today. 2017;56:29-34.
57. Wildemuth BM. Applications of social research methods to questions in information and library science: Abc-Clio; 2016.
58. MacPhail A. Nominal group technique: a useful method for working with young people. British Educational Research Journal. 2001;27(2):161-70.
59. Manera K, Hanson CS, Gutman T, Tong A. Consensus methods: nominal group technique. 2019.
60. Speziale HS, Streubert HJ, Carpenter DR. Qualitative research in nursing: Advancing the humanistic imperative: Lippincott Williams & Wilkins; 2011.
61. Polit D, Beck C. Essentials of Nursing Research 6th edition. Philadephia MA. Lippincott; 2006.

Supervisors and Advisors

| **Name** | **Family** | **Name EN** | **Family EN** |  |  |  | **پست الکترونیک** |
| --- | --- | --- | --- | --- | --- | --- | --- |

| zahra | behboodi moghadam | zahra | behboodi moghadam | Second Advisor | School of Nursing and Midwifery | Reproductive health | behboodi@tums.ac.ir |
| --- | --- | --- | --- | --- | --- | --- | --- |
| Shirin | Shahbazi seyghaldeh | Shirin | Shahbazi seyghaldeh | First Advisor | School of Nursing and Midwifery | Reproductive health | shahbazishirin@yahoo.com |
| Elham | Ebrahimi | Elham | Ebrahimi | First Supervisor | School of Nursing and Midwifery | Reproductive health | ebrahimi_308@yahoo.com |

Methods of study

**Method of implementation**

**Type of study and implementation method**

**Specify the type of study and explain the items opposite the paragraph in the implementation method section.**

The present study is a multi-stage mixed study. The reason for choosing a mixed approach (collecting both qualitative and quantitative data) by the researcher was that mixed research methods are based on a pragmatic philosophical approach. The foundation of pragmatic philosophy emphasizes the consequences of research, and from its point of view, paying attention to the question asked in the research is more important than paying attention to the research method. Hence, mixed research methodology is the simultaneous explanation and understanding of the complexities, dimensions, structure, forms of phenomena and social realities, and the use of various designs, methods, theories and data sources, in other words, the use of a combination of quantitative and qualitative research methods, is an ontological and epistemological necessity; because no research method alone is complete and cannot answer all aspects of the research question.

At a practical level, mixed methods provide a sophisticated approach to research that is appealing to those at the forefront of new research practices. It can also be an ideal approach if the researcher has access to both quantitative and qualitative data. Since mixed methods research is relatively new as a distinct research approach in the social sciences and humanities, it is useful to provide a basic definition and description of the mixed method approach in the research methodology (49). The present study is also a type of multi-stage mixed method study that Cresswell and Clark (2017) have identified as one of the five main mixed method research designs (51). The study will have three qualitative components – an intervention design and a quantitative component.

The first stage of the qualitative part of the study

First, a sequential exploratory qualitative study will be conducted with a contractual content analysis approach with the aim of explaining the supportive care needs of couples in the postpartum period.

Research setting

The research setting of qualitative research is the real field. That is, it will be carried out in the place where the people in question live and their experiences take place or in places chosen by the participants. The setting of this research and the place of discussion and interview will be, according to the participants' opinions and coordination with other members of the group, mainly in hospitals and health centers affiliated with Kermanshah University of Medical Sciences.

Research community and participants:

Since qualitative research seeks to examine the meaning of reality, participants are people who are a rich source of information about the phenomenon under study. The study population in this study will be women receiving postpartum care and their spouses in hospitals and health centers affiliated with Kermanshah University of Medical Sciences who have experienced at least one delivery (natural or cesarean section) and postpartum care, as well as reproductive health service providers including midwives working in inpatient wards, gynecologists, gynecological residents, and nurses who have at least two years of experience providing services to the aforementioned population, as well as managers and policymakers in the field of reproductive health and women's health, psychologists, and midwives working abroad. They will be selected with rational and purposeful sampling with maximum diversity, and sampling and interviews will continue until data saturation.

Sampling method:

Individuals are selected for interviews in a purposeful manner with maximum diversity. Purposeful sampling means that the researcher seeks to select participants who have experiences in the field of the phenomenon under study with key concepts to be explored. To achieve maximum diversity, the current study attempts to include participants with different ages, educational levels, socioeconomic status, and variations in pregnancy status and type of delivery, gender of the newborn, whether the pregnancy was wanted or unwanted, the time elapsed since delivery, the individual's support status, etc., and in the reproductive health service provider section, individuals with different employment histories are used. Women who have given birth (either by natural birth or cesarean section) at the sampling site (hospitals and health centers affiliated with Kermanshah University of Medical Sciences) and their spouses who are physically, mentally, and communicatively fit for the interview, consent to participate in the study, and a maximum of eight weeks have passed since the birth of their child will be included in the study. In the reproductive health service providers section, health care personnel including midwives working in the aforementioned departments, gynecologists, gynecological residents, and nurses who have at least two years of experience providing services to the aforementioned population will be included in the study if they agree to participate in the study. The next group to be studied will be managers and policymakers in the field of reproductive health services and women's health, psychologists, and midwives working abroad. After explaining the objectives of the study, while emphasizing the confidentiality of the information, if they wish to participate in the study, coordination will be made in terms of time and place for the interview.

Acceptable criteria for entry into the study

Individuals participating in this study will have the following characteristics:

• Have a minimum level of literacy

• Be in a suitable physical, mental, and communication condition to conduct an interview

• Be able to communicate and conduct an interview.

• Be Iranian and able to understand and speak Persian.

• Have at least one history of childbirth and postpartum care.

• Being in the postpartum period (up to 8 weeks after natural birth or cesarean section)

• Being hospitalized in the postpartum wards of one of the hospitals affiliated with Kermanshah University of Medical Sciences or visiting outpatient health centers to receive postpartum care

• Reproductive health service providers with at least two years of work experience

Exclusion criteria

• Suffering from known mental illnesses and mood disorders

• Death of a fetus or newborn during a recent delivery process

• Suffering from a known abnormality in the newborn

• An unfortunate incident in the past 6 months in the life of the individuals

Sample size calculation method:

• In qualitative studies, the researcher continues sampling until data saturation is reached and until no new data is added. Therefore, in this study, sampling continues gradually until data saturation.

Data collection method:

Data collection method and qualitative data collection tools

Individual interview

In-depth and semi-structured interviews, the response rate of people is high and they have greater freedom to express their views and express their opinions. In this type of interview, the researcher prepares a general guide or checklist and based on it, identifies general axes in advance and has questions in mind for each axis during the interview. Initially, all respondents are asked the same questions, but they are free to present their answers in any way they wish, that is, while the general structure of the interview is the same for all participants, the interviewer can ask other questions when necessary. In this case, the researcher is responsible for coding the answers and classifying them. In this type of interview, the researcher is looking for specific information so that he can compare and contrast them with information obtained from other interviews.
In the present study, this approach will also be used to explain the supportive care needs of couples in the postpartum period. Participants will be selected purposively. An attempt will be made to interview women with one or more previous births and their spouses, as well as reproductive health service providers in different fields and job categories (midwives, nurses, physicians, officials and managers) with multiple backgrounds, with maximum frequency. Individual interviews will also be conducted with key informants (reproductive health managers and policymakers, women's health policymakers, psychologists, midwives working abroad). After the final approval of the research proposal by the Graduate Education Council of the relevant specialized university and obtaining scientific and ethical approval from Tehran University of Medical Sciences, written consent will be obtained from the Vice Chancellor for Education and the Vice Chancellor for Treatment to begin conducting the research and collecting data. Sampling will begin with the researcher visiting health centers and hospitals affiliated with Kermanshah University of Medical Sciences. After contacting the participants, the researcher will introduce himself/herself and explain the objectives of the study, and the time and place of the interview will be coordinated with them. For the interview, a friendly relationship will be established with the participants and they will be assured of the confidentiality of the information. After explaining the purpose of the study, consent will be obtained from the participants in the research. The interviews will begin with a few open-ended questions. Then, based on the initial answers and the interview guide, the needs for supportive care in the postpartum period will be asked, as well as suggestions for meeting these needs. Also, as needed, probing questions such as "What do you mean?" or "If you can, please explain more" will be used in the interviews. According to the interview guide, simple and more general questions are asked at first, and according to the way the participants respond and their experiences, the interview continues with more detailed questions. The interview questions are flexible and new questions will be added to the interview guide if needed. In the present study, during the interviews, observations and notes will be taken in the field to examine how care is provided and the interactions of service providers with women who have given birth and their spouses. Attention will be paid to all emotional and behavioral movements and reactions of the interviewee and the environment. After completion, the recorded interviews and notes taken will be transcribed and analyzed.

Questions related to couples in the postpartum period

1- Explain your experience and feelings about the care provided to you after childbirth?

3- What do you think should have been paid attention to in this care?

4- What expectations did you have from the doctor, nurse, and midwives?

5- What needs have you had during this period and do you think there was no specific service for them or do you think it is necessary to provide services to couples and families in these cases?

6- What problems did you face in the postpartum period regarding yourself, your spouse, and the baby?

7-How did you resolve these problems?

8-Did you refer to health and medical service providers to resolve your problems and needs? If yes, please share your opinion and experience of receiving these services.

9-What were your information needs during this period and from what sources did you resolve your needs? Were these resources of quality and did they meet your needs well and completely? Did they have any shortcomings? Please explain.

10-What were your support and emotional needs during this period and from what sources did you resolve your needs? Were these resources of quality and did they meet your needs well and completely? Did they have any shortcomings? Please explain.

11-What were your physical needs and physical care during this period and from what sources did you resolve your needs? Were these resources of quality and did they meet your needs well and completely? Did they have any shortcomings? Please explain.

12-How did you see your own abilities? What problems did you feel in yourself?

13-What are your current concerns?

And........

Questions for reproductive health providers:
1-What services do you provide to women in the postpartum period? Describe a typical day of your work in providing postpartum care.

2-What are the physical, informational, and emotional support and care in the postpartum period, and do you think this support and care is complete and well-provided, or is it lacking? Please explain.

3- In your opinion, what are the issues and concerns related to couples' health in the postpartum period and the reasons for them? Explain.

4-What physical and psychological health needs of couples in the postpartum period are met in this center?

5- In your opinion, what needs do couples have in the postpartum period that are not met in hospital centers?

6- Explain about couples' access to supportive services and care in the postpartum period?

7- Which supportive care needs do couples most desire to have met?

8- As a reproductive health service provider, what obstacles do you see in providing your services in line with the supportive care needs of these women?

9- As someone who is familiar with the problems of couples in the postpartum period, what needs do you know in the field of postpartum support that have not been answered?

Questions related to psychologists:

1- What is your experience with mental disorders that women and their spouses refer to you in the postpartum period?

2- In what situations and circumstances do couples usually refer to you in the postpartum period? What are their problems in this period?

3- If couples develop psychological disorders in the postpartum period, what do you think is the cause of these problems?

4- How useful can care in hospitals and health centers and care provided by spouses and other members of the support system be in reducing and preventing these problems?

Questions for policymakers:

1- Do you know what services are provided for the postpartum period in Iran? Please explain.

2- Do you know what the difference is between these services and other developed countries and what are the shortcomings and deficiencies?

3- Do you think it is possible to address these shortcomings and deficiencies? And how?

4- Please explain whether some services and care are not available or not deficient and if they were, what difference would it make in economic terms. Do developed countries that have better services also pay attention to economic efficiency? How?

5- What guidelines for postpartum care are provided and what are being developed? Explain how these guidelines are monitored, evaluated and updated?

The rest of the questions related to the policymakers section will be designed after the interview with the couples and based on the needs explained by them.

After the interview questions are finished, the participant will be asked to state if there are any points or comments left. At the end, the possibility of further interviews will be discussed with him/her.

The interviews will be recorded and then transcribed verbatim, with the participant’s permission and assurance that all interviews will be confidential. To check their accuracy, the transcript of the interview will be given to the participant to read and approve.
Data Analysis Method

In the early definitions, content analysis is described as a method for analyzing writings, texts, verbal and non-verbal communication (53). This analysis can be applied to both quantitative and qualitative data. In addition, this analysis can be done in a deductive or inductive manner. The choice of each depends on the purpose of the study. If the existing knowledge on the subject under study is scarce and scattered, it is recommended that content analysis be done in an inductive manner (54).

This method is classified into three categories, including conventional, directional and dense. In the present study, we will use the conventional (conventional) content analysis method. In this method, data are obtained directly from the participants in the study and the categories are not predetermined, but are derived from the text of the data. Therefore, data analysis begins with repeated reading of all the data so that the researcher can immerse himself in the data and gain an overview. The data is then read word for word to highlight words from the text that contain key concepts, thus extracting codes. The researcher then makes initial interpretations of the text and performs an initial analysis. As this process continues, labels for the codes emerge that reflect more than one key concept, usually taken directly from the text and later converted into an initial coding map. The codes are then placed into categories based on how the different codes are related to each other. These categories are used to organize and group the codes into meaningful clusters. Ideally, the number of categories is between 10-15, to be broad enough to accommodate a large number of codes. A larger number of categories can be combined into a smaller number of subcategories based on their relationships. A tree diagram can help organize these categories into a hierarchical structure. Definitions are then created for each category, subcategory, and code. When reporting findings, examples of each code and data category are provided (55). In this study, after each interview, the interview transcript will be transcribed as soon as possible, preferably on the day of the interview. After each interview, the supervising professors and advisors will re-read the interview transcript. The resulting codes will be managed using MAXQDA 12 software.
The conventional content analysis in this study will be analyzed in eight stages based on the method proposed by Jank and Wilmos (2016):

1- Preparing data for qualitative content analysis

2- Deciding on the unit of analysis

3- Classification

4- Testing coding in a sample of the text

5- Extending the coding testing process to the entire text

6- Accessing coding stability

7- Drawing conclusions from the classified or coded data

8- Reporting stage

The next stage is testing the codes and their stability in the text, which will be confirmed by reviewing the codes and agreeing on these codes by the majority of the research team members. To check the stability of the coding, two experienced experts outside the research team will control the codes given to the categories and subcategories. The next stage will include drawing conclusions about the correct classification of the data and the given codes, and the characteristics and relationships in each category and between categories will be examined and analyzed. Each category and subcategory will also be analyzed with the interview text, and finally the categories formed will be interpreted and reported (56, 57).

Second stage: Intervention design section

In this stage of the research, the intervention design to explain the supportive care needs of Iranian couples in the postpartum period will be carried out in two parts:

Part one: Panel of experts (formation of a nominal group) in order to prioritize the needs explained in the qualitative part of the study

In order to design the intervention and establish a connection between the qualitative and quantitative parts of the study, the issues and supportive care needs of couples will be prioritized in a meeting with the presence of reproductive health specialists, reproductive health policy makers, supervisors, and doctors in a nominal group method.

Based on the identified supportive care needs in the first phase, the most important needs identified in this area are prioritized in a meeting consisting of the research team and a group of health professionals and planners (including policymakers, reproductive health providers, supervisors and consultants, etc.). The Nominal Group Technique (NGT) is a method used to generate ideas and reach consensus. This technique was developed in the late 1960s by André Delbeek and André van de Ven and has since been used in various settings, from business to education and government. In fact, the Nominal Group Technique (NGT) is a structured brainstorming process that encourages all group members to share their ideas equally. This decision-making technique can generate ideas, solve problems or make decisions. The basic structure of NGT includes four stages: brainstorming, voting and reducing ideas, discussing and prioritizing ideas, and building consensus. Participants are encouraged to generate as many ideas as possible without judgment or discussion in the first step. In the second step, participants work together to reduce the list of ideas to a manageable number. In the third step, each participant ranks the remaining ideas in order of importance. Finally, in the fourth step, the group works together to reach consensus on the most important ideas. NGT is an effective tool for generating new ideas and reaching agreement within a group (58). A facilitator asks participants to identify ideas individually and contribute to the list in response to a specific question, thus preventing participants from dominating the discussion and allowing everyone in the group to express their opinions equally (59). In this study, at the beginning of the session, after an introduction to the purpose of the session, the results extracted from the qualitative part of the study are presented, and then the invitees are asked to privately record their views on the supportive care needs of couples in the postpartum period. Then, a list of ideas from each group member will be written in a table and shared. Finally, a vote will be taken on the ideas and the most common needs will be identified.

The second part: Review of the literature and development of an appropriate intervention

In this stage, in order to design the intervention, first, considering the main priority determined in the previous stage, interventions and programs related to meeting the supportive care needs of couples in the postpartum period in Iran and other countries will be reviewed. In fact, a literature review will be conducted on the most priority needs of couples in the postpartum period. Accordingly, a search will be conducted in the databases SID; Magiran; Iran Medex; ProQuest; Pubmed; Google Scholar Embase; and web of science and dedicated sites WHO; UNAIDS; IPPF; UNFPAl between the years 2000 and 2023. The interventions and related programs will then be reviewed in a nominal group (NGT) session, consisting of the research team and a group of interested experts, and then the most appropriate intervention will be selected.
Type of intervention:

The proposed intervention is in line with the most important root factors that are associated with women's dissatisfaction with postpartum care and are considered as the most important barriers to achieving optimal health in the field of perinatal and postnatal services, such as the technocratic (biomedical) model governing the country's healthcare system and the medicalization of the birth process, limited staff numbers, inadequate information for staff, not allowing mothers to hug and touch their newborns immediately after delivery, inflexible length of stay, availability of professional support, as well as support challenges such as the lack of counseling facilities on topics such as adapting to changes in their intimate and family relationships, resuming sexual relations, coping skills with parenting, and coping with experienced birth trauma, and addressing concerns such as damage to the reproductive system, body image, and other barriers to providing woman-centered care.

As mentioned, in order to design and validate the intervention, the issues and supportive care needs of couples explained in the qualitative part of the study will be prioritized in a nominal group meeting with the presence of reproductive health specialists, reproductive health policy makers, supervisors, and doctors. Then, considering the main priority determined in the previous stage, interventions and programs related to meeting the supportive care needs of couples in the postpartum period in Iran and other countries will be reviewed and reviewed. Then, the most appropriate intervention (which will be in the form of a clinical trial) will be selected in the nominal group meeting. Validation and evaluation of the intervention will be carried out using the Tider tool. After selecting the intervention and implementing it, existing standard questionnaires in the field of supportive care satisfaction will be used for evaluation according to the variables selected in the quantitative part of the study.

Stage Three: Quantitative Part of the Study (Intervention Test)

The third stage of this research will be a quantitative study. This stage will be conducted with the aim of determining the impact of the designed intervention on the supportive care needs of Iranian couples in the postpartum period. It is worth noting that the type of quantitative study and other related details will be determined in this stage based on the type of intervention selected after prioritizing needs and reviewing the literature. After conducting the intervention study, the quantitative data will be analyzed using SPSS software.

**Study location**

Outside the university

Data Collection Method:

Data Collection Method and Qualitative Data Collection Tool

Individual Interview

In-depth and semi-structured interviews, the response rate of individuals is high and they have greater freedom to express their views and express their opinions. In this type of interview, the researcher prepares a general guide or checklist and based on it, identifies general axes in advance and has questions in mind for each axis during the interview. Initially, all respondents are asked the same questions, but they are free to present their answers in any way they wish, that is, while the general structure of the interview is the same for all participants, the interviewer can ask other questions when necessary. In this case, the researcher is responsible for coding the answers and classifying them. In this type of interview, the researcher is looking for specific information so that he can compare and contrast them with information obtained from other interviews.

In the present study, this approach will also be used to explain the supportive care needs of couples in the postpartum period. Participants will be selected purposively. An attempt will be made to interview women with one or more previous births and their spouses, as well as reproductive health service providers in different fields and job categories (midwives, nurses, physicians, officials and managers) with multiple backgrounds, with maximum frequency. Individual interviews will also be conducted with key informants (reproductive health managers and policymakers, women's health policymakers, psychologists, midwives working abroad). After the final approval of the research proposal by the Graduate Education Council of the relevant specialized university and obtaining scientific and ethical approval from Tehran University of Medical Sciences, written consent will be obtained from the Vice Chancellor for Education and the Vice Chancellor for Treatment to begin conducting the research and collecting data. Sampling will begin with the researcher visiting health centers and hospitals affiliated with Kermanshah University of Medical Sciences. After contacting the participants, the researcher will introduce himself/herself and explain the objectives of the study, and the time and place of the interview will be coordinated with them. For the interview, a friendly relationship will be established with the participants and they will be assured of the confidentiality of the information. After explaining the purpose of the study, consent will be obtained from the participants in the research. The interviews will begin with a few open-ended questions. Then, based on the initial answers and the interview guide, the needs for supportive care in the postpartum period will be asked, as well as suggestions for meeting these needs. Also, as needed, probing questions such as "What do you mean?" or "If you can, please explain more" will be used in the interviews. According to the interview guide, simple and more general questions are asked at first, and according to the way the participants respond and their experiences, the interview continues with more detailed questions. The interview questions are flexible and new questions will be added to the interview guide if needed. In the present study, during the interviews, observation and note-taking will be conducted in the field to examine the care provided and interactions of service providers with postpartum women and their spouses. Attention will be paid to all emotional and behavioral movements and reactions of the interviewee and the environment. After the interview, the recorded interviews and notes taken will be transcribed and analyzed.
Questions related to couples in the postpartum period

1- Describe your experience and feelings about the care provided to you after childbirth?

3- What do you think should have been considered in this care?

4- What expectations did you have from the doctor, nurse, and midwives?

5- What needs did you have during this period and do you think there was no specific service for them or do you think services should be provided to couples and families in these cases?

6- What problems did you face in the postpartum period for yourself, your spouse, and the baby?

7- How did you solve these problems?

8- Did you refer to health and medical service providers to solve your problems and needs? If yes, please share your opinion and experience of receiving these services.

9- What were your information needs during this period and from what sources did you solve your needs? Were these resources of quality and did they solve your needs well and completely? Did they have any shortcomings? Please explain.

10-What were your support and emotional needs during this period and from what sources did you meet your needs? Were these resources of quality and did they meet your needs well and completely? Were there any shortcomings? Please explain.

11-What were your physical needs and physical care during this period and from what sources did you meet your needs? Were these resources of quality and did they meet your needs well and completely? Were there any shortcomings? Please explain.

12-How did you see your own abilities? What problems did you feel in yourself?

13-What are your current concerns?

And........
Questions related to reproductive health service providers:

1- What services do you provide to women in the postpartum period? Describe a typical day of your work in providing postpartum care.

2- What are the physical, informational, and emotional support and care in the postpartum period? In your opinion, is this support and care complete and well-provided or is it lacking? Please explain.

3- What are the issues and concerns related to couples' health in the postpartum period and the reasons for them? Explain.

4- What physical and psychological health needs of couples in the postpartum period are met in this center?

5- What needs do couples have in the postpartum period that are not met in hospital centers?

6- Explain about couples' access to supportive services and care in the postpartum period?

7- Which supportive care needs do couples have the most desire to have met?

8- As a reproductive health service provider, what obstacles do you see in providing your services in line with the supportive care needs of these women?

9- As someone who is familiar with the problems of couples in the postpartum period, what needs do you know in the field of postpartum support that have not been answered?

Questions related to psychologists:

1- What is your experience with mental disorders that women and their spouses refer to you in the postpartum period?

2- In what situations and circumstances do couples usually refer to you in the postpartum period? What are their problems in this period?

3- If couples develop psychological disorders in the postpartum period, what do you think is the cause of these problems?

4- How useful can care in hospitals and health centers and care provided by spouses and other members of the support system be in reducing and preventing these problems?

Questions for policymakers:

1- Do you know what services are provided for the postpartum period in Iran? Please explain.

2- Do you know what the difference is between these services and other developed countries and what are the shortcomings and deficiencies?

3- Do you think it is possible to address these shortcomings and deficiencies? And how?

4- Please explain whether some services and care are not available or not deficient and if they were, what difference would it make in economic terms. Do developed countries that have better services also pay attention to economic efficiency? How?

5- What guidelines for postpartum care are provided and what are being developed? Explain how these guidelines are monitored, evaluated and updated?

The rest of the questions related to the policymaker’s section will be designed after the interview with the couples and based on the needs explained by them.

After the interview questions are finished, the participant will be asked to state if there are any points or comments left. At the end, the possibility of further interviews will be discussed with him/her.

The interviews will be recorded and then transcribed verbatim, with the participant’s permission and assurance that all interviews will be confidential. To check their accuracy, the transcript of the interview will be given to the participant to read and approve.

**Method of calculating sample size and its number**

Method of calculating sample size:

• In qualitative studies, the researcher continues sampling until data saturation is reached and until no new data is added. Therefore, in this study, sampling continues gradually until data saturation.

**Implementation limitations of the design and methods for reducing them**

The individual characteristics and intellectual preoccupations of the research participants can affect the way they respond, which is beyond the researcher's control.

During the interview with the research participants, it is possible that people may not express their true opinions and may state things to attract the interviewer's attention, which, given the characteristics of the type of interview, will have minimal impact on the presentation of the material.

The degree of trust respondents have in the interview is not the same, which is beyond the researcher's control.

Table of Variables

| **Variable Title** | **Variable Role** | **Variable Type** | **Scientific/Practical definition** | **How to measure** | **Scale** |
| --- | --- | --- | --- | --- | --- |

| Age of the woman | Independent | Quantitative/Continuous |  | Demographic questionnaire | Year |
| --- | --- | --- | --- | --- | --- |
| Age of the man | Independent | Quantitative/Continuous |  | Demographic questionnaire | Year |
| Education of the woman | Independent | Qualitative/Ordinal |  | Demographic questionnaire | Elementary High School, Diploma, University |
| Education of the man | Independent | Qualitative/Ordinal |  | Demographic questionnaire | Elementary High School, Diploma, University |
| Occupation of the woman | Independent | Qualitative/Nominal |  | Demographic questionnaire | Housewife, Employed |
| Occupation of the man | Independent | Qualitative/Nominal |  | Demographic questionnaire | Unemployed, Student, Employee, Worker, Freelance, Retired, Other |
| Economic status | Independent | Qualitative/Ordinal |  | Demographic questionnaire | Poor, Average, Good, Excellent |
| Number of pregnancies | Independent | Quantitative/Discrete |  | Demographic questionnaire | Number |
| Number of births | Independent | Quantitative/Discrete |  | Demographic questionnaire | Number |
| Number of miscarriages | Independent | Quantitative/Discrete |  | Demographic questionnaire | Number |
| Number of children | Independent | Quantitative/Discrete |  | Demographic questionnaire | Vaginal/Cesarean |
| Type of delivery | Independent | Qualitative/Nominal |  | Demographic questionnaire | Girl/Boy |
| Sex of the current baby | Independent | Qualitative/Nominal |  | Demographic questionnaire | Wanted/Unwanted |
| Wanted or unwanted pregnancy | Independent | Qualitative/Nominal |  | Demographic questionnaire | Number (by month) |
| Time elapsed since birth | Independent | Quantitative/Continuous |  | Demographic questionnaire | Poor/Average/Good |
| Supportive status of the individual | Independent | Qualitative/Ordinal |  | Demographic questionnaire | Year |

Timetable

| Row | Activities | Month |  |
| --- | --- | --- | --- |
| 1 | Stages of defending the proposal and making amendments and final approval | 1 month | \| 25 \| 24 \| 23 \| 22 \| 21 \| 20 \| 19 \| 18 \| 17 \| 16 \| 15 \| 14 \| 13 \| 12 \| 11 \| 10 \| 9 \| 8 \| 7 \| 6 \| 5 \| 4 \| 3 \| 2 \| 1 \| \| --- \| --- \| --- \| --- \| --- \| --- \| --- \| --- \| --- \| --- \| --- \| --- \| --- \| --- \| --- \| --- \| --- \| --- \| --- \| --- \| --- \| --- \| --- \| --- \| --- \| |
| 2 | Collecting data from the first stage (interviews, field notes), conducting interviews, coding and analyzing them | 3 month | \| 25 \| 24 \| 23 \| 22 \| 21 \| 20 \| 19 \| 18 \| 17 \| 16 \| 15 \| 14 \| 13 \| 12 \| 11 \| 10 \| 9 \| 8 \| 7 \| 6 \| 5 \| 4 \| 3 \| 2 \| 1 \| \| --- \| --- \| --- \| --- \| --- \| --- \| --- \| --- \| --- \| --- \| --- \| --- \| --- \| --- \| --- \| --- \| --- \| --- \| --- \| --- \| --- \| --- \| --- \| --- \| --- \| |
| 3 | Designing the intervention (second stage of the study) | 1 month | \| 25 \| 24 \| 23 \| 22 \| 21 \| 20 \| 19 \| 18 \| 17 \| 16 \| 15 \| 14 \| 13 \| 12 \| 11 \| 10 \| 9 \| 8 \| 7 \| 6 \| 5 \| 4 \| 3 \| 2 \| 1 \| \| --- \| --- \| --- \| --- \| --- \| --- \| --- \| --- \| --- \| --- \| --- \| --- \| --- \| --- \| --- \| --- \| --- \| --- \| --- \| --- \| --- \| --- \| --- \| --- \| --- \| |
| 4 | Implementing the intervention | 1 month | \| 25 \| 24 \| 23 \| 22 \| 21 \| 20 \| 19 \| 18 \| 17 \| 16 \| 15 \| 14 \| 13 \| 12 \| 11 \| 10 \| 9 \| 8 \| 7 \| 6 \| 5 \| 4 \| 3 \| 2 \| 1 \| \| --- \| --- \| --- \| --- \| --- \| --- \| --- \| --- \| --- \| --- \| --- \| --- \| --- \| --- \| --- \| --- \| --- \| --- \| --- \| --- \| --- \| --- \| --- \| --- \| --- \| |
| 5 | Collecting quantitative data and analyzing them statistically | 5 month | \| 25 \| 24 \| 23 \| 22 \| 21 \| 20 \| 19 \| 18 \| 17 \| 16 \| 15 \| 14 \| 13 \| 12 \| 11 \| 10 \| 9 \| 8 \| 7 \| 6 \| 5 \| 4 \| 3 \| 2 \| 1 \| \| --- \| --- \| --- \| --- \| --- \| --- \| --- \| --- \| --- \| --- \| --- \| --- \| --- \| --- \| --- \| --- \| --- \| --- \| --- \| --- \| --- \| --- \| --- \| --- \| --- \| |
| 6 | Writing the article and obtaining acceptance | 5 month | \| 25 \| 24 \| 23 \| 22 \| 21 \| 20 \| 19 \| 18 \| 17 \| 16 \| 15 \| 14 \| 13 \| 12 \| 11 \| 10 \| 9 \| 8 \| 7 \| 6 \| 5 \| 4 \| 3 \| 2 \| 1 \| \| --- \| --- \| --- \| --- \| --- \| --- \| --- \| --- \| --- \| --- \| --- \| --- \| --- \| --- \| --- \| --- \| --- \| --- \| --- \| --- \| --- \| --- \| --- \| --- \| --- \| |
| 7 | Presenting and reporting the research results | 2 month | \| 25 \| 24 \| 23 \| 22 \| 21 \| 20 \| 19 \| 18 \| 17 \| 16 \| 15 \| 14 \| 13 \| 12 \| 11 \| 10 \| 9 \| 8 \| 7 \| 6 \| 5 \| 4 \| 3 \| 2 \| 1 \| \| --- \| --- \| --- \| --- \| --- \| --- \| --- \| --- \| --- \| --- \| --- \| --- \| --- \| --- \| --- \| --- \| --- \| --- \| --- \| --- \| --- \| --- \| --- \| --- \| --- \| |

24 Month

Personnel costs

No data was recorded for Personnel costs.

The cost of equipment and materials

No data was recorded for The cost of equipment and materials.

The cost of tests and and specialized services (within the university)

No data was recorded for The cost of tests and and specialized services (within the university).

The cost of tests and and specialized services (outside the university)

No data was recorded for The cost of tests and and specialized services (outside the university).

Travel Cost

No data was recorded for Travel Cost.

Other costs

No data was recorded for Other costs.

How to finance the research project

No data was recorded for How to finance the research project.

Ethical considerations

**Ethical considerations and problems of the project**

The lack of trust of study participants in the researcher

Concerns about revealing their secrets and opinions

Concerns about the obligation to continue cooperation until the end of the project even if they lose their desire to cooperate during the project

Concerns about the reason for conducting the interview and where the results will be presented

**Resolution of ethical problems**

(1) Obtaining permission from the Vice President of Research, Tehran School of Nursing and Midwifery

(2) Obtaining permission from the Vice President of Research, Kermanshah University of Medical Sciences

(3) Obtaining permission from the heads of hospitals affiliated with Kermanshah University of Medical Sciences

(4) Obtaining informed consent from participating patients

(5) Introducing the researcher to the participants

(6) Clarifying the objectives and methods of conducting the research for each participant

(7) All participants in the study are assured that all information provided by them will be kept confidential and will be kept confidential by the researcher.

(8) Mentioning the anonymity of the interviews

(9) Participants are reminded that participation in the study is completely voluntary and they are allowed to decide at any point in the study whether to continue or discontinue cooperation with the researcher.

(10) Respecting integrity

(11) Obtaining ethics approval for conducting the research

KTE

1. **Is the research conducted at the request of the employer and is part or all of its funding provided by the client?**

No

**Please enter the name of the organization and upload its documentation in the project attachments section.**

**2) Is the research conducted at the request of the employer and is all of its funding provided by the university?**

Yes

**Please enter the name of the organization and upload its documentation in the project attachments section.**

Tehran University of Medical Sciences, Faculty of Nursing and Midwifery

**3) Do the direct beneficiaries of the research results actively participate in conducting the research?**

Yes

**4) Is the research based on an organization's stated priorities?**

Yes

**Please enter the name of the organization and upload the priorities file in the project attachments section.**

Faculty of Nursing and Midwifery, Tehran University of Medical Sciences

**5) Is it aimed at solving a health challenge?**

Yes

**Explain how the research findings can address the challenge.**

If comprehensive postpartum supportive care programs are adopted with comprehensive coverage for women, it can be assured that women’s needs during the critical postpartum period are met. This requires conducting an assessment, examining the concerns and various dimensions of women’s problems in the postpartum period from their own perspective, and examining the impact of such a program on the health of women and their infants, as well as parental anxiety and self-confidence; and the economic impact on health services for women and their families. Since careful planning and new evaluation are needed to ensure the adequacy of postpartum care. Approaches should be adopted that fully address the problems that surround fathers and mothers in accessing comprehensive postpartum care with a comprehensive supportive care approach, and should not be limited to the limited care provided to women after discharge from the hospital (16). This requires conducting qualitative research to explain the needs and problems of couples at this time. On the other hand, given that no qualitative research has been conducted on this issue in Iran so far and previous studies have only quantitatively assessed the quality of postpartum care, therefore, in line with the research priority of the Department of Midwifery and Reproductive Health, Tehran University of Medical Sciences, on designing, implementing, monitoring, and evaluating interventions related to reducing maternal and neonatal morbidity after childbirth, this study aims to use a combined approach to explain the supportive care needs of couples in the postpartum period and design and implement a needs-based intervention to improve health services in the postpartum period. If the designed intervention is effective, it will be made available to policymakers and health managers to use in order to improve the quality of postpartum care.

**6) Does the research result have the potential to create change and impact?**

**Yes**

**The type of effect and target group of the change should be written as an explanation.**

**Target group:** Women and their partners in the postpartum period

Type of effect: Helping to improve the adequacy of postpartum care and proposing a comprehensive supportive care program with comprehensive coverage

**7) In order for this research to lead to change, should it be placed alongside the results of other research so that they can make specific recommendations?**

Yes

**Another research needs to be identified**

**-** Review studies in the field of supportive care needs of mothers and fathers in different ethnicities and nationalities

- Conduct clinical trials to evaluate the effectiveness of the proposed intervention in this plan

**8) Can the research result lead to the production of an initial product (prototype) or the registration of a patent?**

No

**9) In the case of the present study, is your answer to at least one of the above questions positive?**

Yes

Self-deceleration of research products

No data was recorded for Self-deceleration of research products .

attachments

| **Name** | **Type** | **Subject** | **Date** | **Download** |
| --- | --- | --- | --- | --- |
| Informed consent.doc |  | Informed consent form | 2024/02/01 19:19:14 | [Download](file://\\j-fs04\J-PLOS-L\Production\PONE\pone.0350038\FROM_CLIENT\Accepted_manuscripts\pone_637862bf-78aa-482b-a38f-14f0a97e4dc3\download?xyz=s-9TEuZMjPUEj1I2WVwnU3i5uHRfxtp7Ti-ZUylK8f73J0N2KycZ2OJyQ3Ed7DQVX2izp253ELEeTSwUCGLi-1QB5W6DPZbGbZgsRHWlLzxbna1q7B7iX3f41DXoSBiX&csrf_token=MTc1MjY4OTQ0Nzg0ZU1ZUGRxR2lKakZzNTN6Umkwc3RZOGluVjBTR0JC) |
| Research priorities of midwifery and reproductive health education group.pdf |  | Research priorities of midwifery and reproductive health education group | 2024/02/01 22:11:55 | [Download](file://\\j-fs04\J-PLOS-L\Production\PONE\pone.0350038\FROM_CLIENT\Accepted_manuscripts\pone_637862bf-78aa-482b-a38f-14f0a97e4dc3\download?xyz=K7NNoUDCOoGb33ZeaymWHDqsoqayonfu7x8eq8RB1TE3W2mydlAuCk28EEbhOYsHvAfy8VQpFOmv9wbTD1Gz9pE-nO66hn-ZGA3qMx4Ma63qwCfxNHY8KafdEukE1AvC&csrf_token=MTc1MjY4OTQ0Nzg0ZU1ZUGRxR2lKakZzNTN6Umkwc3RZOGluVjBTR0JC) |
| Proposal approval form.jpg |  | Proposal approval form | 2024/02/01 22:12:45 | [Download](file://\\j-fs04\J-PLOS-L\Production\PONE\pone.0350038\FROM_CLIENT\Accepted_manuscripts\pone_637862bf-78aa-482b-a38f-14f0a97e4dc3\download?xyz=IEsn-O0c5CEyXIggZ1rXA07wOFyzAS_6d3wZb97yzgHsycAhu-cKKcV36s-7jqmRFtDfNTANoRoP0VoYX_IGS5eklBPQ2jWK3MuHoyjf1T_UxPJQqHvcyERxb0eKeMa6&csrf_token=MTc1MjY4OTQ0Nzg0ZU1ZUGRxR2lKakZzNTN6Umkwc3RZOGluVjBTR0JC) |
| Minutes of the proposal defense meeting.pdf |  | Minutes of the proposal defense meeting | 2024/02/04 08:23:11 | [Download](file://\\j-fs04\J-PLOS-L\Production\PONE\pone.0350038\FROM_CLIENT\Accepted_manuscripts\pone_637862bf-78aa-482b-a38f-14f0a97e4dc3\download?xyz=JURItHo2nQku7WXkPN7mFUZm5MKDh1H244cKGJl6H7k4P0XYTKXVGHAjNbdinHkpTusvvGS7DWPwTuYVQzsFmwNHMmWinmjOJ4kOU6r_SEzQVIfMMhS9CIPu4NMlXzDY&csrf_token=MTc1MjY4OTQ0Nzg0ZU1ZUGRxR2lKakZzNTN6Umkwc3RZOGluVjBTR0JC) |
| Descriptions related to the quantitative part of the study (questionnaire and sample size in the quantitative part).docx |  | Descriptions related to the quantitative part of the study (questionnaire and sample size in the quantitative part) | 2024/02/07 17:52:54 | [Download](file://\\j-fs04\J-PLOS-L\Production\PONE\pone.0350038\FROM_CLIENT\Accepted_manuscripts\pone_637862bf-78aa-482b-a38f-14f0a97e4dc3\download?xyz=8BXbMYzAQmIpCK-bHF9FlCQ-mHRIAnr_IvdKadtBrUnsHvrbM0CqgWKnoidUubWyta6OeKIy0DcdJOv53-RCGZ5HyQqWBrdmd6Jmr2WtbiAnpOMp8U7wPGyFSF4pMexm&csrf_token=MTc1MjY4OTQ0Nzg0ZU1ZUGRxR2lKakZzNTN6Umkwc3RZOGluVjBTR0JC) |
| Sample interview questions.docx |  | Sample interview questions | 2024/02/07 17:53:22 | [Download](file://\\j-fs04\J-PLOS-L\Production\PONE\pone.0350038\FROM_CLIENT\Accepted_manuscripts\pone_637862bf-78aa-482b-a38f-14f0a97e4dc3\download?xyz=T3Pk7JV1yIgxYW_gFOMePSn0CDV7qGwv3_x8HAcS_sC-ysRhhu5YINratf7bFIfL5Cn3uwzo4ZkVfUTx9PIqu7KwcT8ZYziR-FG719xiuIaddJE_tJ5dfRIuZlHshkjh&csrf_token=MTc1MjY4OTQ0Nzg0ZU1ZUGRxR2lKakZzNTN6Umkwc3RZOGluVjBTR0JC) |
| proposal.docx |  | proposal | 2024/02/28 11:25:58 | [Download](file://\\j-fs04\J-PLOS-L\Production\PONE\pone.0350038\FROM_CLIENT\Accepted_manuscripts\pone_637862bf-78aa-482b-a38f-14f0a97e4dc3\download?xyz=1yxNX6jaPo3NOu5htp5J04s-FJ0ezwclqAbHBdQ3wp1soc5-csNa5JGktpo1GpeBMgfH1W6pLBsD2WtT6lUElhuS5lA2vcAQJbCXz3cFPVIouunJ-a_ghINRdgeOh6CK&csrf_token=MTc1MjY4OTQ0Nzg0ZU1ZUGRxR2lKakZzNTN6Umkwc3RZOGluVjBTR0JC) |
