# Supplementary material for: The supportive care needs of Iranian couples during postpartum hospitalization: A protocol of design, implementation and evaluation of intervention
Source: PLoS One. 2026 Jun 3;21(6):e0350038. doi: 10.1371/journal.pone.0350038 (PMC13232809; doi:10.1371/journal.pone.0350038)
Supplement: S4 File — (PDF) [file pone.0350038.s003.docx]

**Title :** Exploring the supportive care needs of Iranian couples during postpartum period: Designing, Implementing and Evaluating the Intervention

**Tracking Code:** 70979

**Researcher :** Zahra rastad

**Specialty:** -

**Project Code:** 40111151006

**Ethics Code:** IR.TUMS.FNM.REC.1402.238

**Date of Registration:** 2024/02/01 17:51:02

**Postage date:** 2024/02/01 22:15:03

**Edit date:**

**The second target center:** School of Nursing and Midwifery

**The second target center:**

**The third target center:**

General specifications and design abstract

**عنوان فارسی پایان نامه**

تبیین نیازهای مراقبت حمایتی زوجین ایرانی در دوره پس از زایمان: طراحی، اجرا و ارزشیابی مداخله

**English Title**

Exploring the supportive care needs of Iranian couples during postpartum period: Designing, Implementing and Evaluating the Intervention

**مقطع**

Ph.D Thesis

**شماره دانشجویی**

40111151006

**کلید واژه ها**

نیازهای مراقبت حمایتی- زوجین-دوره پس از زایمان

**نوع پایان نامه**

Basic-Applied

**نوع مطالعه**

other

**خلاصه ضرورت اجرای پایان نامه**

دوره پس از زایمان مملو از تغییرات فیزیولوژیک و هورمونی است و می تواند مادران را با چالش های اجتماعی، عاطفی و عملکردی مواجه کند. به طور کلی عواملی که با نارضایتی زنان از مراقبت‌های پس از زایمان ارتباط دارند بازتاب تجارب زنان هستند. میزان حساسیت مراقبین و این که تا چه حد نگرانی های زنان جدی گرفته می شود، تعجیل در ارایه مراقبت ها توسط پرسنل، طول مدت بستری در بیمارستان پس از زایمان و عدم وجود تدابیر مربوط به مشاوره و مراقبت های حمایتی و پشتیبانی مهم ترین عوامل ریشه ای هستند که با نارضایتی در ارتباطند و به عنوان مهم ترین موانع دستیابی به سلامت مطلوب در حوزه خدمات حول و حوش تولد و پس از زایمان تلقی می شوند. از آن جا که برنامه ریزی دقیق و ارزیابی جدید برای اطمینان از کفایت مراقبت های پس از زایمان مورد نیاز است. باید رویکردهایی اتخاذ شود که مشکلاتی که پدران و مادران را در دستیابی به مراقبت های جامع پس از زایمان احاطه کرده است به طور کامل و با رویکرد مراقبت حمایتی جامع پوشش داده شود و نباید صرفا به مراقبت های محدود ارایه شده به زنان پس از ترخیص از بیمارستان بسنده نمود. این امر مستلزم انجام پژوهش های کیفی در راستای تبیین نیاز ها و مشکلات زوجین در این برهه زمانی است. از طرفی با توجه به اینکه تاکنون در ایران پژوهشی کیفی حول محور این موضوع انجام نشده و مطالعات قبلی صرفا به صورت کمی، کیفیت مراقبت های پس از زایمان را مورد سنجش قرار داده اند، درراستای اولویت پژوهشی گروه مامایی و سلامت باروری دانشگاه علوم پزشکی تهران، مبنی بر طراحی، اجرا،پایش و ارزشیابی مداخلات مرتبط با کاهش موربیدیته مادران و نوزادان پس از زایمان این مطالعه قصد دارد با یک رویکرد ترکیبی به تبیین نیازهای مراقبت حمایتی زوجین در دوره پس از زایمان پرداخته و یک مداخله مبتنی بر نیاز در راستای ارتقا خدمات سلامت در دوران پس از زایمان را طراحی و اجرا نماید.

**خلاصه روش اجرا و شیوه های تحلیل**

پژوهش حاضر یک مطالعه ترکیبی چند مرحله ای است، این پژوهش دارای سه بخش کیفی – طراحی مداخله و کمی خواهد بود و در سال 1402-1403 در دانشگاه علوم پزشکی کرمانشاه انجام خواهد شد. جامعه پژوهش، زنان بستری و دریافت کننده مراقبت در بخش های پس از زایمان بیمارستان های وابسته به دانشگاه علوم پزشکی کرمانشاه(که تجربه زایمان به شیوه زایمان طبیعی یا سزارین را داشته اند) ونیز زنان مراجعه کننده به مراکز بهداشتی درمانی که حداکثر 8 هفته از زمان زایمان آن ها گذشته است و همسران آنان، ارایه دهندگان خدمات سلامت باروری، مدیران و سیاست گزاران حوزه سلامت باروری و حوزه سلامت زنان، روان شناسان و ماماهای شاغل در خارج از کشور هستند.
ابتدا مطالعه کیفی متوالی اکتشافی به روش تحلیل محتوای مرسوم باهدف تبیین نیازهای مراقبت حمایتی مادران و پدران در دوره پس از زایمان انجام خواهد شد و پژوهش کیفی با رویکرد تحلیل محتوا خواهد بود که ابتدا به تبیین نیازهای مراقبت حمایتی مادران و پدران در دوره پس از زایمان از طریق مصاحبه نیمه ساختاریافته عمیق با زنان و همسران آن ها و ارایه دهندگان خدمات مراقبتی به آنها، خواهیم پرداخت. نمونه گیری در هر دو گروه به روش مبتنی بر هدف با حداکثر تنوع(سنی ، رتبه بارداری، شغلی، نوع زایمان، تحصیلات و...) صورت خواهد گرفت . در این پژوهش محقق از تحلیل محتوای قراردای به روش ژانک و ویلموس (برای طبقه بندی داده ها) استفاده خواهد نمود.
در مرحله دوم مطالعه با استفاده از نتایج مرحله کیفی، مسایل و نیازهای مراقبت حمایتی زنان و همسران آن ها در جلسه ای متشکل از متخصصان سلامت باروری ، سیاستگذاران سلامت باروری ، اساتید راهنما و داورها به شیوه گروه اسمی مورد اولویت بندی قرار خواهند گرفت و سپس مروری بر متون برای یافتن یک مداخله ای مناسب جهت اجرا در بخش کمی مطالعه، صورت می پذیرد .
در مرحله سوم مطالعه یا بخش کمی با توجه به داده های کیفی و اولویت های پیشنهادی در جلسه گروه اسمی (که لینک بین بخش کیفی و کمی مطالعه می باشد) ومروری بر متون گسترده ، در نهایت یک مداخله مبتنی بر نیاز و اثربخش طراحی خواهد شد وسپس مداخله طراحی شده اجرا و ارزشیابی مداخله مرتفع کننده نیازهای مراقبت حمایتی مادران و پدران در دوره پس از زایمان انجام خواهد شد. در نهایت داده های کمی با استفاده از نرم افزار spss مورد تجزیه و تحلیل قرار خواهند گرفت.

Record

**اهداف کلی , اختصاصی و کاربردی**

اهداف اصلی طرح :
1- تبیین نیازهای مراقبت حمایتی زوجین ایرانی در دوره پس از زایمان
2-طراحی، اجرا و ارزشیابی مداخله ی مبتنی بر مهمترین نیازهای مراقبت حمایتی زوجین ایرانی در دوره پس از زایمان

اهداف فرعی طرح :
اهداف بخش کیفی مطالعه
1-تبیین نیاز مراقبت حمایتی زوجین ایرانی در دوره پس از زایمان
2-تبیین نیاز مراقبت حمایتی از دیدگاه ارایه دهندگان خدمات(پزشکان، ماماها، پرستاران و...) در دوره پس از زایمان
3-تبیین نیازمراقبت حمایتی از دیدگاه صاحب نظران (مطلعین کلیدی از قبیل مدیران و سیاست گزاران)
4-تبیین تسهیل گر ها و موانع مراقبت حمایتی زوجین در دوره پس از زایمان
اهداف بخش کمی مطالعه :
1-تعیین مهم¬ترین نیازهای مراقبت حمایتی زوجین در دوره پس از زایمان بر اساس نتایج استخراج شده از بخش کیفی و مرور متون با استفاده از پانل صاحب نظران
2-طراحی مداخله ی مبتنی بر نیازهای مراقبت حمایتی زوجین در دوره پس از زایمان
3-تعیین روایی محتوای مداخله طراحی شده (چنانچه در مداخله نیاز به طراحی محتوی باشد روایی محتوی توسط متخصصین انجام می شود).
4-اجرا و ارزشیابی مداخله ی مبتنی بر نیازهای مراقبت حمایتی زوجین در دوره پس از زایمان
این اهداف در مراحل بعدی کامل می شوند.
اهداف کاربردی پژوهش:
مداخله طراحی شده در این بخش، که مبتنی بر نیازهای مراقبت حمایتی زوجین ایرانی در دوره پس از زایمان است در صورت اثربخشی مداخله، در اختیار سیاست گذاران و مدیران بهداشتی قرار داده خواهد شد تا در جهت ارتقا کیفیت مراقبت های پس از زایمان از آن استفاده نمایند.

**مقدمه-بیان مساله**

دوره پس از زایمان مملو از تغییرات فیزیولوژیک و هورمونی است و می تواند مادران را با چالش های اجتماعی، عاطفی و عملکردی مواجه کند (1, 2) اگرچه مادری تجربه ای خوشایند و منحصر به فرد است، اما گذار به مادری گاه با استرس و تنش همراه است، زیرا با تغییرات فراوان و سریع درون فردی و بین فردی در ابعاد مختلف همراه است که زن باید قادر به سازگاری با تجارب این تغییرات باشد (3).
زنان در دوره پس از زایمان گاه با عوامل خطر زای فیزیکی و بیولوژیکی (سلامت جسمانی ضعیف)، روانی (افسردگی و اندوه پس از زایمان، رویدادهای استرس زا زندگی)، زایمان و عوامل مربوط به نوزاد (بارداری ناخواسته، سزارین اورژانسی، استرس القا شده در طول زایمان طبیعی و ترومای زایمانی)، اجتماعی-جمعیتی (پایین بودن سن مادر) و عوامل فرهنگی (حمایت اجتماعی کم) روبه رو هستند (4). در حالی که آن ها نیاز دارند که تجربه خود را ازاین بخش اززندگی با دیگران به اشتراک بگذارند و به آنها اطمینان داده شود که درک شده اند (4).
شناسایی نیازهای حمایتی و انتظارات مادران جدید برای رفاه و بهبودی آنان در دوره پس از زایمان مهم است. نیاز های حمایتی که به جنبه های مختلف جسمی، شناختی، عاطفی و روانشناختی نیاز های افراد در شرایط خارج از معمول و ویژه زندگی می پردازد، هدف اولیه و محور مراقبت های زن محور و بهبود کیفیت زندگی زنان در دوره پس از زایمان است. ابعاد کلیدی مراقبت حمایتی شامل مدیریت درد و علایم، حمایت عاطفی و روان شناختی، ارایه اطلاعات در زمینه های مورد نیاز به زنان، بهبود ارتباطات ، توانایی تصمیم گیری، رسیدگی به نیاز های عملی( نگرانی های مالی، دسترسی به منابع و ....)، احترام به کرامت و خودمختاری، و ارایه مراقبت های هماهنگ و یکپارچه است (5).
دغدغه های زنان در دوره بعد از زایمان دربرگیرنده مضامین مشترک جهانی از قبیل اضطراب، ترس، احساس تنهایی، نگرانی در مورد شایستگی و توانمندی خود برای فرزندپروری و پذیرش نقش مادری ، تصویر ذهنی منفی از بدن خود((body image ، توانایی برقراری روابط رضایت بخش جنسی و بازسازی روابط با همسر و سایر اعضای خانواده و موضوعات مهم دیگر از این قبیل است. این مسایل در مادرانی که برای اولین بار زایمان را تجربه می کنند نسبت به کسانی که قبلا تجربه مادر شدن را داشته اند کاملا محرز تر و دارای اهمیت بیشتری است . این مضامین در سطح ملی و بین المللی، بازتاب یافته از شواهد و متون موجود است (1, 2, 6-9).
هدف اصلی مراقبت های مطلوب پس از زایمان ، حفظ و ارتقای سلامت زن و نوزاد او و ایجاد محیطی است که به خانواده و جامعه برای مرتفع ساختن طیف وسیعی از نیازهای بهداشتی و اجتماعی مرتبط کمک و حمایت می کند. آن چه ضرورت دارد این است که محتوای مراقبت های پس از زایمان مفهوم سلامت مطلوب را در بر بگیرد و در راستای کلیه ابعاد سلامت از نظر سازمان جهانی بهداشت باشد (10).
اما شواهد نشان می دهد که برخی از زنان مراقبت‌هایی را که درهنگام حضور در بخش پس از زایمان دریافت می کنند ناکافی تلقی می کنند. از جمله در مطالعه سیمبر و همکاران که بر روی زنان بستری در بیمارستان های منتخب دانشگاه علوم پزشکی کردستان انجام شد مراقبت ها از دیدگاه زنان در حوزه حمایت روحی روانی ضعیف ارزیابی شده بود(11). همچنین در مطالعه ی توصیفی دیگری سیمبر و همکاران کیفیت ارایه مراقبت های پس از زایمان در بیمارستان های تابعه ی دانشگاه علوم پزشکی شهید بهشتی ضعیف گزارش کردند (12). علی دوستی و همکاران نیز در بررسی رضایت مندی زنان در دوره پس از زایمان از خدمات و امکانات زایشگاه بیمارستان هاجر شهرکرد دریافتند که رضایتمندی افراد از خدمات انسانی در سطح پایینی است و اظهار داشتند که حجم کاری زیاد پرسنل سبب کاهش تمرکز آن ها در ارایه بهتر خدمات شده است (13).
به طور کلی عواملی که با نارضایتی زنان از مراقبت‌های پس از زایمان ارتباط دارند بازتاب تجارب زنان هستند. میزان حساسیت مراقبین و این که تا چه حد نگرانی های زنان جدی گرفته می شود، تعجیل در ارایه مراقبت ها توسط پرسنل، طول مدت بستری در بیمارستان پس از زایمان و عدم وجود تدابیر مربوط به مشاوره و مراقبت های حمایتی و پشتیبانی (14) مهم ترین عوامل ریشه ای هستند که با نارضایتی در ارتباطند و به عنوان مهم ترین موانع دستیابی به سلامت مطلوب در حوزه خدمات حول و حوش تولد و پس از زایمان تلقی می شوند (14).
در مطالعه ای مروری که با هدف بررسی دیدگاه زنان در رابطه با مراقبت های بعداز زایمان بیمارستانی انجام شده است، زنان ارایه مراقبت های پس از زایمان را در مقایسه با سایر مراقبت ها ، دارای اولویت کمتری درک کرده بودند. (مراقبت های پس از زایمان در اولویت مراقبت های ارایه شده توسط Care provider ها نیست) (15) دیگر موضوعاتی که زنان تحت مطالعات به آن اشاره نموده اند شامل شلوغی و گاه آشفتگی بخش های پس از زایمان و عدم انعطاف پذیری در رفع نیاز های فردی زنان بود . محدودیت تعداد پرسنل و تعداد بالای بازدید کنندگان(ملاقات کنندگان)، عدم اجازه به مادر جهت در آغوش کشیدن و تماس با نوزاد بلافاصله پس از زایمان (و در موارد خاص از جمله نیاز به بستری نوزاد برای مدت طولانی تر) و نیز چالش های حمایتی از قبیل عدم وجود امکانات مشاوره در راستای موضوعاتی مانند سازگاری باا تغییرات در روابط صمیمی و خانوادگی خود، از سرگیری روابط جنسی، مهارت تطابق با فرزندپروری، و مقابله با ترومای زایمانی تجربه شده، و رفع نگرانی هایی مانند آسیب به دستگاه تناسلی، تصویر ذهنی از بدن و غیره از دیگر موانع ارایه مراقبت های زن محور ذکر شده است (15-22).
به علاوه بندرت پیش می آید که در طول مدت بستری زنان در بخش پس از زایمان پدرها، فرصت و امکانات این را داشته باشند که شب ها در بیمارستان در کنار همسر و فرزند خود باقی بمانند (15, 16). و یا به آن ها اجازه داده شود در بحث های مراقبتی مشاوره ای وارد شوند (مراقبت های مشاوره ای را در کنار همسر خود دریافت کنند) . در صورتی که نقطه عطف برای جلب همکاریهای زوجی برای مراقبت از نوزاد، دوره بعداز زایمان است. مردان خواهان مشارکت بیش تری در مراقبت از فرزند هستند اما این امر نیاز به حمایت در گذار به پدر شدن دارد. مطالعات اندکی توضیح داده اند که چه مقوله هایی "حمایت" برای پدران تلقی می شود و ممکن است با حمایتی که مادران به دنبال آن هستند متفاوت باشد. مشکلات سلامت روان پری ناتال به میزان مشابه زنان، برای مردان رخ می دهد اما مردان اغلب درگیر خدمات و مراقبت های بهداشتی نیستند.و همان طور که مسجل است سلامت روان ضعیف پدران بر رشد عاطفی و شناختی کودک و روابط آن ها با همسران خود تاثیر سوء می گذارد. در واقع بررسی ها نشان می دهد که پدران به حمایت از همه سطوح اکوسیستم از جمله تغییرات سیاست گذاری، اجتماعی فرهنگی، و حمایت از طرف خانواده و همسرو به ویژه خدمات و مراقبت های بهداشتی نیاز دارند (23).
از دیگر مسایل مطرح شده شامل عدم دریافت مراقبت های حرفه ای با کیفیت و در سطح مورد انتظار ، ناسازگاری و عدم همخوانی توصیه های ارایه شده به آنان از مراقبین مختلف، عدم وجود امکانات مشاوره، و مراقبت های تخصصی است (24).
با این حال، تجربیات منفی یا کمبودهای درک شده در مراقبت منجر به این نشده است که زنان بخواهند زودتر بیمارستان را ترک کنند. بیشترزنان تمایل دارند تا زمانی که از توانمندی خود در خصوص مراقبت نوزاد مطمئن نشده اند، در بیمارستان بمانند و در واقع از زمان خود برای یادگیری مهارت های مرتبط با شیردهی و مراقبت کلی از نوزاد و افزایش اعتماد به نفس خود در این زمینه استفاده کنند و به تبادل تجربه با سایر زنان به ویژه با زنان مولتی پار که مهارت بیشتری در زمینه های مذکور دارند بپردازند و آگاهی های لازم را از مراقبین کسب کنند . بر اساس شواهد موجود پرداختن به این مسایل و اختصاص زمان کافی از سوی مراقبین یکی از دغدغه های اصلی زنان در دوره پس از زایمان محسوب می شود و در واقع زنان مایل به شرکت در کلاس ها و استفاده از خدمات پشتیبانی پس از ترخیص (به عنوان مثال کلینیک های شیردهی، مراکز ارایه دهنده خدمات ارتقا سلامت روان، مشاوره های فردی، مشاوره جنسی و..) هستند. یافته ها نشان می دهد که به نظر می رسد برای بسیاری از زنان، ماندن در بیمارستان برای روزهای بعد از تولد تنها گزینه ایمن موجود برای آنان و نوزادشان است (8, 9, 16).
یک دیدگاه ثابت وجود دارد که حضور فیزیکی و در دسترس بودن پشتیبانی حرفه ای و مراقبت های حمایتی تاثیر بسیار بسزایی در کاهش نگرانی ها و رفع نیاز های زنان در کلیه ابعاد مدنظر آنان دارد.
به نظر می رسد که تعدادی از عوامل وجود دارد که می تواند به تلقی زنان از بیمارستان به عنوان امن ترین و مناسب ترین مکان بعد از زایمان کمک کند. این عوامل شامل میزان تدابیر همه جانبه برای پوشش دهی و پاسخ گویی به ابعاد مختلف نیاز های زنان پس از زایمان و القای حس دریافت حمایت کافی از سوی پرسنل و ارایه دهندگان خدمت است (16) و بالعکس برخی از عوامل اجتماعی می توانند نتیجه منفی در دیدگاه زنان داشته باشند. عمده این عوامل حول محور زیر ساخت اجتماعی زایمان در کشور، پزشکی سازی فرایند تولد و بارداری، الگوی تکنوکراتیک (زیست پزشکی) سیستم بهداشتی درمانی و عدم مشارکت و دخیل دانستن زنان در تصمیم گیری های پزشکی در پروسه بهداشت و درمان است (25).
مفهوم رفتن به بیمارستان برای بچه دار شدن و ماندن در بیمارستان پس از زایمان از نظر فرهنگی به یک هنجار اجتماعی در این خصوص تبدیل شده که زایمان یک پروسه خطرناک، آسیب رسان و غیرقابل کنترل است (26). نویسندگان استدلال می کنند که این ساخت و ساز در ارتباط با پزشکی سازی تولد، و تخریب فزاینده احساس شایستگی زنان در رابطه با تولد و مراقبت از نوزاد تازه وارد، به خصوص با کمبود اطلاعات نهادینه شده در فرهنگ رخ داده است (27)(با وجود این پیش زمینه، ترس در وجود مادران در مورد سلامت خود و نوزاد تازه متولد شده بدور از نظارت های پزشکی و حمایتی مورد انتظار، کاملا قابل انتظار و بدیهی بنظر می رسد (27).
در حقیقت شیوه ارایه مراقبت ها از سوی ارایه دهندگان خدمت (به ویژه ماماها) و نحوه رفتار آن ها با مادران پس از تولد این دیدگاه را برای زنان ایجاد کرده است که مراجعه به بیمارستان های دارای پشتیبانی پزشکی 24 ساعته در حین تولد و پس از آن به صورت مطمئن و امن خواهد بود اما در عمل میان انتظار و آن چه در واقع رخ می دهد فواصل زیادی مشاهده می شود (26). آن چه از مصاحبه های ساختاریافته با زنان در دوره پس از زایمان به دست می اید این است که آنان مراقبین بهداشتی (ماماها و پرستاران) را جهت پوشش دهی نگرانی های خود کافی نمی دانند و نمی توانند برای رفع نیاز های خود به آنان اعتماد و بسنده کنند . ممکن است اقداماتی از قبیل پزشکی‌سازی زایمان زنان را به این سمت و سو سوق داده باشدکه نیاز به این داشته باشند که متخصصان مراقبت های بهداشتی و پزشکان در اطراف آنها به منظور تسکین اضطراب ها یا ترس های آنها در طول انتقال به مادر شدن و فرزندپروری حضور داشته باشند (16).
به علاوه بر اساس شواهد موجود، زنان ترجیح می دهند خدمات مراقبت بهداشتی را در جایی که ارتباط ارایه دهندگان مراقبت با آن ها توام با احترام و ارایه مطلوب مراقبت باشد دریافت کنند و مراقبت محترمانه از دیدگاه آنان شامل مراقبت های حمایت کننده و مبتنی بر نیاز های زنان است (24).
توصیف مراقبت های غیر محترمانه از سوی آنان شامل آزار کلامی یا فیزیکی و یا القای احساس سلطه و قدرت به مراجع از سوی مراقبت دهنده بود ومطالعات نشان می دهند که زنان آسیب پذیر( نوجوانان، زنان مبتلا به وضعیت اجتماعی-اقتصادی ضعیف و زنان HIV مثبت) احتمال بیشتری دارد که مراقبت های غیر محترمانه دریافت کنند (24).
مراقبت های پری ناتال از سوی ارایه دهندگان خدمت با توجه به تا حدودی ماهیت تهاجمی آن ، دارای پتانسیل آسیب رساندن یا ایجاد آسیب مجدد است (18). مراقبت های مامایی اغلب شامل پاسخ دادن به سوالات حساس ،الزام به تعویض لباس، بررسی ها و پروسیجرهایی باشد که می تواند تهاجمی تلقی شود.موارد فوق پتانسیل ایجاد فلاش بک و آسیب دیدگی مجدد زنان را دارد. این زنان ممکن است سطح بالای اضطراب ، ناتوانی عاطفی، و گسستگی در طول مراقبت های مامایی را نشان دهند و از طرفی هم با توجه به شیوع بالای رفتارهای غیرمحترمانه نیاز به پاسخ گویی در قالب حمایت و تسکین بخشی را اجتناب ناپذیر می کند (18, 28). مطالعه هریس و آیرز همچنین نشان داد که قوی ترین پیش بینی کننده ایجاد PTSD مربوط به تولد، مشکلات بین فردی با ارایه دهندگان مراقبت و تجربه کمبود حمایت بود (28). در حالی که ارایه دهندگان مراقبت ممکن است اقدامات و تعاملات خود را روتین در نظر بگیرند برخی از زنان آنها را آسیب زننده میدانند (28) این پدیده منجر به معرفی اصطلاح حقوقی «خشونت مامایی» در برخی از کشورها شده است.. والمیر و همکاران اشاره کرده اند که زنان از اصطلاح " birth rape " برای توصیف تجربیات خشونت زایمان استفاده می کردند . مطالعات کیفی که به بررسی تجربیات زنان از آسیب‌های روانی زایمان می‌پردازد مشخص کننده نحوه تعامل با ارایه دهندگان مراقبت به عنوان یک عامل مهم تر از مداخله پزشکی یا نوع زایمان است (28).
با توجه به واقعیت های چندگانه و نیازهایی که به آن ها اشاره شد می توان به این نتیجه رسید که مراقبت های بهداشتی و درمانی کشور در دوره پس از زایمان با غفلت نسبی از حمایت روانی و اجتماعی مادران و پدران همراه بوده است. شواهد قابل توجهی مبنی بر عدم تطابق بین ارایه خدمات بهداشتی درمانی و حمایت مورد نظر مادران، وجود دارد و شکاف ثابتی بین آن چه مادران از مراقبت ها در طول پروسه زایمان و پس از آن انتظار دارند و آن چه ارایه می شود، وجود دارد. همچنین مطالعاتی که در این زمینه اختصاصا بر روی پدران انجام شده باشد بسیار اندک است (29).
کالج آمریکایی زنان و زایمان (ACOG) غربالگری روتین سلامت روان پس از زایمان را به عنوان بخشی از پیگیری جامع پس از زایمان توصیه می کند . این در حالی است که خلق و خو و بهزیستی عاطفی فقط یکی از 9 حوزه توصیه شده برای پوشش و ارزیابی در طول ویزیت های بارداری و پس از زایمان را شامل می شود . حوزه های دیگر توصیه شده تمرکز بر مراقبت از نوزاد و مادر و سلامت و ریکاوری جسمانی مادر است ، نگرانی های مربوط به سلامت روان و تجارب روانی اجتماعی مادر ممکن است زمان و توجه کافی را برای پرداختن و رسیدگی به آن ها دریافت نکند . علاوه بر این، غربالگری سلامت روان به صورت جهانی اجرا نمی شود و گاهی اوقات به نوعی اجرا می شود که بر اساس دستورالعمل های توصیه شده نیست (29).
مساله بعدی نیاز به انعطاف پذیری در مراقبت پس از زایمان است. زنان تمایل دارند که گزینه های مراقبتی متفاوتی برای آن ها وجود داشته باشد این مساله بخصوص در مادران نخست زا صدق می کند. زنانی که فرزند دوم یا بعدی خود را به دنیا می آورند ممکن است کمتر به حمایت و آموزش پس از زایمان نیاز داشته باشند. این دیدگاه با تجربه زنان و اعتماد به نفس بیشتر در مراقبت از فرزندان بعدی و نیاز کمتر به امنیت و پشتیبانی مستمر حرفه ای مطابقت دارد. زنانی که اولین فرزند خود را به دنیا می آورند ممکن است نیاز به مراقبت های متفاوت پس از زایمان را نسبت به آنچه وجود دارد ، درک و تقاضا نکنند چرا که دیدگاه آن ها منطبق بر این اصل است که «آنچه هست» می تواند بهترین گزینه موجود باشد. در حالی که زنانی که قبلا نیز زایمان کرده اند توقعات و انتظارات بیشتری نسبت به تنوع و پوشش دهی و کفایت مراقبت ها ی حمایتی در ابعاد مختلف مورد نیاز آن ها را ذکر می کنند. این نشان می دهد که با تاکید و دانش به این موضوع که نیازهای زنان ممکن است بر اساس پاریته و تجارب قبلی آن ها تغییر کند نیاز به انعطاف پذیری در گزینه های مراقبت پس از زایمان وجود دارد (30).
به همین منظور و در راستای پاسخگویی مبتنی بر نیاز، در سرتاسر جهان الگوهای متفاوتی در ارایه خدمات مراقبتی پس از زایمان وجود دارد که در ادامه به برخی از آن ها اشاره می شود (16) برای مثال:
در استرالیا بیمارستان ها موظفند مراقبت های کافی پس از زایمان را برای زنان، نوزادان و خانواده های آنها بر اساس مسایل بالینی و نیازهای روانی – اجتماعی تضمین کنند. این شامل: حداقل یک ویزیت درخانه پس از زایمان یا نوعی دیگر از تماس و پیگیری برای همه زنان پس از ترخیص از بیمارستان؛ حداقل دو بار ویزیت در خانه پس از زایمان یا بیشتر، در صورت لزوم برای زنان و نوزادان با نیازهای خاص و متنوع، مانند زنان مبتلا به مسایل مربوط به سوء مصرف مواد، مهاجران تازه وارد، مادران جوان مجرد ، و زنان دارای معلولیت است. تعداد بیشتری از ویزیت های خانگی پس از زایمان برای زنان دارای عوارض ناشی از تولد در دوره بلافاصله پس از زایمان؛ و یا پس از ترخیص به ویژه در خصوص رسیدگی به نگرانی ها ی مربوط به خانواده و ارتباطات آن ها و سایر مراقبت های حمایتی لحاظ می شود (31).
همچنین در سیستم بهداشتی کشور سوئیس، کوتاه شدن مدت بستری در بیمارستان پس از زایمان با اجرای ویزیت های خانه پس از زایمان توسط ماماهای جامعه جبران شده است. زنان و خانواده ها می توانند در طول 56 روز اول پس از زایمان برای فرزند اول یا در شرایط پیچیده حداکثر 16 ویزیت از ماماهای جامعه خوداشتغال دریافت کنند و برای فرزند دوم و بعد از آن حداکثر 10 ویزیت دریافت کنند. میانگین تعداد بازدیدها در سال 2018 7.5 بوده است . در طول این بازدیدهای خانگی، ماماها سلامت جسمی زنان و نوزادان را بررسی می‌کنند و حمایت شخصی در مورد تغذیه با شیر مادر، مراقبت از نوزاد و سلامت روان ارائه می‌کنند (32).
در هلند، جایی که سیستمی از دستیاران مراقبت از مادران وجود دارد که تا هشت روز زنان را حمایت می کنند، ممکن است زنان در هلند با اطمبنان خاطر بیشتری تمایل داشته باشند که زودتر به خانه بروند. در حال حاضر در کشورهایی مانند ایران چنین سیستمی برای پیاده سازی وجود ندارد و نیازمند پیش بینی یک چهارچوب ارزیابی شده و نیز تغییر در نحوه تامین بودجه است (33)
موضوع طول اقامت غیر قابل انعطاف که با نیازهای زنان تطبیق داده نشده است موضوع مورد بحث مهمی است .ده سال پیش کمیسیون حسابرسی بریتانیا هشدار داد که مدت زمانی که زنان در بیمارستان می گذرانند نباید از قبل تعریف یا استاندارد شود، در عوض پیشنهاد می‌کند که سیاست‌ها انعطاف‌پذیر باشند و ضروری است که با زنان در مورد طول اقامت و ماهیت مراقبت از آنها مشورت شود (34). مهم است که هر حرکتی به سمت مدت اقامت کوتاهتر پس از زایمان بر حسب ارزیابی سلامت جسمی و روانی مادر و نوزاد، و رضایت مادر از مراقبت دریافت شده انجام پذیرد.
جدای از تحولات در عمل بالینی، کاهش میانگین مدت اقامت به عنوان راهی برای دستیابی به کارایی و هزینه اثربخشی تلقی می شود. در واقع، اقامت کوتاه‌تر، هزینه هر بیمار را کاهش می‌دهد. به نظر می رسد که ترخیص زودرس پس از زایمان در مطالعات کنترل شده با برنامه پیگیری پس از بیمارستان ایمن باشد. در همین راستا چندین کشور مانند فرانسه و انگلستان ظرفیت تخت خود را در خدمات پس از زایمان کاهش داده اند و در عوض به مراقبت های پس از ترخیص متمرکز شده اند. همچنین در کشور سوئیس بازدید های پس از زایمان به طور کامل در قالب طرح بیمه درمانی اجباری مندرج در قانون سوئیس LAMAL بازپرداخت می شوند، بنابراین خدمات نزدیک به خدمات جهانی را تضمین می کنند.. پرستاران و متخصصان مراقبت های اجتماعی ممکن است خدمات بیشتری را بعد از این دوره ارائه دهند. هنگامی که خانواده ها در پریشانی اجتماعی زندگی می کنند، از تنهایی، فقر، مسکن نامناسب یا به طور کلی فقدان حمایت رنج می برند، ماماهای جامعه تمایل دارند خدمات بیشتری را ارائه دهند. آنها با متخصصان دیگر مانند مددکاران اجتماعی تماس می گیرند، اما همچنین در مواقع ضروری اقدام مستقیم انجام می دهند (35).
اگر ترتیبات جایگزین برای ارایه مراقبت پس از زایمان معرفی شود، و برنامه های جامع مراقبت حمایتی با پوشش دهی همه جانبه زنان اتخاذ گردد می توان در خصوص رفع نیازهای زنان در دوره بحرانی پس از زایمان اطمینان اخذ کرد. و این نیاز مند انجام ارزیابی، بررسی دغدغه ها و ابعاد مختلف مشکلات زنان در دوره پس از زایمان از دیدگاه خود آنان و بررسی تاثیر چنین برنامه ای بر سلامتی زنان و نوزادان، همچنین اضطراب و اعتماد به نفس والدین؛ و تاثیرات اقتصادی برخدمات سلامت زنان و خانواده هایشان باشد (16).
از آن جا که برنامه ریزی دقیق و ارزیابی جدید برای اطمینان از کفایت مراقبت های پس از زایمان مورد نیاز است. باید رویکردهایی اتخاذ شود که مشکلاتی که پدران و مادران را در دستیابی به مراقبت های جامع پس از زایمان احاطه کرده است به طور کامل و با رویکرد مراقبت حمایتی جامع پوشش داده شود و نباید صرفا به مراقبت های محدود ارایه شده به زنان پس از ترخیص از بیمارستان بسنده نمود(16). این امر مستلزم انجام پژوهش های کیفی در راستای تبیین نیاز ها و مشکلات زوجین در این برهه زمانی است. از طرفی با توجه به اینکه تاکنون در ایران پژوهشی کیفی حول محور این موضوع انجام نشده و مطالعات قبلی صرفا به صورت کمی، کیفیت مراقبت های پس از زایمان را مورد سنجش قرار داده اند، لذا درراستای اولویت پژوهشی گروه مامایی و سلامت باروری دانشگاه علوم پزشکی تهران مبنی بر طراحی، اجرا،پایش و ارزشیابی مداخلات مرتبط با کاهش موربیدیته مادران و نوزادان پس از زایمان این مطالعه قصد دارد با یک رویکرد ترکیبی به تبیین نیازهای مراقبت حمایتی زوجین در دوره پس از زایمان پرداخته و یک مداخله مبتنی بر نیاز در راستای ارتقا خدمات سلامت در دوران پس از زایمان را طراحی و اجرا نماید. در صورت اثربخشی مداخله طراحی شده در اختیار سیاست گذاران و مدیران بهداشتی قرار داده خواهد شد تا در جهت ارتقا کیفیت مراقبت های پس از زایمان از آن استفاده نمایند.

**بررسی متون**

مطالعات کمی

1-درخشان پور و همکاران یک کارآزمایی بالینی با عنوان " تاثیر یک مداخله آموزشی روانی بر سلامت پس از زایمان" را بر روی 80 مادر باردار بستری در زایشگاه بیمارستان شهید صیاد شیرازی گرگان انجام دادند. آزمودنی ها به طور تصادفی به دو گروه مداخله (40 نفر) و کنترل (40 نفر) تقسیم شدند. آزمودنیهای گروه مداخله و همسرانشان 48 ساعت قبل از تاریخ تخمینی زایمان، آموزشهای بهداشت روانی پس از زایمان دریافت کردند. گروه کنترل هیچ آموزشی دریافت نکرد. داده ها با استفاده از پرسشنامه دموگرافیک و پرسشنامه سلامت عمومی-28 (GHQ-28) در ابتدا و در هفته دوم و چهارم پس از زایمان جمع آوری شد. داده ها با استفاده از نرم افزار SPSS (نسخه 18) در سطح معنی داری 05/0 تجزیه و تحلیل شد. میانگین سنی در گروه مداخله 6/5±3/26 سال و در گروه کنترل 1/5±9/27 سال بود. بین گروه ها از نظر سن، ترتیب بارداری، تعداد سقط، نوع زایمان و سابقه اعتیاد در ابتدا تفاوت معنی داری وجود نداشت. نمره کل GHQ-28 از ابتدا تا هفته چهارم پس از زایمان در هر دو گروه به طور معنی داری کاهش یافت (001/0=P). از نظر کاهش نمره کل GHQ-28 تفاوت معنی داری بین گروه ها وجود نداشت (05/0P>). همچنین تفاوت معنی داری بین گروه ها از نظر تغییر در نمرات خرده مقیاس های GHQ-28 در طول زمان وجود نداشت.
یافته های این مطالعه حاکی از آن است که مداخله آموزشی کوتاه مدت 24 تا 48 ساعت قبل از زایمان در بهبود سلامت روان در دوران پس از زایمان موثر نیست. بنابراین مداخلات آموزشی باید به مدت طولانی تری، چند هفته قبل یا بعد از زایمان در قالب مراقبت های حمایتی انجام شود. (36).
2-چن و همکاران در مطالعه ای با عنوان " اثرات مداخله گروهی حمایتی در زنان مضطرب پس از زایمان: یک مطالعه کنترل شده در تایوان" 60 زن مضطرب پس از زایمان را به طور تصادفی در دو گروه حمایت (30 نفر) و کنترل (30 نفر) قرار دادند. زنان منتسب به گروه حمایتی در چهار جلسه گروهی حمایتی شرکت کردند که شامل بحث هایی در مورد انتقال به مادری، مدیریت استرس پس از زایمان، مهارت های ارتباطی و برنامه ریزی زندگی بود. نمرات پرسشنامه افسردگی بک (BDI) و مقیاس استرس ادراک شده (PSS) در افرادی که در جلسات پشتیبانی شرکت کرده بودند ، کاهش یافت و در پایان چهارمین جلسه هفتگی، نمرات فهرست ارزیابی حمایت بینفردی (ISEL) نیز افزایش یافته بود. در مقابل، در گروه کنترل در این مدت تغییر معنی داری مشاهده نشد. این مطالعه کنترلشده شواهدی را ارائه میکند که نشان میدهد مشارکت در گروههای حمایتی برای زنان مضطرب پس از زایمان، مزایای روانی اجتماعی قابل سنجشی را فراهم میکند.
این مطالعه ازین نظر که به دغدغه های خانم ها در دوره پست پارتوم پرداخته و این مضامین را محور جلسات گروهی حمایتی قرار داده و به مقوله حمایت پس از زایمان پرداخته با مطالعه حاضر وجه اشتراکی دارد. لذا در بحث و تحلیل نتایج مورد استفاده قرار خواهد گرفت (37)
3-گلاوین وهمکاران یک کارآزمایی بالینی تحت عنوان " مشاوره حمایتی توسط پرستاران بهداشت عمومی برای زنان مبتلا به افسردگی پس از زایمان" انجام دادند که جمعیت مورد مطالعه شامل زنان در دوره پس از زایمان، ساکن در دو شهرداری نروژ بود که بین ژوئن 2005 و دسامبر 2006 یک فرزند زنده به دنیا آورده بودند. در مجموع 228 زن در این مطالعه وارد شدند پرستاران بهداشت عمومی در یکی از شهرداری ها در زمینه شناسایی افسردگی پس از زایمان و ارائه مشاوره حمایتی آموزش دیدند. پیش آزمون با استفاده از مقیاس افسردگی پس از زایمان ادینبورگ در 6 هفته پس از زایمان انجام شد. پس آزمون با استفاده از همان مقیاس در 3 و 6 ماه پس از زایمان انجام شد. نتایج نشان داد که نمره افسردگی در گروه آزمایش در مقایسه با گروه مقایسه در 3 و 6 ماه پس از زایمان به طور معنیداری کاهش یافت. محققان ذکر کردند که مشاوره حمایتی ارائه شده توسط پرستاران بهداشت عمومی یک روش درمانی موثر برای افسردگی پس از زایمان است. تحقیقات بیشتری برای مدیریت افسردگی پس از زایمان در مراقبتهای بهداشتی اولیه مورد نیاز است.
این مطالعه ازاین نظر که در مداخله خود به مشاوره حمایتی پرداخته که یکی از خلا های مراقبتی موجود در کشور ماست حایز اهمیت و درخور توجه است لذا در طراحی مداخله و بحث مورد استفاده و تحلیل قرار خاهد گرفت. این مطالعه ازین حیث که افراد دارای افسردگی پس از زایمان را به عنوان گروه هدف مطالعه در نظر گرفته است با مطالعه حاضر (افراد سالم) تفاوت دارد (38).
4-کوچاک و همکاران در مطالعه ای با عنوان " توسعه اپلیکیشن پشتیبانی تلفن همراه پس از زایمان و تاثیر اپلیکیشن بر علائم اضطراب و افسردگی مادران" بین ژوئیه 2017 و فوریه 2020 انجام دادند که نوع مطالعه کارازمایی کنترل شده پیش آزمون-پس آزمون بود و در آن کسانی که نوزادان سالمی داشتند گروه مورد مطالعه را تشکیل دادند. داده ها با استفاده از فرم اطلاعات دموگرافیک ، مقیاس وضعیت و تداوم اضطراب STAI و مقیاس افسردگی پس از زایمان ادینبورگ جمع آوری شد. برای تجزیه و تحلیل داده ها از آنالیز آنوای مختلط ، آزمون t در گروه های وابسته، آزمون t و آنالیز مجذور کای در گروه های مستقل استفاده شد. یافته ها حاکی از آن بود که اکثر مادرانی که از این نرم افزار استفاده می کردند، کودک خود را فقط با شیر مادر تغذیه می کردند و احساس کافی در مورد شیردهی داشتند. علائم افسردگی مادرانی که از برنامه پشتیبانی تلفن همراه پس از زایمان استفاده می کردند کمتر از مادران گروه کنترل بود. با این حال، مشخص شد که این برنامه به تنهایی برای کاهش سطح اضطراب و علائم افسردگی کافی نیست(P>0/05).
این مطالعه از حیث استفاده از شیوه های نوین پیگیری پس از ترخیص (اپلیکیشن تلفن همراه) درخور توجه می باشد لذا در طراحی مداخله مورد بحث و بررسی قرار خواهد گرفت (39).
5-باقرزاد و همکاران در یک کارآزمایی بالینی تحت عنوان " تاثیر مراقبت در منزل بر حمایت شوهر در طول دوران دوره پس از زایمان" 64 زن را که در بیمارستان های اصفهان زایمان کرده بودند به طور تصادفی در دو گروه مداخله و کنترل قرار دادند. برای جمع آوری داده ها از پرسشنامه محقق ساخته استفاده نموده و روایی و پایایی پرسش نامه را مورد ارزیابی قرار دادند. برای تجزیه و تحلیل داده ها از آمار توصیفی، آزمون تی مستقل، آزمون دقیق فیشر، کای اسکوئر و یو من ویتنی استفاده نمودند. سطح معنی داری کمتر از 05/0 در نظر گرفته شد. در بخش نتایج محققان ذکر کردند که پس از دریافت مراقبت در منزل، حمایت شوهر در گروه مداخله با گروه کنترل تفاوت معناداری داشت( p=0/001). همچنین بین گروه مداخله و کنترل در زمینه های اعتماد به همسر، اعتماد به او، توجه به نیاز های مراقبتی او، حمایت مالی، تلاش برای رفع مشکلات همسر، خریدن هدیه، کمک به مراقبت از فرزند تفاوت معناداری وجود داشت(P<0/05). طبق نتایج این پژوهش برنامه مراقبت در منزل به طور مطلوبی می تواند حمایت شوهر را افزایش دهد که منجر به یک دوره ایمن پس از زایمان گردد. همچنین ارائه خدمات خانگی نیازمند برنامه ریزی دقیق و مناسب و مدیریت توسط ماماها است.
این مطالعه از این نظر که به ابعاد حمایت اجتماعی و مراقبت توسط همسر به عنوان مداخله پرداخته درخور توجه و ارزشمند است و به شکل متفاوتی از سایر مطالعات بعد مراقبت در منزل توسط اعضای خانواده را هدف قرار داده که در طراحی مداخله مطالعه حاضر مورد استفاده قرار خواهد گرفت (40).
-6فنویک و همکاران( 2010) در مطالعه ای با عنوان" برداشت زنان استرالیای غربی از سبک و کیفیت مراقبت های پس از زایمان مامایی در بیمارستان و در خانه" از یک پیمایش مقطعی بر مبنای خودگزارش دهی برای توصیف ابعاد مختلف حمایت اطلاعاتی، عملی و عاطفی ارایه شده توسط ماماها در دوره پس از زایمان استفاده کردند. پرسشنامه طراحی شده به طور ویژه برای جمعیت تحت مطالعه، توسط 2699 زن در 8 هفته اول پس از زایمان تکمیل شد. . داده ها با استفاده از آمار توصیفی، آزمون t و مجذور کای تجزیه و تحلیل شدند. نتایج مطالعه نشان داد که زنان از بیشتر جنبه های مربوط به مراقبت مامایی مربوط به ارایه توصیه ها و کمک های عملی در رابطه با مراقبت از نوزاد و و بهبود جسمانی آن ها راضی بودند. حوزه هایی که امتیاز مثبت کمتری دریافت کردند مربوط به ارائه مشاوره مداوم، در دسترس بودن ماماها، مراقبت عاطفی، ایمن سازی و پیشگیری از بارداری، ارایه اطلاعات در مورد نیازهای سلامت مادر بود. محققان اظهار داشتند که مادران نخست زا به طور کلی به سبک و کیفیت مراقبت ها نسبت به مادران چندزا امتیاز کمتری داده اند و در میان زنانی که مراقبت های ماماها را در رتبه کمتر مطلوب قرار می دهند گرایشی به سمت مراقبت های بیمارستان های خصوصی وجود دارد. در پژوهش آنان مراقبت های مامایی در منزل بسیار مثبت و به طور قابل توجهی بهتر از مراقبت های بیمارستانی ارزیابی شد. اگرچه اکثریت زنان در این مطالعه از مولفه های مراقبت بدنی و ارایه اطلاعات و کمک در مورد تغذیه و خواب و اسکان نوزاد در کوتاه مدت راضی بودند، رضایت کمتری از مراقبت عاطفی و و آمادگی برای زندگی در منزل به همراه یک نوزاد جدید و تطابق با فرزند پروری وجود داشته است (41)
این مطالعه به درک ما از تجربیات و انتظارات زنان در اوایل دوران پس از زایمان می افزاید و اطلاعاتی را ارایه می دهد که برمبنای آن می توان در خدمات و مراقبت های پس از زایمان پیشرفت حاصل نمود که از نقاط قوت این مطالعه است. حجم بالای نمونه که زمینه را برای تعمیم پذیری یافته ها فراهم می کند، بررسی مولفه های مختلف و متعدد نیاز های مراقبتی زنان (مراقبت فردی، مراقبت از نوزاد، نیاز های جسمانی و نیاز های عاطفی) و تنوع نمونه گیری از حبث رتبه بارداری که زمینه را برای آگاهی از تفاوت برداشت زنان نخست زا و چندزا از جنبه های مختلف مراقبت ها فراهم می کند از دیگر نقاط قوت مطالعه است. در این مطالعه محققان از رویکرد کمی برای بررسی کیفیت مراقبت های پس از زایمان از دیدگاه زنان مورد بررسی استفاده کرده اند درحالی که مطالعاتی از این قبیل بهتر است به صورت کیفی به ارزیابی افراد تحت مطالعه خود بپردازد. از این مطالعه در بیان مساله و ضرورت تحقیق استفاده شده و نتایج آن در طراحی سوالات مصاحبه و تحلیل و تفسیر نتایج بکار برده خواهد شد.
7-سیمبر و همکاران(1384) در مطالعه ای توصیفی باعنوان " بررسی کیفیت مراقبت های پس از زایمان در بیمارستان های تابعه ی دانشگاه علوم پزشکی و خدمات بهداشتی درمانی شهیدبهشتی" به بررسی 60 زن مراجعه کننده به مراکز بهداشتی درمانی پس از زایمان طبیعی پرداختند. میانگین سنی نمونه ها 3/24 سال بود و حاملگی و تعداد فرزندان زنده به ترتیب در 78 درصد و 83 درصد نمونه ها یک الی دو فرزند بود که اکثریت آن ها هیچ موردی از مرگ و میر فرزند و سقط جنین را نداشتند. محققان اظهار داشتند که کیفیت ارایه مراقبت ها در بخش پس از زایمان در اکثریت موارد ضعیف بوده است و با توجه به اهمیت این مراقبت ها در پیشگیری از عوارض دوره پس از زایمان، انجام بررسی ها و تحقیقات بیش تری در رابطه با علل کیفیت پایین این مراقبت ها توصیه کرده اند (12).
مسجل ننمودن متغیرهای مورد بررسی در راستای سنجش کیفیت مراقبت های پس از زایمان از جمله نقاط ضعف این پژوهش است. (محققان به این موضوع که کیفیت مراقبت های پس از زایمان را با چه آیتم ها و ملاک هایی مورد سنجش قرار داده اند اشاره ای ننموده اند). ازدیگر نقاط ضعف این مطالعه می توان به این مساله اشاره کرد که کیفیت مراقبت های پس از زایمان از طریق مطالعه کمی سنجیده شده بود در حالی که تعیین ابعاد کیفیت مراقبت ها و چالش های آن نیازمند در نظر گرفتن دیدگاه ،تجارب ،هیجانات و احساسات فرد و تعاملات اجتماعی است که باید از طریق مطالعه کیفی به آن دست یافت. از این مطالعه در بیان مساله و ضرورت تحقیق استفاده شده و نتایج آن در تحلیل و تفسیر نتایج بکار برده خواهد شد.
8-قبادی و همکاران( 1397) در مطالعه ای توصیفی-تحلیلی با عنوان " بررسی میزان رضایت مندی از تجربه زایمان طبیعی و عوامل مرتبط با آن در زنان شهر رشت" به بررسی 126 زن بستری در بخش بعد از زایمان طبیعی پرداختند. نمونه ها به روش در دسترس انتخاب شده بود و ابزار گرداوری داده ها، پرسشنامه ای محقق ساخته و شامل سه بخش عوامل انسانی، عوامل محیطی و اقدامات مرتبط با فرآیند لیبر و زایمان بود. داده های پژوهش با استفاده از آزمون های همبستگی اسپیرمن، پیرسون و تی تست تجزیه و تحلیل شده بودند. بر اساس یافته های گزارش شده توسط محققان میانگین نمره رضایتمندی مادران از تجربه زایمان طبیعی، 03/59 و در حد متوسط بود. بیشترین میزان رضایتمندی از عوامل محیطی گزارش شده بود(68 درصد).رضایتمندی مادران از دسترسی به امکانات لیبر و دریافت اطلاعات کافی پیرامون مراحل لیبر و زایمان، تاثیرات یکسانی در رضایتمندی مادران از تجربه زایمان داشتند. برخورد محترمانه پرسنل لیبر، مهمترین عامل در جلب رضایتمندی مادران از تجربه زایمان بود. درواقع کیفیت روابط بین زنان و مراقبین از عوامل مهم تعیین کننده است. پیشبرد مراقبت حین زایمان به سوی مراقبت های مامایی زن محور و ایجاد روابط مناسب با زنان از موثرترین اقدامات جهت ارتقا کیفیت مراقبت های مامایی و بهبود رضایت زنان از زایمان طبیعی است (42).
بررسی ابعاد مختلف دخیل در رضایتمندی زنان از تجربه زایمان طبیعی به تفکیک عوامل محیطی، انسانی و پروسه ی زایمان از نقاط قوت این مطالعه محسوب می شود اگرچه استفاده از نمونه های در دسترس تعمیم پذیری یافته ها را کاهش داده و بهتر است در مطالعاتی از این قبیل از نمونه های با حداکثر تنوع و نیز مطالعات کیفی( به جای بررسی کمی) استفاده نمود. از این مطالعه در بیان مساله و ضرورت تحقیق استفاده شده و نتایج آن در طراحی سوالات مصاحبه و تحلیل و تفسیر نتایج بکار برده خواهد شد.


مطالعات کیفی
9- فورستر و همکاران(2008) در پژوهشی کیفی با عنوان" دوره اولیه پس از تولد: بررسی دیدگاه ها، انتظارات زنان و تجربیات مراقبت با استفاده از گروه های متمرکز " که در مناطق شهری و روستایی ویکتوریا، استرالیا انجام شده بود با 52 نفر در هشت گروه متمرکز مصاحبه انجام دادند. شرکت کنندگان شامل 8 زن باردار و 42 زن در دوره پس از زایمان و دو نفر از همسران آنان بودند. مصاحبه ها جهت بررسی تجارب و انتظارات شرکت کنندگان از مراقبت های پس از زایمان در بیمارستان و منزل با تاکید بر طول مدت بستری در بیمارستان، حمایت حرفه ای و اجتماعی، و تداوم مراقبت ها بود. تم های کلی استخراج شده از مصاحبه ها شامل اضطراب/ترس، و گذار به مادری و فرزندپروری بود. محققان اظهار داشتند که نیاز های مادران نخست زا با نیاز های مادرانی که قبلا تجربه مادری را داشتند متفاوت بود و زنان در این مطالعه به طور کلی نگران ایمنی نوزاد جدید خود بودند، و به عنوان یک مادر جدید نسبت به توانایی خود در مراقبت از نوزاد خود اطمینان نداشتند. آنان در نتایج پژوهش خود تاکید کردند که بر اساس دیدگاه های موجود حضور فیزیکی و در دسترس بودن پشتیبانی حرفه ای به کاهش این نگرانی ها کمک کرده است، و این به ویژه در مورد زنانی که اولین نوزاد را به دنیا می آورند، صدق می کند و ارایه دهندگان مراقبت باید ازین مسایل آگاه باشند . بسیار مهم است که نگرانی ها و نیاز های زنان در هنگام برنامه ریزی برای ارایه خدمات لحاظ شود. اگر اضطراب برای فرزند پروری جدید، دیدگاه غالب است ارائه دهندگان مراقبت باید این نیاز را بشناسند و اطمینان حاصل کنند که مراقبت ها به صورت فردی شده و جهت رسیدگی به نگرانی های اختصاصی هر زن/خانواده ارایه می شود (16).
در نقد مطالعه فوق می توان به این نکته اشاره نمود که هدف پژوهشگران بررسی تجارب و انتظارات افراد از مراقبت های پس از زایمان است در حالی که تعدادی از نمونه های تحت مطالعه افراد باردار هستند . لذا با توجه به اینکه افرادی که در دوره پس از زایمان بسر میبرند می توانند درک بهتری از انتظارات خود در این دوره داشته باشند پیشنهاد می شود که در مطالعاتی از این قبیل از افراد واقع در دوره پس از زایمان استفاده کرد. به علاوه جهت رسیدن به دیدگاه صحیح تر و جامع تر در خصوص نظرات پدران در صورت افزایش تعداد نمونه های مرد، نتایج مطالعه، قابل استنادتر خواهد نمود. تفکیک نتایج بررسی از دیدگاه زنان نخست زا و زنان دارای تجربه قبلی بارداری از نقاط قوت این مطالعه محسوب می شود. این مطالعه با توجه به کیفی بودن متدلوژی و با توجه به مشارکت پدران به عنوان بخشی از شرکت کنندگان در مطالعه با مطالعه حاضر وجه اشتراک دارد. از این مطالعه در بیان مساله و ضرورت تحقیق استفاده شده و با توجه به رویکرد کیفی ازین مطالعه می توان در بخش معیارهای ورود و نتایج آن در طراحی سوالات مصاحبه و تحلیل و تفسیر نتایج استفاده نمود
10- مک لیش و همکاران (2020) پژوهش خود را با عنوان " مطالعه ی کیفی تجربیات مادران نخست زا از حمایت اجتماعی پس از زایمان متخصصان بهداشت در انگلستان" به شیوه پدیدارشناسی با استفاده از مصاحبه های عمیق و نیمه ساختاریافته با 32 مادر با پیشینه های مختلف انجام دادند. با استفاده از تحلیل موضوعی استقرایی به چهار مضمون کلی در ابعاد مختلف حمایت اجتماعی(عاطفی، ارزیابی، اطلاعاتی و عملی) دست یافتند. در نتایج پژوهش آنان نه موضوع مرتبط با حمایت اجتماعی وجود داشت که که قوی ترین نگاشت مربوط به ابعاد ارزیابی و پشتیبانی اطلاعاتی بود.
تم های مرتبط با بعد ارزیابی شامل تحسین و اعتبار، انتقاد و تضعیف، و ایجاد احساس ناتوانی و تم های مرتبط با بعد پشتیبانی اطلاعاتی شامل اقدامات پیشگیرانه، آموزش علائم خطر و وضعیت های غیرنرمال، رفع سردرگمی در مورد مراقبت های پس از زایمان بود. بعد حمایت عاطفی شامل احساس درک و شنیده شدن، مراقبت غیر شخصی(عدم توجه به فردیت در مراقبت ها) و نادیده گرفته شدن و بعد حمایت عملی شامل توانمندسازی شرکای جنسی برای ارایه حمایت بود. محققان در پایان اظهار داشتند که متخصصین سلامت می توانند در دوره پس از زایمان نقش مهمی در کمک به مادران نخست زا با ارایه مناسب و شخصی سازی شده مراقبت ها، ایجاد اعتماد به نفس، انجام ارزیابی های مناسب فردی، حمایت اجتماعی عاطفی و اطلاعاتی در کنار مراقبت بالینی ایفا کنند و این موضوع نیازمند آموزش و پشتیبانی حرفه ایست تا اطمینان حاصل شود که کلیه متخصصین سلامت توانایی ارایه حمایت اجتماعی را در کنار سایر خدمات خود دارا هستند (43).
نیاز های حمایتی زنان در دوره پس از زایمان دارای ابعاد مختلف و گسترده ای است که این پژوهش تنها به بررسی بعد حمایت اجتماعی پرداخته که از نقاط ضعف این مطالعه محسوب می شود. لذا این مطالعه با بررسی یکی از ابعاد نیازهای حمایتی زنان پس از زایمان با مطالعه ما وجه اشتراک دارد. بنابراین در مطالعه پیش رو سعی خواهد شد با بررسی ابعاد مختلف نیازهای حمایتی زوجین نتایج جامع تری را کسب کنیم. همچنین با توجه به نتایج ذکر شده در خصوص مادران نخست زا ، بررسی جداگانه مادران نخست زا و مولتی پار در مطالعه پیش رو می تواند تفاوت نیاز های حمایتی مدنظر این دو گروه از زنان را به صورت نتایج ارزشمندی برای ما مسجل سازد. از نتایج این مطالعه در طراحی سوالات پژوهش و تحلیل نتایج استفاده خواهد شد.
11- پولاک و همکاران( 2005) در پژوهشی کیفی با عنوان " پدران با اولین تجربه فرزندآوری و عوامل استرس زا در دوره پس از زایمان" به بررسی یک نمونه در دسترس متشکل از 19 شرکت کننده شامل پدرانی که برای اولین بار در سنین 18 تا 45 سالگی فرزندآوری را تجربه می کردند پرداختند. داده ها از طریق مصاحبه تلفنی گرداوری شدند. جهت سنجش عوامل استرس زا از نظر سنجی و مصاحبه با پدران و نیز جهت بررسی ماهیت و شدت عوامل استرس زا از شاخص استرس های روزمره(ESI) استفاده شد . نتایج نشان داد که عواملی مانند احساس نداشتن زمان کافی برای مسئولیت های زیاد، مسایل مالی و نگرانی در مورد سلامت کودک و سایر اعضای خانواده منبع استرس افراد بود. محققان گزارش کردند که به استثنای تفاوت های جزئی در رتبه بندی، استرس های روزمره در بین پدرانی که برای اولین بارفرزندآوری را تجربه می کنند، شباهت زیادی به عوامل استرس زای گزارش شده قبلی از مادران نخست زا دارد. اگرچه حجم نمونه کم بود و نتایج ممکن است غیر قابل تعمیم باشد این یافته ها نشان می دهد که تلاش های آموزشی توسط متخصصان مراقبت های بهداشتی می تواند برای پدران و مادران در دوران بارداری و پس از زایمان مفید باشد (21).
محققان جهت مطالعه از نمونه های در دسترس استفاده نموده و آن ها را صرفا به مردانی که برای اولین بار پدر شده اند منحصر نموده اند. در حالی که استفاده از رویکرد نمونه گیری با حداکثر تنوع قدرت تعمیم یافته ها را افزایش داده و نیز استفاده از نظرات پدرانی که قبلا سابقه فرزندآوری داشته اند می تواند اطلاعات مفیدتری ضمن مقایسه نظرات این افراد با پدران دارای نخستین تجربه فرزند آوری بدست دهد. از نقاط قوت مطالعه مقایسه نتایج توسط پژوهشگران با نتایج حاصل از مطالعات مشابه در مادران نخست زا است که اطلاعات مفید و نگرش جامعی را در اختیار خواننده قرار می دهد. این مطالعه از این نظر که پدران را مورد بررسی قرار داده است مشابه مطالعه حاضر است، لذا در بررسی نیاز های حمایتی پدران در دوره پس از زایمان میتوان از سوالات مورد استفاده این پژوهشگران در مصاحبه الگو گرفت و نتایج مطالعه آنان را در قسمت بحث و نتیجه گیری با مطالعه پیش رو مورد مقایسه قرار داد. از این مطالعه در طرح پیشنهادی تحقیق در نگارش ضرورت تحقیق و بیان مساله استفاده شده است
12- آدامز و همکاران در یک مطالعه کیفی درسال 2023 با عنوان "ارزیابی نیاز های مراقبت پس از زایمان: درک زنان از مراقبت های پس از زایمان، موانع و نیاز های آموزشی" به هشت بحث گروهی متمرکز در میان 54 زن در دوره پس از زایمان در چهار مرکزبهداشتی درمنطقه ساگناریگو تاماله در غنا پرداختند پس ازرونویسی و ترجمه داده های ضبط شده و تجزیه و تحلیل موضوعی شش تم اصلی استخراج شد که شامل: مراقبت های بعد اززایمان متمرکز بر نوزاد، اعمال پس از زایمان، آگاهی ناکافی از علایم خطر پس از زایمان، موانع دسترسی به مراقبت های پس از زایمان، تجارب سلامت روان ضعیف، نیاز به آموزش پس از زایمان بودند.به گفته محققان مراقبت پس از زایمان برای زنان در این مطالعه در درجه اول به عنوان مراقبت از نوزاد پس از زایمان و کسب اطلاعات کلیدی در مورد مراقبت از سلامت جسمی و روانی برای مادر تلقی شد و عدم دسترسی به اطلاعات این حوزه ها می تواند منجر به تطابق ضعیف با دوره پس از زایمان شود و فقدان دانش در مورد علائم خطر برای علل شایع عوارض و مرگ و میردر دوره پس از زایمان مقوله ای بحرانیست. بنابراین تحقیقات آینده نیاز به درک چگونگی انتقال اطلاعات مهم در مورد سلامت روانی و جسمی پس از زایمان برای محافظت بهتر از مادران دارد (44). این مطالعه با توجه به هدف کلی ارزیابی نیازها و درک زنان از مراقبت های پس از زایمان، همسو با بخش کیفی مطالعه حاضر است لذا در طراحی سوالات پژوهش و در بخش بحث و نتیجه گیری مورد مقایسه و تحلیل قرار خواهد گرفت و بخش طراحی مداخله و اجرا و ارزشیابی مطالعه حاضر در راستای پیشنهاد پژوهشی محققین این مطالعه است.


مطالعات مروری
13- لیتگو و همکاران(2021) در مطالعه ای با عنوان" چگونه تجارب و ادراکات زنان از مراقبت، بر جذب و استفاده از مراقبت های پس از زایمان تاثیر می گذارد، یک مطالعه ی مروری سیستماتیک کیفی در در سراسر جنوب صحرای آفریقا" در فاصله سال های 2009-2019 به بررسی سیتماتیک شواهد کیفی پرداختند. مطالعه مروری آنان کلیه متون کیفی به زبان انگلیسی که" ادراکات و تجربیات زنان را از کیفیت مراقبت هایی که پس از زایمان(محترمانه و غیرمحترمانه) دریافت کرده اند و اینکه چگونه این موضوع بر تصمیمات آن ها برای دسترسی به مراقبت های پس از زایمان تاثیر می گذارد " را در برمی گرفت(شامل می شد). تحلیل موضوعی برای استخراج مضامین فرعی و اصلی انجام شده بود. پانزده مطالعه با داده های حاصل از مصاحبه با 985 زن در هشت کشور وارد مطالعه شدند. در این مطالعات مراقبت محترمانه به صورت مهربانی ارایه دهندگان خدمت، حمایت کننده بودن و توجه به نیاز های زنان در نظر گرفته می شد و توصیف مراقبت های غیرمحترمانه شامل آزارکلامی/فیزیکی و عدم تعادل قدرت بین زنان و ارایه دهندگان خدمت(اعمال قدرت از سوی ارایه دهندگان خدمت) بود. در نتایج مطالعه محققان اذعان داشتند که زنان ترجیح می دهند خدمات مراقبت های بهداشتی را در جایی که ارایه دهندگان مراقبت های بهداشتی با احترام ارتباط برقرار می کنند دریافت کنند و شواهد حاکی از آن بود که زنان آسیب پذیر( نوجوانان، زنان با وضعیت اجتماعی-اقتصادی ضعیف، و زنان HIV مثبت)احتمال بیش تری دارد که مراقبت های غیرمحترمانه دریافت کنند. این بررسی سیستماتیک توضیح میدهد که چگونه جنبههای مراقبتهای مادرانه محترمانه و بیاحترامی بر ادراک و تجربیات زنان و تصمیمگیریها برای دسترسی به خدمات مراقبت پس از زایمان تاثیر میگذارد. نیاز به تمرکز مجدد برای اولویتبندی مراقبتهای محترمانه زایمان و ارایه پایدار مراقبتهای پس از زایمان با کیفیت خوب برای همه زنان و نوزادانشان به گونه ای که انتظارات و نیازهای سلامتی آنها را برآورده کند، وجود دارد (24).
علی رغم گنجاندن مطالعاتی از کشورهای مختلف در این مطالعه مروری، که تعمیم پذیری یافته ها را در نتیجه تنوع نمونه های در مجموع، افزایش می دهد، تعداد محدود مطالعات مورد بررسی از جمله نقدهاییست که می توان به مطالعه وارد نمود. با توجه به نتایج ذکر شده در خصوص زنان آسیب پذیر و تاکید بر آن ها که از نقاط قوت این مطالعه محسوب می شود، جهت افزایش تنوع در نمونه گیری و افزایش تعمیم پذیری و گستردگی طیف یافته های پژوهش در مطالعه حاضر سعی خواهد شد که این گروه های هدف از زنان نیز در مطالعه وارد شوند. لذا با درنظر گرفتن متغیرهای جمعیت شناختی مطالعات مورد بررسی در این مرور سیستماتیک ، سعی در حداکثر تنوع در نمونه گیری خواهد شد.همچنین نتایج این پژوهش در تحلیل و تفسیر نتایج نقش خواهند داشت
14- وارن و همکاران ( 2022) در پژوهشی با عنوان " ادراکات و تجربیات پدران از حمایت به عنوان یک شریک والدی در طول دوره پری ناتال" به بررسی و مرور سیتماتیک مطالعات کیفی و ترکیبی پرداختند. جست و جوی سیتماتیک از طریق پایگاه های داده مختلف صورت پذیرفت . به طور کلی 23 مقاله وارد مطالعه شدند. بر اساس نتایج مطالعات مردان مایل به حمایت شدن و طی کردن مراسم گذر جهت تطابق با گذار به دوره والدی بودند. این روند انتقالی به مردان جهت بیان تعهد خود برای ایفای نقش به عنوان یک پدر و آمادگی برای نقش آفرینی به عنوان الگو برای فرزندان آینده کمک می کند. پدران نیازمند حمایت همه جانبه در همه سطوح اکوسیستم شامل سیاست گذاری ها، تغییرات اجتماعی- فرهنگی، و نیز تغییرات محل کار و درک و حمایت از جانب همکاران، خانواده، همسالان و بالاخص ارایه دهندگان خدمات بهداشتی هستند. درواقع توسعه مشارکت والدین، نیازمند رویکردهای متعهدانه در همه سطوح خرد، میانی و کلان است (23).
با توجه به این که پژوهشگران در مطالعه مروری خود علاوه بر مطالعات کیفی، مطالعات ترکیبی را نیز مورد بررسی قرار داده اند می توان آن را از نقات قوت این پژوهش دانست. اگرچه در این مطالعه به بررسی ادراک پدران از حمایت پرداخته شده است اما در نتایج به این نکته که چه مقوله هایی از دیدگاه پدران حمایت تلقی می شود اشاره ای نشده که از خلا های ذکر نتایج در مرور مطالعات مذکور است. نتایج این بررسی در خصوص ادراک پدران از "حمایت" همسو با متغیر مورد بررسی(نیاز های حمایتی) در پژوهش پیش رو است. لذا در طراحی سوالات پژوهش و در بخش بحث و نتیجه گیری مورد مقایسه و تحلیل قرار خواهد گرفت
مطالعات مذکور به درک ما از تجربیات و انتظارات زنان در اوایل دوران پس از زایمان می افزاید و اطلاعاتی را ارایه می دهد که برمبنای آن می توان در خدمات و مراقبت های پس از زایمان پیشرفت حاصل نمود.
نتایج مطالعات حاکی از آن بود که زنان بیش تر از جنبه های مراقبت مامایی مربوط به ارایه توصیه ها و کمک های عملی در رابطه با مراقبت از نوزاد و و بهبود جسمانی آن ها راضی بودند و در واقع ابعاد جسمانی سلامت مادر و مراقبت نوزاد عمده تمرکز مراقبت های پس از زایمان را دریافت می دارند ودر حالی که نیاز های حمایتی زنان در دوره پس از زایمان دارای ابعاد مختلف و گسترده ای است و مراقبین به حوزه های مربوط به مراقبت روانی اجتماعی، ارائه مشاوره مداوم، مراقبت عاطفی، ارایه اطلاعات در مورد نیازهای سلامت مادر (از قبیل ایمن سازی و پیشگیری از بارداری)زمان و توجه کمتری را اختصاص می دهند.
به علاوه مطالعات انجام شده ازین حیث که تنها به برخی از ابعاد نیازهای حمایتی زنان پس از زایمان می پردازند و یا صرفا با مردان یا زنانی که نخستین بار پدر شدن را تجربه کرده اند وارد مطالعه خود نموده اند خلا هایی دارند که در مطالعه پیش رو در صدد مرتفع ساختن آن ها هستیم زیرا که ما نیازمند بررسی ابعاد مختلف نیازهای حمایتی زوجین برای کسب نتایج جامع تر هستیم و به علاوه استفاده از نظرات پدران یا مادرانی که قبلا سابقه فرزندآوری داشته اند می تواند اطلاعات مفیدتری ضمن مقایسه نظرات این افراد بدست دهد.
لذا در مطالعه پیش رو سعی بر این خواهد بود که با حداکثر تنوع در نمونه ها از حیث سن، رتبه فرزندآوری و... و وارد نمودن پدران در مطالعه در کنار همسران آنان (به صورت زوجی) جهت بررسی این مهم که چه مقوله هایی از دیدگاه آنان حمایت تلقی می شود و در مطالعات قبلی به آن پرداخته نشده و نیز پرداختن به گروه های آسیب پذیر زنان به بررسی ابعاد مختلف دخیل در رضایتمندی زوجین از تجربه زایمان طبیعی به تفکیک عوامل محیطی، انسانی و پروسه ی زایمان بپردازیم. لازم به ذکر است که در مطالعات داخلی به مقوله نیاز های مراقبت های حمایتی در بخش های پس از زایمان و مراکز بهداشتی به صورت کیفی پرداخته نشده و مطالعات موجود در این زمینه کمی هستند. با توجه به اینکه تاکنون در ایران پژوهشی کیفی حول محور این موضوع انجام نشده و مطالعات قبلی صرفا به صورت کمی، کیفیت مراقبت های پس از زایمان را مورد سنجش قرار داده اند این مطالعه قصد دارد با یک رویکرد ترکیبی به تبیین نیازهای مراقبت حمایتی زوجین در دوره پس از زایمان پرداخته و یک مداخله مبتنی بر نیاز در راستای ارتقا خدمات سلامت در دوران پس از زایمان را طراحی و اجرا نماید.

**تعریف واژه ها**

نیاز
تعریف نظری: نیاز به موقعیتی دلالت دارد که در آن وضعیت موجود یا حاضر با وضعیت مطلوب فاصله دارد. وضعیت مطلوب در برگیرنده ایده¬ آل¬ها، هنجارها، ترجیحات، انتظارات و ادراکات مختلف درباره آنچه که باید باشد است (45).
تعریف عملی: در این مطالعه مقصود، نیازهای مراقبت حمایتی زوجین در دوره پس از زایمان است که از طریق مصاحبه¬های عمیق نیمه ساختار یافته استخراج می¬گردد.

مراقبت حمایتی
تعریف نظری: نوعی از مراقبت است که به جنبه های مختلف اطلاعاتی، جسمی، عاطفی ، روانشناختی نیاز های افراد در شرایط خارج از معمول و ویژه زندگی می پردازد. هدف اولیه مراقبت های حمایتی بهبود کیفیت زندگی افراد است . ابعاد کلیدی مراقبت حمایتی شامل مدیریت درد و علایم، حمایت عاطفی و روان شناختی، بهبود ارتباطات ، توانایی تصمیم گیری، رسیدگی به نیاز های عملی( نگرانی های مالی، دسترسی به منابع و ....)، احترام به کرامت و خودمختاری، و ارایه مراقبت های هماهنگ و یکپارچه است (5).
تعریف عملی: در این پژوهش مقصود از مراقبت حمایتی، مراقبت ارایه شده در جنبه های مختلف اطلاعاتی، جسمی، روانشناختی، عاطفی در دوره پس از زایمان به مادران و همسران آن ها از سوی کادر بهداشت و درمان در بخش های بستری و مراکز سرپایی است و منطبق بر تعریف نظری است. این مفهوم از طریق مصاحبه های عمیق و نیمه ساختاریافته استخراج خواهد شد.
دوره پس از زایمان:
تعریف نظری:
دوره پس از زایمان که از آن به عنوان دوره نفاس یا تریمستر چهارم یاد نیز یاد می شود، به هفته های اول پس از تولد اطلاق می شود. هنگامی که تغییرات فیزیولوژیک و آناتومیک مرتبط با بارداری به وضعیت غیرباردار برمی گردد (46).
تعریف عملی: در این پژوهش ، تعریف عملی منطبق بر تعریف نظری بوده وبازه زمانی جهت نمونه گیری 8 هفته پس از زایمان را شامل می شود.

**سوالات پژوهشی و فرضیات**

فرضیات یا سوالات پژوهش (باتوجه به اهداف طرح) :
سوالات پژوهش:
1-تبیین زوجین ایرانی از نیاز مراقبت حمایتی در دوره پس از زایمان چیست؟
2-تبیین ارایه دهندگان خدمات سلامت از نیاز مراقبت حمایتی در دوره پس از زایمان چیست؟
3-تبیین صاحب نظران (مطلعین کلیدی، مدیران و سیاست گذاران) از نیاز مراقبت حمایتی در دوره پس از زایمان چیست؟
4-مهم¬ترین نیازهای مراقبت حمایتی زوجین در دوره پس از زایمان بر اساس پنل صاحب نظران کدامند؟
5-بر اساس مرور متون و پانل صاحب نظران چه مداخله ای در زمینه نیاز های مراقبت حمایتی زوجین در دوره پس از زایمان مناسب است؟
6-تسهیل گر ها و موانع مراقبت حمایتی زوجین در دوره پس از زایمان کدامند؟
7- آیا مداخله طراحی شده بر رفع نیاز های مراقبتی زوجین موثر است؟

فرضیات پژوهش:
اجرای مداخله بر رفع نیاز زوجین در خصوص حمایت ها و مراقبت های پس از زایمان موثر است.

**فهرست منابع مورد استفاده**

1. Infurna MR, Bevacqua E, Costanzo G, Falgares G, Giannone F. Psychosocial Risk Factors and Psychopathological Outcomes: Preliminary Findings in Italian Pregnant Women. Women. 2023;3(1):121-31.
2. Austin M-P, Committee MSPSA. Marcé International Society position statement on psychosocial assessment and depression screening in perinatal women. Best Practice & Research Clinical Obstetrics & Gynaecology. 2014;28(1):179-87.
3. Asadi M, Noroozi M, Alavi M. Identifying women’s needs to adjust to postpartum changes: a qualitative study in Iran. BMC Pregnancy and Childbirth. 2022;22(1):115.
4. Slomian J, Emonts P, Vigneron L, Acconcia A, Glowacz F, Reginster J-Y, et al. Identifying maternal needs following childbirth: A qualitative study among mothers, fathers and professionals. BMC pregnancy and childbirth. 2017;17(1):1-13.
5. Chambers EJ, Brown E, Germain M. Supportive care for the renal patient: OUP Oxford; 2010.
6. El-Khoury F, Sutter-Dallay A-L, Panico L, Charles M-A, Azria E, Van der Waerden J, et al. Women’s mental health in the perinatal period according to migrant status: the French representative ELFE birth cohort. The European Journal of Public Health. 2018;28(3):458-63.
7. Simsek A, Balkan E, Caliskan E. Determination of mothers' thoughts and adaptation behaviors regarding the infant: A descriptive study. Pediatrics & Neonatology. 2022;63(3):276-82.
8. McKellar LV, Pincombe JI, Henderson AM. Insights from Australian parents into educational experiences in the early postnatal period. Midwifery. 2006;22(4):356-64.
9. Rudman A, El-Khouri B, Waldenström U. Evaluating multi-dimensional aspects of postnatal hospital care. Midwifery. 2008;24(4):425-41.
10. Health WHOR. Medical eligibility criteria for contraceptive use: World Health Organization; 2010.
11. سیمبر م, غفاری ف, ترک‌زهرانی ش, علوی‌مجد ح. کیفیت ارایه مراقبت های مامایی به زنان بستری در بخش زایمان بیمارستان های منتخب، دانشگاه علوم پزشکی کردستان. فصلنامه پایش. 1388:11.
12. سیمبر م, علی‌زاده‌دیبازری ز, عابدسعیدی ژ, علوی‌مجد ح. بررسی کیفیت مراقبت های پس از زایمان در بیمارستان های تابعه دانشگاه علوم پزشکی و خدمات بهداشتی و درمانی شهید بهشتی در سال 1382. نشریه پژوهنده. 1384;10(1):9-15.
13. علیدوستی م, طهماسبی م, رئیسی م. بررسی رضایت مندی زنان پس از زایمان از خدمات و امکانات زایشگاه بیمارستان هاجر شهرکرد. مجله بالینی پرستاری و مامایی. 1392;2(1):1-8.
14. Brown SJ, Davey M-A, Bruinsma FJ. Women's views and experiences of postnatal hospital care in the Victorian Survey of Recent Mothers 2000. Midwifery. 2005;21(2):109-26.
15. Forster D, McLachlan H, Yelland J, Rayner J, Lumley JP, Pin C. A review of in-hospital postnatal care in Victoria. Final report Melbourne: La Trobe University. 2005.
16. Forster DA, McLachlan HL, Rayner J, Yelland J, Gold L, Rayner S. The early postnatal period: exploring women's views, expectations and experiences of care using focus groups in Victoria, Australia. BMC pregnancy and childbirth. 2008;8(1):1-11.
17. Arefadib N, Shafiei T, Cooklin A. Barriers and facilitators to supporting women with postnatal depression and anxiety: A qualitative study of maternal and child health nurses’ experiences. Journal of Clinical Nursing. 2023;32(3-4):397-408.
18. Sachdeva J, Yang SN, Gopalan P, Worley LL, Mittal L, Shirvani N, et al. Trauma Informed Care in the Obstetric Setting and Role of the Perinatal Psychiatrist: A Comprehensive Review of the Literature. Journal of the Academy of Consultation-Liaison Psychiatry. 2022;63(5):485-96.
19. Finlayson K, Crossland N, Bonet M, Downe S. What matters to women in the postnatal period: A meta-synthesis of qualitative studies. PloS one. 2020;15(4):e0231415.
20. Geuens S, Polona Mivšek A, Gianotten W. Midwifery and Sexuality: Springer Nature; 2023.
21. Pollock MA, Amankwaa LC, Amankwaa AA. First-time fathers and stressors in the postpartum period. The Journal of perinatal education. 2005;14(2):19-25.
22. Swanson V, Hannula L. Parenting stress in the early years–a survey of the impact of breastfeeding and social support for women in Finland and the UK. BMC Pregnancy and Childbirth. 2022;22(1):699.
23. Leahy‐Warren P, Philpott L, Elmir R, Schmied V. Fathers’ perceptions and experiences of support to be a parenting partner during the perinatal period: A scoping review. Journal of Clinical Nursing. 2022.
24. Lythgoe C, Lowe K, McCauley M, McCauley H. How women's experiences and perceptions of care influence uptake of postnatal care across sub-Saharan Africa: a qualitative systematic review. BMC Pregnancy and Childbirth. 2021;21(1):1-11.
25. Hui D, De La Cruz M, Mori M, Parsons HA, Kwon JH, Torres-Vigil I, et al. Concepts and definitions for “supportive care,”“best supportive care,”“palliative care,” and “hospice care” in the published literature, dictionaries, and textbooks. Supportive Care in Cancer. 2013;21:659-85.
26. Fisher C, Hauck Y, Fenwick J. How social context impacts on women's fears of childbirth: a Western Australian example. Social science & medicine. 2006;63(1):64-75.
27. Reibel T. Normal birth: a thing of the past or the new future for primary health care’. Primary Health Care Research & Development. 2004;5(4):329-37.
28. Reed R, Sharman R, Inglis C. Women’s descriptions of childbirth trauma relating to care provider actions and interactions. BMC pregnancy and childbirth. 2017;17:1-10.
29. Albanese AM, Geller PA, Sikes CA, Barkin JL. The importance of patient-centered research in the promotion of postpartum mental health. Frontiers in Psychiatry. 2021;12:720106.
30. van Teijlingen ER, Hundley V, Rennie AM, Graham W, Fitzmaurice A. Maternity satisfaction studies and their limitations:“What is, must still be best”. Birth. 2003;30(2):75-82.
31. Continuity of Care. Australia: Victorian Department of Human Services; 2004.
32. Perrenoud P, Chautems C, Kaech C. “Whatsapping” the continuity of postpartum care in Switzerland: A socio-anthropological study. Women and birth. 2022;35(3):e263-e74.
33. Van Teijlingen E. Maternity home care assistants in the Netherlands. Midwifery and the Medicalization of Childbirth: Comparative Perspectives; van Teijlingen, E, Lowis, G, McCaffery, P, Porter, M, Eds. 2004:163-72.
34. Delivery FC. Improving Maternity Services in England and Wales. Audit Commission. 1997.
35. Lefèvre M, Van den Heede K, Camberlin C, Bouckaert N, Beguin C, Devos C, et al. Impact of shortened length of stay for delivery on the required bed capacity in maternity services: results from forecast analysis on administrative data. BMC Health Services Research. 2019;19(1):1-9.
36. Derakhshanpour F, Kashani L, Taghavi S, Salimi Z, Shahini N. Effect of a Psycho education Interventions on Postpartum Health in North of IRAN. Journal of Clinical and Basic Research (JCBR). 2020;4(4).
37. Chen C-H, Tseng Y-F, Chou F-H, Wang S-Y. Effects of support group intervention in postnatally distressed women: A controlled study in Taiwan. Journal of psychosomatic research. 2000;49(6):395-9.
38. Glavin K, Smith L, Sørum R, Ellefsen B. Supportive counselling by public health nurses for women with postpartum depression. Journal of advanced nursing. 2010;66(6):1317-27.
39. Koçak V, Ege E, İyisoy MS. The development of the postpartum mobile support application and the effect of the application on mothers' anxiety and depression symptoms. Archives of psychiatric nursing. 2021;35(5):441-9.
40. Baghersad Z, Mokhtari F, Bahadoran P. Effect of home care on husband’s support during the postpartum period. Journal of Holistic Nursing And Midwifery. 2019;29(4):210-7.
41. Fenwick J, Butt J, Dhaliwal S, Hauck Y, Schmied V. Western Australian women's perceptions of the style and quality of midwifery postnatal care in hospital and at home. Women and birth. 2010;23(1):10-21.
42. Ghobadi M, Ziaee T, Mirhaghjo N, Pazandeh F. Evaluation of satisfaction with natural delivery experience and its related factors in Rasht women. Journal of Health and Care. 2018;20(3):215-24.
43. McLeish J, Harvey M, Redshaw M, Alderdice F. A qualitative study of first time mothers’ experiences of postnatal social support from health professionals in England. Women and Birth. 2021;34(5):e451-e60.
44. Adams YJ, Miller ML, Agbenyo JS, Ehla EE, Clinton GA. Postpartum care needs assessment: women’s understanding of postpartum care, practices, barriers, and educational needs. BMC Pregnancy and Childbirth. 2023;23(1):502.
45. Fathi Vajargah K. Educational need assessment: models and techniques. Tehran: Abeeze publication. 2005.
46. Berens P. Overview of the postpartum period: Normal physiology and routine maternal care. UptoDate. 2020;15:1-34.
47. Mengshoel AM. Mixed methods research–so far easier said than done? Manual Therapy. 2012;17(4):373-5.
48. حسنی م. در آمدی به روش شناسی تلفیقی در پژوهش های میان رشته ای علوم اجتماعی. فصلنامه مطالعات میان‌رشته‌ای در علوم انسانی. 1389;2(4):137-53.
49. Creswell JW, Creswell JD. Research design: Qualitative, quantitative, and mixed methods approaches: Sage publications; 2017.
50. Teddlie C, Tashakkori A. Foundations of mixed methods research: Integrating quantitative and qualitative approaches in the social and behavioral sciences: Sage; 2009.
51. Creswell JW, Clark VLP. Designing and conducting mixed methods research: Sage publications; 2017.
52. ضیایی م, زندی ا, عباس‌پور ن, عبدی م. توسعه‌ی پایدار گردشگری از دیدگاه دو مکتب ایده‌آلیسم و پراگماتیسم. برنامه ریزی و توسعه گردشگری. 1393;3(8):11-30.
53. Elo S, Kyngäs H. The qualitative content analysis process. Journal of advanced nursing. 2008;62(1):107-15.
54. Lauri S, Kyngas H. Developing nursing theories. Vantaa, Finland: Werner Söderström, Dark Oy. 2005.
55. Hsieh H-F, Shannon SE. Three approaches to qualitative content analysis. Qualitative health research. 2005;15(9):1277-88.
56. Graneheim UH, Lindgren B-M, Lundman B. Methodological challenges in qualitative content analysis: A discussion paper. Nurse education today. 2017;56:29-34.
57. Wildemuth BM. Applications of social research methods to questions in information and library science: Abc-Clio; 2016.
58. MacPhail A. Nominal group technique: a useful method for working with young people. British Educational Research Journal. 2001;27(2):161-70.
59. Manera K, Hanson CS, Gutman T, Tong A. Consensus methods: nominal group technique. 2019.
60. Speziale HS, Streubert HJ, Carpenter DR. Qualitative research in nursing: Advancing the humanistic imperative: Lippincott Williams & Wilkins; 2011.
61. Polit D, Beck C. Essentials of Nursing Research 6th edition. Philadephia MA. Lippincott; 2006.

Supervisors and Advisors

| **Name** | **Family** | **Name EN** | **Family EN** |  |  |  | **پست الکترونیک** |
| --- | --- | --- | --- | --- | --- | --- | --- |
| zahra | behboodi moghadam | zahra | behboodi moghadam | Second Advisor | School of Nursing and Midwifery | بهداشت باروری | behboodi@tums.ac.ir |
| Shirin | Shahbazi seyghaldeh | Shirin | Shahbazi seyghaldeh | First Advisor | School of Nursing and Midwifery | سلامت باروری | shahbazishirin@yahoo.com |
| Elham | Ebrahimi | Elham | Ebrahimi | First Supervisor | School of Nursing and Midwifery | بهداشت باروری | ebrahimi_308@yahoo.com |

Methods of study

**روش اجرا**

نوع مطالعه و روش اجرا
نوع مطالعه را مشخص فرمایید و در قسمت روش اجرا موارد مقابل بند مورد اشاره را توضیح دهید.

پژوهش حاضر یک مطالعه ترکیبی چند مرحله ای است، دلیل انتخاب رویکرد ترکیبی (جمع¬آوری هر دو نوع داده کیفی و کمی)، توسط محقق این بود که روشهای تحقیق ترکیبی بر مبنای رویکرد فلسفی عملگرا استواراست. بنیان فلسفه عملگرا تاکید بر پیامدهای تحقیق دارد و از نظر آن توجه به سوالی که در تحقیق پرسیده می شود مهمتر از توجه به روش تحقیق است. از این رو روش شناسی تحقیق ترکیبی، تبیین و تفیهم همزمان پیچیدگی¬ها، ابعاد، ساختار، اشکال پدیده¬ها و واقعیات اجتماعی است و استفاده از طرح¬ها، روش¬ها، نظریه¬ها و منابع داده¬های گوناگون و به عبارت دیگر استفاده از ترکیب روش¬های تحقیق کمی و کیفی، یک ضرورت هستی شناسی و معرفت شناسی است؛ زیرا هیچ روش تحقیقاتی به تنهایی کامل نیست و نمی¬تواند به همه ابعاد سوال تحقیق پاسخ دهد.
در سطح عملی، روش‌های ترکیبی رویکرد پیچیده‌ای را برای تحقیق فراهم می‌کند که برای کسانی که در خط مقدم رویه‌های تحقیقاتی جدید هستند جذابیت دارد. همچنین اگر محقق به داده¬های کمی و کیفی دسترسی داشته باشد، می¬تواند یک رویکرد ایده آل باشد. از آنجایی که روش تحقیق ترکیبی در علوم اجتماعی و انسانی به عنوان یک رویکرد تحقیق متمایز نسبتاً جدید است، ارایه یک تعریف و توصیف اساسی از رویکرد ترکیبی در روش انجام پژوهش مفید خواهد بود (49). پژوهش حاضر نیز نوعی مطالعه ترکیبی چند مرحله ای است که کرسول و کلارک (2017) آن را به عنوان یکی از پنج طرح اصلی مطالعات ترکیبی معرفی کرده اند (51). این پژوهش دارای سه بخش کیفی – طراحی مداخله و کمی خواهد بود.

مرحله اول بخش کیفی مطالعه
ابتدا یک مطالعه کیفی متوالی اکتشافی با رویکرد تحلیل محتوای قراردادی با هدف تبیین نیازهای مراقبت حمایتی زوجین در دوره پس از زایمان انجام خواهد شد
محیط پژوهش
محیط پژوهش تحقیق کیفی عرصه واقعی است. یعنی در محلی که افراد مورد نظر به سر می برند و تجربیات آنها آن روی می دهد و یا در محل هایی که توسط شرکت کنندگان انتخاب شود صورت میگیرد. محیط این پژوهش و محل بحث و مصاحبه با توجه به نظر مشارکت کنندگان و هماهنگی با سایر اعضای گروه عمدتا در بیمارستان ها و مراکز بهداشتی درمانی وابسته به دانشگاه علوم پزشکی شهر کرمانشاه خواهد بود.
جامعه پژوهش و مشارکت کنندگان :
از آنجا که تحقیق کیفی به دنبال بررسی معنای واقعیت است، مشارکت کنندگان افرادی هستند که منبع غنی از اطلاعات پدیده مورد بررسی هستند. جامعه مورد مطالعه در این پژوهش زنان دریافت کننده مراقبت های پس از زایمان و همسران آن ها در بیمارستان ها و مراکز بهداشتی درمانی وابسته به دانشگاه علوم پزشکی کرمانشاه که تجربه حداقل یک مورد زایمان (به شیوه طبیعی یا سزارین) و مراقبت پس از زایمان را داشته باشند و نیز ارایه دهندگان خدمات سلامت باروری شامل ماما های شاغل در بخش های بستری ، متخصصین زنان ،رزیدنت های زنان و پرستاران که حداقل دو سال سابقه ارایه خدمت به جمعیت مذکور را داشته باشند ونیز مدیران و سیاست گزاران حوزه سلامت باروری و حوزه سلامت زنان، روان شناسان و ماماهای شاغل در خارج از کشور خواهند بود و با نمونه گیری منطقی و مبتنی بر هدف با حداکثر تنوع انتخاب خواهند شد ونمونه گیری و مصاحبه ها تا اشباع اطلاعاتی داده ها ادامه خواهد یافت.
روش نمونه گیری:
افراد جهت انجام مصاحبه ها بصورت هدفمند با حداکثر تنوع انتخاب می شوند . نمونه گیری هدفمند به این معنی است که محقق در صدد انتخاب مشارکت کنندگانی است که دارای تجربیاتی در زمینه پدیده مورد پژوهش با مفاهیم کلیدی مورد اکتشاف هستند. برای رسیدن به حداکثر تنوع سعی می شود در مطالعه حاضر از مشارکت کنندگانی با سن و سطح تحصیلات گوناگون و وضعیت اجتماعی اقتصادی متفاوت و تنوع در رتبه بارداری و نوع زایمان ، جنسیت نوزاد، خواسته یا ناخواسته بودن بارداری، مدت زمان سپری شده از زایمان، وضعیت حمایتی فرد و... و در بخش ارایه دهندگان خدمات سلامت باروری از افرادی با سابقه اشتغال متفاوت استفاده می گردد
زنان زایمان کرده(به هردو شیوه زایمان طبیعی یا سزارین) در محل نمونه گیری (بیمارستان ها و مراکز بهداشتی وابسته به دانشگاه علوم پزشکی کرمانشاه) و همسران آنها که وضعیت جسمی و روانی و ارتباطی مناسب برای مصاحبه داشته، رضایت برای شرکت در مطالعه دارند و حداکثر هشت هفته از تولد فرزند آن ها گذشته باشد، وارد مطالعه خواهند شد. در بخش ارایه دهندگان خدمات سلامت باروری ، پرسنل بخش بهداشت و درمان شامل ماما های شاغل در بخش های مذکور ، متخصصین زنان ،رزیدنت های زنان و پرستاران که حداقل دو سال سابقه ارایه خدمت به جمعیت مذکور را داشته باشند،در صورت رضایت جهت شرکت در مطالعه، وارد مطالعه خواهند شد.گروه بعدی مورد مطالعه مدیران و سیاست گذاران در حوزه خدمات سلامت باروری و حوزه سلامت زنان، روان شناسان و ماماهای شاغل در خارج از کشور خواهد بود. پس از شرح اهداف مطالعه ضمن تاکید بر محرمانه ماندن اطلاعات، در صورت تمایل به شرکت در مطالعه هماهنگی از نظر زمان و مکان انجام مصاحبه صورت خواهد گرفت

معیارهای قابل قبول ورود به مطالعه
افرادی که در این پژوهش شرکت میکنند دارای ویژگی های زیر خواهند بود.
• دارا بودن حداقل سواد خواندن و نوشتن
• وضعیت جسمی، روانی وارتباطی مناسب جهت انجام مصاحبه
• قادر به برقراری ارتباط و مصاحبه باشند.
• ایرانی بوده و قادر به درک و صحبت زبان فارسی باشند.
• حداقل یک مورد سابقه زایمان و مراقبت پس از زایمان را داشته باشد.
• قرار داشتن در دوره پس از زایمان(حداکثر8 هفته پس از زایمان طبیعی یا سزارین)
• بستری بودن در بخش های پس از زایمان یکی از بیمارستان های تابعه دانشگاه علوم پزشکی کرمانشاه و یا مراجعه سرپایی به مراکز بهداشتی جهت دریافت مراقبت های پس از زایمان
• ارایه دهندگان خدمات سلامت باروری با حداقل دو سال سابقه کار
معیار های خروج از مطالعه
• ابتلا به بیماریهای روانی و اختلالات خلقی شناخته شده
• وقوع مرگ جنین یا نوزاد در پروسه زایمان اخیر
• ابتلای نوزاد به ناهنجاری شناخته شده
• رخداد حادثه ناگوار در 6 ماه گذشته در زندگی افراد

روش محاسبه حجم نمونه:
• در مطالعات کیفی محقق تا رسیدن به اشباع داده¬ها تا زمانی که هیچ داده جدیدی اضافه نشود به نمونه گیری ادامه می¬دهد. لذا در این مطالعه نیز نمونه گیری به صورت تدریجی تا اشباع داده¬ها ادامه می یابد.
روش جمع آوری داده ها:
روش گردآوری داده ها و ابزار گردآوری داده های کیفی
مصاحبه فردی
در مصاحبه های عمیق و نیمه ساختاریافته میران پاسخ دهی افراد بالا بوده و از صراحت بیان و آزادی بیشتر در ارایه نقطه نظرات خود برخوردار هستند. در این نوع مصاحبه، پژوهشگر یک راهنمای کلی یا فهرست بررسی تهیه می‌کند و براساس آن محور‌های کلی را از قبل مشخص می‌کند و هنگام مصاحبه برای هر محور سوال هایی در ذهن دارد. در ابتدا از تمام پاسخگو‌ها سوال‌های مشابهی پرسیده می‌شود، اما آنها آزادند که پاسخ خود را به هر طریقی که مایلند ارائه دهند، یعنی در حالی که ساختار کلی مصاحبه برای همه شرکت کنندگان یکسان است، اما مصاحبه کننده می‌تواند در مواقع لزوم سوال‌های دیگری بپرسد.، در این مورد مسئولیت رمز گردانی پاسخ‌ها و طبقه بندی آنها بر عهده پژوهشگر است. در این نوع مصاحبه پژوهشگر به دنبال اطلاعات خاصی است تا بتواند آنها را با اطلاعاتی که از دیگر مصاحبه‌ها به دست می‌آید مقایسه و مقابله کند.
در مطالعه حاضر برای تبیین نیازهای مراقبت حمایتی زوجین در دوره پس از زایمان نیز از این رویکرد استفاده خواهد شد. مشارکت کنندگان مبتنی بر هدف انتخاب خواهند شد. سعی بر آن خواهد شد که با رعایت تناوب(تنوع) حداکثری با زنان دارای یک یا چند سابقه زایمان و همسران آن ها و نیز ارایه دهندگان خدمات سلامت باروری در حیطه ها و رده های مختلف شغلی (ماما، پرستار، پزشک، مسئولین و مدیران) با سوابق متعدد مصاحبه به عمل آید. مصاحبه¬های فردی با مطلعین کلیدی (مدیران و سیاست گذاران حوزه سلامت باروری، سیاست گذاران حوزه سلامت زنان، روان شناسان، ماماهای شاغل در خارج از کشور) نیز انجام خواهد شد. بعد از تایید نهایی پروپوزال تحقیقاتی توسط شورای تحصیلات تکمیلی دانشگاه تخصصی مربوطه وکسب تاییدیه علمی اخلاقی از دانشگاه علوم پزشکی تهران برای شروع اجرای تحقیق و گردآوری داده ها از معاونت آموزشی و نیز معاونت به درمان موافقت کتبی اخذ خواهد شد. نمونه گیری با مراجعه پژوهشگر به مراکز بهداشتی درمانی و بیمارستانهای وابسته به دانشگاه علوم پزشکی کرمانشاه آغاز خواهد شد. پس از تماس با مشارکت کنندگان پژوهشگر خود را معرفی کرده و با توضیح اهداف از انجام مطالعه، زمان و مکان مصاحبه با آنها هماهنگ خواهد شد. جهت مصاحبه با مشارکت کننده ، رابطه دوستانه برقرار خواهد شد و به آنها در مورد محرمانه ماندن اطلاعات اطمینان داده خواهد شد و پس از تفهیم هدف انجام مطالعه از شرکت کنندگان در تحقیق رضایت نامه کسب خواهد گردید. مصاحبه ها با چند سوال باز آغاز خواهد شد. در ادامه بر اساس پاسخ¬های اولیه و بر اساس راهنمای مصاحبه نیازهای مراقبت حمایتی در دوره بعد از زایمان و همچنین ارائه پیشنهاداتی در رفع این نیازها مورد پرسش قرار خواهد گرفت. همچنین بر حسب نیاز در مصاحبه¬ها از سوالات کاوشی نظیر «منظور شما چیست؟» و یا «اگر می¬توانید لطفاً بیشتر توضیح دهید» استفاده خواهد شد. با توجه به راهنمای مصاحبه در ابتدا سوالات ساده و کلی¬تر مطرح می¬شود و با توجه به نحوه پاسخگویی و تجربیات مشارکت کنندگان مصاحبه به سمت سوالات جزئی¬تر ادامه می یابد. سوالات مصاحبه قابلیت انعطاف داشته و در صورت نیاز سوالات جدید به راهنمای مصاحبه اضافه خواهد شد در پژوهش حاضر حین مصاحبه ها، مشاهده و یادداشت برداری در عرصه جهت بررسی نحوه ارایه مراقبت ها و تعاملات ارایه دهندگان خدمت با زنان زایمان کرده و همسران آن ها نیز صورت خواهد گرفت. به کلیه حرکات و واکنش های احساسی و رفتاری مصاحبه شونده و محیط توجه خواهد شد. پس از پایان، مصاحبه های ضبط شده و یادداشت برداری های انجام شده، پیاده و تحلیل می شوند.
سوالات مربوط به زوجین در دوره پس از زایمان
1- تجربه و احساس خود در مورد مراقبت هایی که بعد از زایمان به شما ارایه شد را توضیح دهید؟
3- فکر می کنید باید به چه مواردی در این مراقبت ها توجه می شد؟
4- چه انتظاراتی از پزشک، پرستار و ماماها داشتید؟
5-چه نیازهایی در این مدت داشته اید و فکر می کنید خدمت خاصی برای آن ها وجود نداشته است و یا لازم است در این موارد هم خدماتی به زوجین و خانواده ها ارائه شود؟
6-در مورد خودتان و همسرتان و نوزاد با چه مشکلاتی در دوره بعد از زایمان رو به رو شدید؟
7-چگونه این مشکلات را برطرف کردید.
8-برای برطر ف کردن مشکلات و نیازهای خود به ارائه کنندگان خدمات بهداشتی و پزشکی مراجعه کردید؟ اگر بله لطفا نظر و تجربه خودتان را از دریافت این خدمات مطرح کنید.
9-نیازهای اطلاعاتی شما در این مدت چه بودند و از چه منابعی نیازهای خود را برطرف کردید؟ آیا این منابع با کیفیت بودند و نیاز شما را به خوبی و کامل برطرف کردند؟ آیا نقصی داشتند؟ لطفا توضیح دهید.
10-نیازهای حمایتی و عاطفی شما در این مدت چه بودند و از چه منابعی نیازهای خود را برطرف کردید؟ آیا این منابع با کیفیت بودند و نیاز شما را به خوبی و کامل برطرف کردند؟ آیا نقصی داشتند؟ لطفا توضیح دهید.
11-نیازهای جسمی و مراقبت های جسمی شما در این مدت چه بودند و از چه منابعی نیازهای خود را برطرف کردید؟ آیا این منابع با کیفیت بودند و نیاز شما را به خوبی و کامل برطرف کردند؟ آیا نقصی داشتند؟ لطفا توضیح دهید.
12-توانایی های خودتان را چطور دیدید؟ چه مشکلاتی در خودتان احساس کردید؟
13-در حال حاضر چه نگرانی هایی دارید؟
و........
سوالات مربوط به ارایه دهندگان خدمات سلامت باروری:
1-شما چه خدماتی به خانم ها در دوره پس از زایمان ارایه می دهید؟ یک روز کاری خودتان را درخصوص ارائه مراقبت های پس از زایمان شرح دهید.
2-حمایت ها و مراقبت های جسمانی، اطلاعاتی، عاطفی در دوره بعد از زایمان شامل چه مواردی است و آیا از نظر شما این حمایت و مراقبت ها کامل است و به خوبی ارائه می شود یا نقص و کمبود دارد. لطفا شرح دهید.
3- به نظر شما مسایل و دغدغه های مربوط به سلامت زوجین در دوره بعداز زایمان و دلایل آن چیست؟ توضیح دهید.
4-چه نیازهایی از سلامت زوجین در ابعاد جسمی و روانی در دوره بعد از زایمان در این مرکز برآورده می شود؟
5-به نظر شما زوجین در دوره پس از زایمان چه نیازهایی دارند که در مراکز بیمارستانی برآورده نمی شود؟
6-در مورد دسترسی زوجین به خدمات و مراقبت حمایتی در دوره پس از زایمان توضیح دهید؟
7-تمایل زوجین به برآورده شدن کدام نیازهای مراقبت حمایتی بیشتر است؟
8-شما به عنوان ارایه دهنده خدمات سلامت باروری چه موانعی را بر سر راه ارایه خدمت خود در راستای نیازهای مراقبت حمایتی به این زنان می بینید؟
9-شما به عنوان کسی که با مشکلات زوجین در دوره پس از زایمان آشنا هستید چه نیازهایی در حوزه حمایت پس از زایمان می شناسید که پاسخ داده نشده است؟
سوالات مربوط به روان شناسان:
1-تجربه شما از اختلالات روانی که خانم ها و همسران آن ها در دوره بعد از زایمان به شما مراجعه می کنند چیست؟
2-زوجین در دوره بعد از زایمان معمولا در چه مواقع و شرایطی به شما مراجعه می کنند؟ مشکلات آن ها در این دوره در چه حیطه هایی است؟
3-در صورت ابتلا زوجین به اختلالات روان شناختی در دوره پس از زایمان، فکر می کنید علت ابتلا آن ها به این مسایل چیست؟
4-مراقبت های داخل بیمارستان و مراکز بهداشتی و مراقبت های ارایه شده توسط همسر و سایر اعضای سیستم حمایتی چقدر می تواند در کاهش و پیشگیری ازین مشکلات مفید باشد؟
سوالات مربوط به سیاست گذاران:
1-اطلاع دارید چه خدماتی برای دوره بعد از زایمان در ایران ارائه می شود؟ لطفا توضیح دهید.
2-آیا می دانید تفاوت این خدمات نسبت به دیگر کشورهای پیشرفته چیست و چه کمبودها و نقص هایی وجود دارد؟
3-به نظر شما امکان برطرف کردن این نقص ها و کمبودها وجود دارد؟ و چگونه؟
4-لطفا در مورد این که بعضی از خدمات و مراقبت ها وجود ندارد یا نقص ندارد و اگر وجودمی داشت، چه تفاوتی به لحاظ اقتصادی داشت توضیح دهید. آیا کشورهای پیشرفته که خدمات بهتری دارند، به صرفه اقتصادی هم توجه دارند؟ چگونه؟
5- چه دستورالعمل هایی برای مراقبت های پس از زایمان ارائه شده و چه مواردی در حال تدوین است؟ نحوه نظارت بر این دستورالعمل ها ، ارزشیابی و بروز رسانی را توضیح دهید؟
ادامه سوالات مربوط به بخش سیاست گذاران، پس از مصاحبه با زوجین و بر اساس نیاز های تبیین شده از سوی آن ها، طراحی خواهد شد.
پس از پایان سوالات مصاحبه، از مشارکت‌کننده خواسته خواهد شد که اگر نکته و صحبتی باقی مانده، بیان کند. در پایان با او در مورد احتمال مصاحبه‌های بعدی صحبت خواهد شد.
مصاحبه‌ها با کسب اجازه از مشارکت‌کننده و اطمینان دادن مبنی بر این که کلیه مصاحبه ها محرمانه خواهند بود، ضبط و سپس کلمه به کلمه پیاده خواهند شد و برای کنترل صحّت آنها، متن مصاحبه به مشارکت کننده داده خواهد شد تا آن را خوانده و تایید نماید.
روش تحلیل داده ها
در تعاریف اولیه تحلیل محتوی به عنوان روشی برای تحلیل نوشته ها، متون ، ارتباطات کلامی و غیر کلامی توصیف شده است (53). این تحلیل هم در مورد داده های کمی و هم به داده های کیفی قابل به کار گیری است. به علاوه این تحلیل به صورت قیاسی و یا استقرایی نیز قابل انجام است. انتخاب هر یک بستگی به هدف مطالعه دارد . اگر دانش موجود در مورد موضوع مورد مطالعه کم با پراکنده باشد ، توصیه می شود که تحلیل محتوی به روش استقرایی انجام شود (54).
این روش به سه دسته، شامل قراردادی ، جهت دار و متراکم طبقه بندی می شود که در مطالعه حاضر، ما از روش تحلیل محتوای قراردادی ( مرسوم) استفاده خواهیم کرد. در این روش، داده ها به صورت مستقیم از شرکت کنندگان در مطالعه کسب می شوند و طبقه ها از قبل تعیین نمی گردند، بلکه برگرفته از متن داده ها هستند. بنابراین تحلیل داده -ها با خواندن مکرر تمام داده ها شروع می شود تا محقق در داده ها غرق شود و یک دید کلی بدست آورد. سپس داده -ها کلمه به کلمه خوانده می شوند تا کلماتی از متن که در بر گیرنده مفاهیم کلیدی است برجسته گشته و بدین ترتیب کدها استخراج گردند. سپس محقق اولین تفاسیر خود را بر روی متن می گذارد و تحلیل اولیه را انجام می دهد. همچنانکه این فرایند ادامه می یابد، برچسبهایی برای کدها پدیدار می گردد که منعکس کننده بیش از یک مفهوم کلیدی هستند و معمولا مستقیما از متن برگرفته می شوند و بعدا به نقشه کدگذاری اولیه تبدیل می شوند. سپس کدها بر اساس آنکه کدهای مختلف چگونه با هم مرتبط هستند در طبقه جای داده می شوند. این طبقات پدید آمده برای سازمان دهی و گروه بندی کدها به صورت خوشه هایی با معنی استفاده می شوند. به طور ایده آل تعداد طبقات بین 10-15 است، تا وسعت کافی برای در بر گرفتن تعداد زیادی از کدها را داشته باشد. می توان تعداد زیادتری از طبقات را بر اساس ارتباط بین آنها به تعداد کمتری از زیر طبقات ترکیب کرد. یک دیاگرام درختی می تواند در سازمان دهی این طبقات به یک ساختار سلسله مراتبی کمک کند . سپس برای هر طبقه، زیر طبقه و کد ، تعاریفی خلق می شود. به هنگام گزارش یافته ها مثال هایی از هر کد و طبقه از داده ها آورده می شود (55). در این مطالعه پس از انجام هر مصاحبه متن مصاحبه در اولین فرصت و ترجیحاً در روز مصاحبه پیاده سازی خواهد شد. پس از پیاده کردن هر مصاحبه اساتید محترم راهنما و مشاور متن مصاحبه را مجدداً مورد مطالعه قرار می دهند. کدهای حاصل با استفاده از نرم افزار MAXQDA 12 مدیریت می-شود.
تحلیل محتوای قراردادی در این مطالعه بر اساس روش پیشنهادی ژانک و ویلموس(2016) در هشت مرحله بدین صورت آنالیز خواهد شد:
1-آماده کردن داده ها برای تحلیل محتوای کیفی
2-تصمیم گیری در خصوص واحد آنالیز
3-طبقه بندی
4- آزمودن کدگذاری در نمونه ای از متن
5-گسترش فرآیند آزمودن کدگذاری به کل متن
6-دسترسی به ثبات کدگذاری
7- نتیجه گیری از داده های طبقه بندی شده یا کد شده
8- مرحله گزارش دهی
در مرحله ی بعدی، آزمون کدها و ثبات آن در متن است که با بررسی کدها و توافق اکثریت اعضای تیم تحقیق بر این کدها، مورد تایید قرار خواهد گرفت. جهت بررسی ثبات کد گذاری ، دو نفر متخصص مجرب خارج از تیم تحقیق کدهای داده شده به طبقات و زیر طبقات را کنترل خواهند کرد. مرحله ی بعدی شامل نتیجه گیری در مورد انجام صحیح طبقه بندی داده ها و کدهای داده شده خواهد بود که ویژگی ها و روابط در هر طبقه و بین طبقات مورد بررسی و تحلیل قرار خواهد گرفت. هر طبقه و زیر طبقه با متن مصاحبه نیز مورد تحلیل قرار خواهد گرفت و در نهایت طبقات شکل گرفته تفسیر و گزارش داده خواهد شد (56, 57).
مرحله دوم: بخش طراحی مداخله
دراین مرحله از پژوهش، طراحی مداخله جهت تبیین نیازهای مراقبت حمایتی زوجین ایرانی در دوره پس از زایمان ، در دو بخش انجام خواهد شد:
بخش اول : پنل صاحب نظران(تشکیل گروه اسمی) در راستای اولویت بندی نیاز های تبیین شده در بخش کیفی مطالعه
در راستای طراحی مداخله و برقراری ارتباط بین بخش کیفی و کمی مطالعه ، مسایل و نیازهای مراقبت حمایتی زوجین در جلسه ای با حضور متخصصان سلامت باروری، سیاست گذاران سلامت باروری، اساتید راهنما و دارورها به شیوه گروه اسمی مورد اولویت بندی قرار خواهند گرفت

با توجه به نیازهای مراقبت حمایتی تبیین شده در مرحله اول، مهم¬ترین نیازهای بدست آمده در این زمینه در جلسه¬ای متشکل از تیم تحقیق و گروهی از متخصصین و برنامه¬ریزان سلامت (شامل سیاستگذاران ، ارایه دهندگان خدمات سلامت باروری، اساتید راهنما و مشاور و ...) مورد اولویت¬بندی قرار می¬گیرد. تکنیک گروه اسمی (NGT) روشی است که برای تولید ایده و رسیدن به اجماع استفاده می شود. این تکنیک در اواخر دهه 1960 توسط آندره دلبک و اندرو ون دی ون توسعه یافت و از آن زمان در محیط های مختلف، از تجارت گرفته تا آموزش و دولت مورد استفاده قرار گرفته است. در واقع تکنیک گروه اسمی (NGT) یک فرآیند طوفان فکری ساختاریافته است که همه اعضای گروه را تشویق می‌کند تا ایده‌های خود را به طور یکسان به اشتراک بگذارند. این تکنیک تصمیم گیری می تواند ایده ایجاد کند، مشکلات را حل کند یا تصمیم بگیرد
ساختار اصلی NGT شامل چهار مرحله است: طوفان فکری، رای دادن و کاهش ایده، بحث و اولویت بندی ایده و ایجاد اجماع نظر . شرکت کنندگان تشویق می شوند تا در مرحله اول تا حد امکان ایده های خود را بدون قضاوت یا بحث ایجاد کنند. در مرحله دوم، شرکت کنندگان با یکدیگر همکاری می کنند تا فهرست ایده ها را به تعداد قابل مدیریت کاهش دهند. در مرحله سوم، هر شرکت کننده ایده های باقی مانده را به ترتیب اهمیت رتبه بندی می کند. در نهایت، در گام چهارم، گروه برای رسیدن به اجماع بر سر مهم ترین ایده ها با یکدیگر همکاری می کنند. NGT ابزاری موثر برای تولید ایده های جدید و رسیدن به توافق در یک گروه است (58). یک تسهیل‌کننده از شرکت‌کنندگان می‌خواهد که به‌صورت جداگانه ایده‌ها را شناسایی کرده و برای ایجاد فهرست در پاسخ به سوالی خاص مشارکت کنند، بنابراین از تسلط شرکت¬کنندگان بر بحث جلوگیری می¬کند و امکان ابراز عقیده برای همه افراد گروه به طور یکسان وجود دارد (59).
در این مطالعه در شروع جلسه پس از بیان مقدمه¬ای مبنی بر هدف از تشکیل جلسه، نتایج استخراج شده از بخش کیفی مطالعه ارایه می¬گردد، سپس از مدعوین درخواست می¬شود تا نظرات خود را نسبت به نیازهای مراقبت حمایتی زوجین در دوره پس از زایمان به طور خصوصی یادداشت کنند. سپس لیستی از ایده¬های هر یک از اعضای گروه در یک جدول نوشته و به اشتراک گذاشته خواهد شد. در نهایت در مورد ایده¬ها رای¬گیری انجام و شایع ترین نیازها شناسایی می¬شود.

دومین بخش :بررسی متون و تدوین مداخله مناسب
در این مرحله جهت طراحی مداخله ابتدا با در نظر داشتن اولویت اصلی تعیین شده در مرحله قبل، به بررسی و مرور مداخلات و برنامه‌های مربوط به رفع نیازهای مراقبت حمایتی زوجین در دوره پس از زایمان در ایران و سایر کشورها، پرداخته خواهد شد در واقع یک مرور متون در زمینه اولویت دارترین نیاز های زوجین در دوره پس از زایمان انجام خواهد شد. بر این‌ اساس به جستجو در بانک‌های اطلاعاتی SID؛ Magiran؛ Iran Medex؛ ProQuest؛ Pubmed؛ Google Scholar Embase; وweb of science و سایت‌های اختصاصی WHO ; UNAIDS; IPPF; ;UNFPAl بین سال‌های 2000 تا 2023 پرداخته خواهد شد. سپس مداخلات و برنامه‌های مرتبط در جلسه ای به شیوه گروه اسمی (NGT) ، متشکل از تیم تحقیق و گروهی از متخصصین علاقه مند مورد بررسی قرار خواهد گرفت و سپس مناسب ترین مداخله انتخاب خواهد شد.
نوع مداخله:
مداخله پیشنهادی در راستای مهم ترین عوامل ریشه ای هستند که با نارضایتی زنان از مراقبت های پس از زایمان در ارتباطند و به عنوان مهم ترین موانع دستیابی به سلامت مطلوب در حوزه خدمات حول و حوش تولد و پس از زایمان تلقی می شوند از قبیل الگوی تکنوکراتیک( زیست پزشکی) حاکم بر سیستم بهداشت و درمان کشور و پزشکی سازی پروسه تولد، محدودیت تعداد پرسنل،اطلاعات ناکافی پرسنل، عدم اجازه به مادر جهت در آغوش کشیدن و تماس با نوزاد بلافاصله پس از زایمان، طول اقامت غیر قابل انعطاف، در دسترس بودن پشتیبانی حرفه ای و نیز چالش های حمایتی از قبیل عدم وجود امکانات مشاوره در راستای موضوعاتی مانند سازگاری با تغییرات در روابط صمیمی و خانوادگی خود، از سرگیری روابط جنسی، مهارت تطابق با فرزندپروری، و مقابله با ترومای زایمانی تجربه شده، و رفع نگرانی هایی مانند آسیب به دستگاه تناسلی، تصویر ذهنی از بدن و دیگر موانع ارایه مراقبت های زن محور خواهد بود.
همانطور که ذکر شد در راستای طراحی و اعتباریابی مداخله، مسایل و نیازهای مراقبت حمایتی تبیین شده زوجین در بخش کیفی مطالعه، در جلسه ای با حضور متخصصان سلامت باروری، سیاست گذاران سلامت باروری، اساتید راهنما و دارورها به شیوه گروه اسمی مورد اولویت بندی قرار خواهند گرفت. سپس با در نظر داشتن اولویت اصلی تعیین شده در مرحله قبل، به بررسی و مرور مداخلات و برنامه‌های مربوط به رفع نیازهای مراقبت حمایتی زوجین در دوره پس از زایمان در ایران و سایر کشورها، پرداخته خواهد شد. سپس مناسب ترین مداخله (که به صورت کارآزمایی بالینی خواهد بود) در جلسه گروه اسمی انتخاب خواهد شد. اعتباریابی و ارزیابی مداخله با استفاده از ابزار تایدر صورت خواهد پذیرفت. پس از انتخاب مداخله و اجرا، جهت ارزشیابی از پرسشنامه های استاندارد موجود که در حوزه رضایت سنجی مراقبت های حمایتی هستند با توجه به متغیر های انتخابی در بخش کمی مطالعه استفاده خواهد شد .
مرحله سوم: بخش کمّی مطالعه (آزمون مداخله)
مرحله سوم این پژوهش یک مطالعه کمّی خواهد بود. این مرحله با هدف تعیین تاثیر مداخله‌ی طراحی شده بر نیاز های مراقبت حمایتی زوجین ایرانی در دوره پس از زایمان انجام خواهد شد. شایان ذکر است که نوع مطالعه کمی و سایر جزییات مرتبط با آن در این مرحله بر اساس نوع مداخله انتخاب شده پس از اولویت بندی نیازها و مرور متون تعیین خواهد شد. پس از انجام مطالعه مداخله ای، داده های کمی با استفاده از نرم افزار SPSS مورد تجزیه و تحلیل قرار خواهند گرفت.

**محل انجام مطالعه**

Outside the university

**مشخصات ابزار جمع آوری اطلاعات و نحوه جمع آوری**

روش جمع آوری داده ها:
روش گردآوری داده ها و ابزار گردآوری داده های کیفی
مصاحبه فردی
در مصاحبه های عمیق و نیمه ساختاریافته میران پاسخ دهی افراد بالا بوده و از صراحت بیان و آزادی بیشتر در ارایه نقطه نظرات خود برخوردار هستند. در این نوع مصاحبه، پژوهشگر یک راهنمای کلی یا فهرست بررسی تهیه می‌کند و براساس آن محور‌های کلی را از قبل مشخص می‌کند و هنگام مصاحبه برای هر محور سوال هایی در ذهن دارد. در ابتدا از تمام پاسخگو‌ها سوال‌های مشابهی پرسیده می‌شود، اما آنها آزادند که پاسخ خود را به هر طریقی که مایلند ارائه دهند، یعنی در حالی که ساختار کلی مصاحبه برای همه شرکت کنندگان یکسان است، اما مصاحبه کننده می‌تواند در مواقع لزوم سوال‌های دیگری بپرسد.، در این مورد مسئولیت رمز گردانی پاسخ‌ها و طبقه بندی آنها بر عهده پژوهشگر است. در این نوع مصاحبه پژوهشگر به دنبال اطلاعات خاصی است تا بتواند آنها را با اطلاعاتی که از دیگر مصاحبه‌ها به دست می‌آید مقایسه و مقابله کند.
در مطالعه حاضر برای تبیین نیازهای مراقبت حمایتی زوجین در دوره پس از زایمان نیز از این رویکرد استفاده خواهد شد. مشارکت کنندگان مبتنی بر هدف انتخاب خواهند شد. سعی بر آن خواهد شد که با رعایت تناوب(تنوع) حداکثری با زنان دارای یک یا چند سابقه زایمان و همسران آن ها و نیز ارایه دهندگان خدمات سلامت باروری در حیطه ها و رده های مختلف شغلی (ماما، پرستار، پزشک، مسئولین و مدیران) با سوابق متعدد مصاحبه به عمل آید. مصاحبه¬های فردی با مطلعین کلیدی (مدیران و سیاست گذاران حوزه سلامت باروری، سیاست گذاران حوزه سلامت زنان، روان شناسان، ماماهای شاغل در خارج از کشور) نیز انجام خواهد شد. بعد از تایید نهایی پروپوزال تحقیقاتی توسط شورای تحصیلات تکمیلی دانشگاه تخصصی مربوطه وکسب تاییدیه علمی اخلاقی از دانشگاه علوم پزشکی تهران برای شروع اجرای تحقیق و گردآوری داده ها از معاونت آموزشی و نیز معاونت به درمان موافقت کتبی اخذ خواهد شد. نمونه گیری با مراجعه پژوهشگر به مراکز بهداشتی درمانی و بیمارستانهای وابسته به دانشگاه علوم پزشکی کرمانشاه آغاز خواهد شد. پس از تماس با مشارکت کنندگان پژوهشگر خود را معرفی کرده و با توضیح اهداف از انجام مطالعه، زمان و مکان مصاحبه با آنها هماهنگ خواهد شد. جهت مصاحبه با مشارکت کننده ، رابطه دوستانه برقرار خواهد شد و به آنها در مورد محرمانه ماندن اطلاعات اطمینان داده خواهد شد و پس از تفهیم هدف انجام مطالعه از شرکت کنندگان در تحقیق رضایت نامه کسب خواهد گردید. مصاحبه ها با چند سوال باز آغاز خواهد شد. در ادامه بر اساس پاسخ¬های اولیه و بر اساس راهنمای مصاحبه نیازهای مراقبت حمایتی در دوره بعد از زایمان و همچنین ارائه پیشنهاداتی در رفع این نیازها مورد پرسش قرار خواهد گرفت. همچنین بر حسب نیاز در مصاحبه¬ها از سوالات کاوشی نظیر «منظور شما چیست؟» و یا «اگر می¬توانید لطفاً بیشتر توضیح دهید» استفاده خواهد شد. با توجه به راهنمای مصاحبه در ابتدا سوالات ساده و کلی¬تر مطرح می¬شود و با توجه به نحوه پاسخگویی و تجربیات مشارکت کنندگان مصاحبه به سمت سوالات جزئی¬تر ادامه می یابد. سوالات مصاحبه قابلیت انعطاف داشته و در صورت نیاز سوالات جدید به راهنمای مصاحبه اضافه خواهد شد در پژوهش حاضر حین مصاحبه ها، مشاهده و یادداشت برداری در عرصه جهت بررسی نحوه ارایه مراقبت ها و تعاملات ارایه دهندگان خدمت با زنان زایمان کرده و همسران آن ها نیز صورت خواهد گرفت. به کلیه حرکات و واکنش های احساسی و رفتاری مصاحبه شونده و محیط توجه خواهد شد. پس از پایان، مصاحبه های ضبط شده و یادداشت برداری های انجام شده، پیاده و تحلیل می شوند.
سوالات مربوط به زوجین در دوره پس از زایمان
1- تجربه و احساس خود در مورد مراقبت هایی که بعد از زایمان به شما ارایه شد را توضیح دهید؟
3- فکر می کنید باید به چه مواردی در این مراقبت ها توجه می شد؟
4- چه انتظاراتی از پزشک، پرستار و ماماها داشتید؟
5-چه نیازهایی در این مدت داشته اید و فکر می کنید خدمت خاصی برای آن ها وجود نداشته است و یا لازم است در این موارد هم خدماتی به زوجین و خانواده ها ارائه شود؟
6-در مورد خودتان و همسرتان و نوزاد با چه مشکلاتی در دوره بعد از زایمان رو به رو شدید؟
7-چگونه این مشکلات را برطرف کردید.
8-برای برطر ف کردن مشکلات و نیازهای خود به ارائه کنندگان خدمات بهداشتی و پزشکی مراجعه کردید؟ اگر بله لطفا نظر و تجربه خودتان را از دریافت این خدمات مطرح کنید.
9-نیازهای اطلاعاتی شما در این مدت چه بودند و از چه منابعی نیازهای خود را برطرف کردید؟ آیا این منابع با کیفیت بودند و نیاز شما را به خوبی و کامل برطرف کردند؟ آیا نقصی داشتند؟ لطفا توضیح دهید.
10-نیازهای حمایتی و عاطفی شما در این مدت چه بودند و از چه منابعی نیازهای خود را برطرف کردید؟ آیا این منابع با کیفیت بودند و نیاز شما را به خوبی و کامل برطرف کردند؟ آیا نقصی داشتند؟ لطفا توضیح دهید.
11-نیازهای جسمی و مراقبت های جسمی شما در این مدت چه بودند و از چه منابعی نیازهای خود را برطرف کردید؟ آیا این منابع با کیفیت بودند و نیاز شما را به خوبی و کامل برطرف کردند؟ آیا نقصی داشتند؟ لطفا توضیح دهید.
12-توانایی های خودتان را چطور دیدید؟ چه مشکلاتی در خودتان احساس کردید؟
13-در حال حاضر چه نگرانی هایی دارید؟
و........
سوالات مربوط به ارایه دهندگان خدمات سلامت باروری:
1-شما چه خدماتی به خانم ها در دوره پس از زایمان ارایه می دهید؟ یک روز کاری خودتان را درخصوص ارائه مراقبت های پس از زایمان شرح دهید.
2-حمایت ها و مراقبت های جسمانی، اطلاعاتی، عاطفی در دوره بعد از زایمان شامل چه مواردی است و آیا از نظر شما این حمایت و مراقبت ها کامل است و به خوبی ارائه می شود یا نقص و کمبود دارد. لطفا شرح دهید.
3- به نظر شما مسایل و دغدغه های مربوط به سلامت زوجین در دوره بعداز زایمان و دلایل آن چیست؟ توضیح دهید.
4-چه نیازهایی از سلامت زوجین در ابعاد جسمی و روانی در دوره بعد از زایمان در این مرکز برآورده می شود؟
5-به نظر شما زوجین در دوره پس از زایمان چه نیازهایی دارند که در مراکز بیمارستانی برآورده نمی شود؟
6-در مورد دسترسی زوجین به خدمات و مراقبت حمایتی در دوره پس از زایمان توضیح دهید؟
7-تمایل زوجین به برآورده شدن کدام نیازهای مراقبت حمایتی بیشتر است؟
8-شما به عنوان ارایه دهنده خدمات سلامت باروری چه موانعی را بر سر راه ارایه خدمت خود در راستای نیازهای مراقبت حمایتی به این زنان می بینید؟
9-شما به عنوان کسی که با مشکلات زوجین در دوره پس از زایمان آشنا هستید چه نیازهایی در حوزه حمایت پس از زایمان می شناسید که پاسخ داده نشده است؟
سوالات مربوط به روان شناسان:
1-تجربه شما از اختلالات روانی که خانم ها و همسران آن ها در دوره بعد از زایمان به شما مراجعه می کنند چیست؟
2-زوجین در دوره بعد از زایمان معمولا در چه مواقع و شرایطی به شما مراجعه می کنند؟ مشکلات آن ها در این دوره در چه حیطه هایی است؟
3-در صورت ابتلا زوجین به اختلالات روان شناختی در دوره پس از زایمان، فکر می کنید علت ابتلا آن ها به این مسایل چیست؟
4-مراقبت های داخل بیمارستان و مراکز بهداشتی و مراقبت های ارایه شده توسط همسر و سایر اعضای سیستم حمایتی چقدر می تواند در کاهش و پیشگیری ازین مشکلات مفید باشد؟
سوالات مربوط به سیاست گذاران:
1-اطلاع دارید چه خدماتی برای دوره بعد از زایمان در ایران ارائه می شود؟ لطفا توضیح دهید.
2-آیا می دانید تفاوت این خدمات نسبت به دیگر کشورهای پیشرفته چیست و چه کمبودها و نقص هایی وجود دارد؟
3-به نظر شما امکان برطرف کردن این نقص ها و کمبودها وجود دارد؟ و چگونه؟
4-لطفا در مورد این که بعضی از خدمات و مراقبت ها وجود ندارد یا نقص ندارد و اگر وجودمی داشت، چه تفاوتی به لحاظ اقتصادی داشت توضیح دهید. آیا کشورهای پیشرفته که خدمات بهتری دارند، به صرفه اقتصادی هم توجه دارند؟ چگونه؟
5- چه دستورالعمل هایی برای مراقبت های پس از زایمان ارائه شده و چه مواردی در حال تدوین است؟ نحوه نظارت بر این دستورالعمل ها ، ارزشیابی و بروز رسانی را توضیح دهید؟
ادامه سوالات مربوط به بخش سیاست گذاران، پس از مصاحبه با زوجین و بر اساس نیاز های تبیین شده از سوی آن ها، طراحی خواهد شد.
پس از پایان سوالات مصاحبه، از مشارکت‌کننده خواسته خواهد شد که اگر نکته و صحبتی باقی مانده، بیان کند. در پایان با او در مورد احتمال مصاحبه‌های بعدی صحبت خواهد شد.
مصاحبه‌ها با کسب اجازه از مشارکت‌کننده و اطمینان دادن مبنی بر این که کلیه مصاحبه ها محرمانه خواهند بود، ضبط و سپس کلمه به کلمه پیاده خواهند شد و برای کنترل صحّت آنها، متن مصاحبه به مشارکت کننده داده خواهد شد تا آن را خوانده و تایید نماید.

**روش محاسبه حجم نمونه و تعداد آن**

روش محاسبه حجم نمونه:
• در مطالعات کیفی محقق تا رسیدن به اشباع داده¬ها تا زمانی که هیچ داده جدیدی اضافه نشود به نمونه گیری ادامه می¬دهد. لذا در این مطالعه نیز نمونه گیری به صورت تدریجی تا اشباع داده¬ها ادامه می یابد.

**محدودیتهای اجرایی طرح و روش کاهش آنها**

خصوصیات فردی و اشتغالات فکری مشارکت کنندگان در پژوهش می تواند بر نحوه پاسخ آنان تاثیر بگذارد، که ازاختیار پژوهشگر خارج است.
در جریان مصاحبه با مشارکت کنندگان در پژوهش احتمال دارد افراد عقاید واقعی خود را بیان نکنند.و مطالبی را برای جلب نظر مصاحبه کننده اظهار نمایند که با توجه به خصوصیات نوع مصاحبه این محدودیت نیز ، حداقل تاثیر رابر ارایه مطالب خواهد داشت.
میزان اعتماد پاسخ دهندگان به مصاحبه یکسان نیست که از اختیار پژوهشگر خارج است.

Table of Variables

| **Variable Title** | **Variable Role** | **Variable Type** | **Scientific/Practical definition** | **How to measure** | **Scale** |
| --- | --- | --- | --- | --- | --- |
| سن زن | Independent | Quantitative/Continuous |  | پرسشنامه جمعیت شناختی | سال |
| سن مرد | Independent | Quantitative/Continuous |  | پرسشنامه جمعیت شناختی | سال |
| تحصیلات زن | Independent | Qualitative/Ordinal |  | پرسشنامه جمعیت شناختی | ابتدایی راهنمایی دبیرستان، دیپلم، دانشگاهی |
| تحصیلات مرد | Independent | Qualitative/Ordinal |  | پرسشنامه جمعیت شناختی | ابتدایی راهنمایی دبیرستان، دیپلم، دانشگاهی |
| شغل زن | Independent | Qualitative/Nominal |  | پرسشنامه جمعیت شناختی | خانه دار، شاغل |
| شغل مرد | Independent | Qualitative/Nominal |  | پرسشنامه جمعیت شناختی | بیکار، دانشجو، کارمند، کارگر، آزاد، بازنشسته، سایر |
| وضعیت اقتصادی | Independent | Qualitative/Ordinal |  | پرسشنامه جمعیت شناختی | ضعیف، متوسط، خوب، عالی |
| تعداد بارداری | Independent | Quantitative/Discrete |  | پرسشنامه جمعیت شناختی | عدد |
| تعداد زایمان | Independent | Quantitative/Discrete |  | پرسشنامه جمعیت شناختی | عدد |
| تعداد سقط | Independent | Quantitative/Discrete |  | پرسشنامه جمعیت شناختی | عدد |
| تعداد فرزند | Independent | Quantitative/Discrete |  | پرسشنامه جمعیت شناختی | عدد |
| نوع زایمان | Independent | Qualitative/Nominal |  | پرسشنامه جمعیت شناختی | واژینال/سزارین |
| جنسیت نوزاد فعلی | Independent | Qualitative/Nominal |  | پرسشنامه جمعیت شناختی | دختر/پسر |
| خواسته یا ناخواسته بودن بارداری | Independent | Qualitative/Nominal |  | پرسشنامه جمعیت شناختی | خواسته/ناخواسته |
| مدت زمان سپری شده از زایمان | Independent | Quantitative/Continuous |  | پرسشنامه جمعیت شناختی | عدد(برحسب ماه) |
| وضعیت حمایتی فرد | Independent | Qualitative/Ordinal |  | پرسشنامه جمعیت شناختی | ضعیف/متوسط/خوب |

Timetable

| Row | Activities | Month |  |
| --- | --- | --- | --- |
| 1 | مراحل دفاع از پروپوزال و انجام اصلاحات و تصویب نهایی آن | 1 month | \| 25 \| 24 \| 23 \| 22 \| 21 \| 20 \| 19 \| 18 \| 17 \| 16 \| 15 \| 14 \| 13 \| 12 \| 11 \| 10 \| 9 \| 8 \| 7 \| 6 \| 5 \| 4 \| 3 \| 2 \| 1 \| \| --- \| --- \| --- \| --- \| --- \| --- \| --- \| --- \| --- \| --- \| --- \| --- \| --- \| --- \| --- \| --- \| --- \| --- \| --- \| --- \| --- \| --- \| --- \| --- \| --- \| |
| 2 | جمع آوری داده های مرحله اول(مصاحبه، یادداشتهای عرصه)، پیاده نمودن مصاحبه ها، کدگذاری و تجزیه و تحلیل آنها | 3 month | \| 25 \| 24 \| 23 \| 22 \| 21 \| 20 \| 19 \| 18 \| 17 \| 16 \| 15 \| 14 \| 13 \| 12 \| 11 \| 10 \| 9 \| 8 \| 7 \| 6 \| 5 \| 4 \| 3 \| 2 \| 1 \| \| --- \| --- \| --- \| --- \| --- \| --- \| --- \| --- \| --- \| --- \| --- \| --- \| --- \| --- \| --- \| --- \| --- \| --- \| --- \| --- \| --- \| --- \| --- \| --- \| --- \| |
| 3 | طراحی مداخله (مرحله دوم مطالعه) | 1 month | \| 25 \| 24 \| 23 \| 22 \| 21 \| 20 \| 19 \| 18 \| 17 \| 16 \| 15 \| 14 \| 13 \| 12 \| 11 \| 10 \| 9 \| 8 \| 7 \| 6 \| 5 \| 4 \| 3 \| 2 \| 1 \| \| --- \| --- \| --- \| --- \| --- \| --- \| --- \| --- \| --- \| --- \| --- \| --- \| --- \| --- \| --- \| --- \| --- \| --- \| --- \| --- \| --- \| --- \| --- \| --- \| --- \| |
| 4 | اجرای مداخله | 1 month | \| 25 \| 24 \| 23 \| 22 \| 21 \| 20 \| 19 \| 18 \| 17 \| 16 \| 15 \| 14 \| 13 \| 12 \| 11 \| 10 \| 9 \| 8 \| 7 \| 6 \| 5 \| 4 \| 3 \| 2 \| 1 \| \| --- \| --- \| --- \| --- \| --- \| --- \| --- \| --- \| --- \| --- \| --- \| --- \| --- \| --- \| --- \| --- \| --- \| --- \| --- \| --- \| --- \| --- \| --- \| --- \| --- \| |
| 5 | جمع آوری داده های کمی و تجزیه و تحلیل آماری آنها | 5 month | \| 25 \| 24 \| 23 \| 22 \| 21 \| 20 \| 19 \| 18 \| 17 \| 16 \| 15 \| 14 \| 13 \| 12 \| 11 \| 10 \| 9 \| 8 \| 7 \| 6 \| 5 \| 4 \| 3 \| 2 \| 1 \| \| --- \| --- \| --- \| --- \| --- \| --- \| --- \| --- \| --- \| --- \| --- \| --- \| --- \| --- \| --- \| --- \| --- \| --- \| --- \| --- \| --- \| --- \| --- \| --- \| --- \| |
| 6 | نوشتن مقاله و اخذ پذیرش | 5 month | \| 25 \| 24 \| 23 \| 22 \| 21 \| 20 \| 19 \| 18 \| 17 \| 16 \| 15 \| 14 \| 13 \| 12 \| 11 \| 10 \| 9 \| 8 \| 7 \| 6 \| 5 \| 4 \| 3 \| 2 \| 1 \| \| --- \| --- \| --- \| --- \| --- \| --- \| --- \| --- \| --- \| --- \| --- \| --- \| --- \| --- \| --- \| --- \| --- \| --- \| --- \| --- \| --- \| --- \| --- \| --- \| --- \| |
| 7 | ارایه و گزارش نتایج تحقیق | 2 month | \| 25 \| 24 \| 23 \| 22 \| 21 \| 20 \| 19 \| 18 \| 17 \| 16 \| 15 \| 14 \| 13 \| 12 \| 11 \| 10 \| 9 \| 8 \| 7 \| 6 \| 5 \| 4 \| 3 \| 2 \| 1 \| \| --- \| --- \| --- \| --- \| --- \| --- \| --- \| --- \| --- \| --- \| --- \| --- \| --- \| --- \| --- \| --- \| --- \| --- \| --- \| --- \| --- \| --- \| --- \| --- \| --- \| |

24 Month

Personnel costs

No data was recorded for Personnel costs.

The cost of equipment and materials

No data was recorded for The cost of equipment and materials.

The cost of tests and and specialized services (within the university)

No data was recorded for The cost of tests and and specialized services (within the university).

The cost of tests and and specialized services (outside the university)

No data was recorded for The cost of tests and and specialized services (outside the university).

Travel Cost

No data was recorded for Travel Cost.

Other costs

No data was recorded for Other costs.

How to finance the research project

No data was recorded for How to finance the research project.

Ethical considerations

**ملاحظات و مشکلات اخلاقی طرح**

عدم اعتماد شرکت کنندگان در مطالعه به پژوهشگر
نگرانی در مورد افشای اسرار و نظرات آنان
نگرانی در مورد الزام به تداوم همکاری تا پایان طرح حتی در صورتی که در طول انجام پروژه تمایل خود برای همکاری را از دست بدهند
نگرانی در مورد علت انجام مصاحبه و اینکه نتایج در کجا ارایه خواهد شد

**راه حل مشکلات اخلاقی**

(1) کسب مجوز از معاونت پژوهشی دانشکده پرستاری و مامایی تهران
(2) کسب مجوز از معاونت پژوهشی دانشگاه علوم پزشکی کرمانشاه
(3)کسب مجوز از روسای بیمارستانهای وابسته به دانشگاه علوم پزشکی کرمانشاه
(4) کسب رضایتنامه ی آگاهانه از بیماران مشارکت کننده
(5) معرفی پژوهشگر به شرکت کنندگان
(6) روشن ساختن اهداف و چگونگی انجام پژوهش برای هر یک از شرکت کنندگان
(7)به کلیه شرکت کنندگان در مطالعه اطمینان داده میشود که کلیه اطلاعات ارایه شده از سوی آنان بصورت محرمانه ونزد محقق حفظ خواهد شد.
(8) ذکر بدون نام و نشان بودن مصاحبه ها
(9) به شرکت کنندگان یادآوری می گردد که شرکت در مطالعه کاملاً اختیاری بوده و آنها مجاز هستند تا در هر قسمتی ازمطالعه در مورد ادامه و یا قطع همکاری با پژوهشگر تصمیم گیری نمایند.
(10) احترام به تمامیت
(11)اخذ مصوبه اخلاق برای انجام پژوهش

KTE

**1) آیا پژوهش به سفارش کارفرما انجام می شود و بخشی و یا تمام بودجه آن توسط سفارش دهنده تامین می گردد؟**

No

**لطفا نام سازمان را وارد نموده و مستند آن را در قسمت ضمائم طرح آپلود کنید.**

**2) آیا پژوهش به سفارش کارفرما انجام می شود و تمام بودجه آن توسط دانشگاه تامین می گردد؟**

Yes

**لطفا نام سازمان را وارد نموده و مستند آن را در قسمت ضمائم طرح آپلود کنید.**

دانشگاه علوم پزشکی تهران، دانشکده پرستاری و مامایی

**3) آیا ذی نفعان مستقیم نتایج پژوهش، مشارکت فعال در انجام پژوهش دارند؟**

Yes

**4) آیا پژوهش بر اساس اولویت های اعلام شده یک سازمان می باشد؟**

Yes

**لطفا نام سازمان را وارد نموده و فایل اولویت ها را در قسمت ضمائم طرح آپلود کنید.**

دانشکده پرستاری و مامایی دانشگاه علوم پزشکی تهران

**5) آیا هدف آن حل کردن یک چالش سلامت می باشد؟**

Yes

**توضیح داده شود چگونه نتایج پژوهش می تواند چالش را برطرف نماید.**

اگر برنامه های جامع مراقبت حمایتی در دوره پس از زایمان با پوشش دهی همه جانبه برای زنان اتخاذ گردد می توان در خصوص رفع نیازهای زنان در دوره بحرانی پس از زایمان اطمینان اخذ کرد و این نیاز مند انجام ارزیابی، بررسی دغدغه ها و ابعاد مختلف مشکلات زنان در دوره پس از زایمان از دیدگاه خود آنان و بررسی تاثیر چنین برنامه ای بر سلامتی زنان و نوزادان، همچنین اضطراب و اعتماد به نفس والدین؛ و تاثیرات اقتصادی برخدمات سلامت زنان و خانواده هایشان باشد. از آن جا که برنامه ریزی دقیق و ارزیابی جدید برای اطمینان از کفایت مراقبت های پس از زایمان مورد نیاز است. باید رویکردهایی اتخاذ شود که مشکلاتی که پدران و مادران را در دستیابی به مراقبت های جامع پس از زایمان احاطه کرده است به طور کامل و با رویکرد مراقبت حمایتی جامع پوشش داده شود و نباید صرفا به مراقبت های محدود ارایه شده به زنان پس از ترخیص از بیمارستان بسنده نمود(16). این امر مستلزم انجام پژوهش های کیفی در راستای تبیین نیاز ها و مشکلات زوجین در این برهه زمانی است. از طرفی با توجه به اینکه تاکنون در ایران پژوهشی کیفی حول محور این موضوع انجام نشده و مطالعات قبلی صرفا به صورت کمی، کیفیت مراقبت های پس از زایمان را مورد سنجش قرار داده اند، لذا درراستای اولویت پژوهشی گروه مامایی و سلامت باروری دانشگاه علوم پزشکی تهران مبنی بر طراحی، اجرا،پایش و ارزشیابی مداخلات مرتبط با کاهش موربیدیته مادران و نوزادان پس از زایمان این مطالعه قصد دارد با یک رویکرد ترکیبی به تبیین نیازهای مراقبت حمایتی زوجین در دوره پس از زایمان پرداخته و یک مداخله مبتنی بر نیاز در راستای ارتقا خدمات سلامت در دوران پس از زایمان را طراحی و اجرا نماید. در صورت اثربخشی مداخله طراحی شده در اختیار سیاست گذاران و مدیران بهداشتی قرار داده خواهد شد تا در جهت ارتقا کیفیت مراقبت های پس از زایمان از آن استفاده نمایند.

**6) آیا نتیجه پژوهش قابلیت ایجاد تغییر و اثر را دارد؟**

Yes

**نوع اثر و گروه مخاطب تغییر به صورت توضیح نوشته شود.**

گروه مخاطب: زنان و همسران آن ها در دوره پس از زایمان
نوع اثر: کمک به ارتقا کفایت مراقبت های پس از زایمان و پیشنهاد برنامه جامع مراقبت حمایتی با پوشش دهی همه جانبه

**7) آیا نتیجه این پژوهش برای اینکه بتواند منجر به ایجاد تغییر گردد، باید در کنار نتایج سایر پژوهش ها قرار بگیرد تا بتوانند پیشنهاد مشخص بدهند؟**

Yes

**سایر پژوهش های مورد نیاز مشخص شود**

مطالعات مروری در حوزه نیاز های مراقبت حمایتی مادران و پدران در اقوام و ملیت های مختلف
انجام کارآزمایی های بالینی در خصوص ارزیابی اثربخشی مداخله پیشنهادی طرح حاضر

**8) آیا نتیجه پژوهش می تواند منجر به تولید محصول اولیه (پروتوتیپ) و یا ثبت پتنت گردد؟**

No

**9) آیا در مورد مطالعه حاضر پاسخ شما به حداقل یکی از سوالات بالا مثبت می باشد؟**

Yes

Self-deceleration of research products

No data was recorded for Self-deceleration of research products .

attachments

| **Name** | **Type** | **Subject** | **Date** | **Download** |
| --- | --- | --- | --- | --- |
| Informed consent.doc |  | فرم رضایت آگاهانه | 2024/02/01 19:19:14 | [Download](file://\\j-fs04\J-PLOS-L\Production\PONE\pone.0350038\FROM_CLIENT\Accepted_manuscripts\pone_637862bf-78aa-482b-a38f-14f0a97e4dc3\download?xyz=s-9TEuZMjPUEj1I2WVwnU3i5uHRfxtp7Ti-ZUylK8f73J0N2KycZ2OJyQ3Ed7DQVX2izp253ELEeTSwUCGLi-1QB5W6DPZbGbZgsRHWlLzxbna1q7B7iX3f41DXoSBiX&csrf_token=MTc1MjY4OTQ0Nzg0ZU1ZUGRxR2lKakZzNTN6Umkwc3RZOGluVjBTR0JC) |
| Research priorities of midwifery and reproductive health education group.pdf |  | Research priorities of midwifery and reproductive health education group | 2024/02/01 22:11:55 | [Download](file://\\j-fs04\J-PLOS-L\Production\PONE\pone.0350038\FROM_CLIENT\Accepted_manuscripts\pone_637862bf-78aa-482b-a38f-14f0a97e4dc3\download?xyz=K7NNoUDCOoGb33ZeaymWHDqsoqayonfu7x8eq8RB1TE3W2mydlAuCk28EEbhOYsHvAfy8VQpFOmv9wbTD1Gz9pE-nO66hn-ZGA3qMx4Ma63qwCfxNHY8KafdEukE1AvC&csrf_token=MTc1MjY4OTQ0Nzg0ZU1ZUGRxR2lKakZzNTN6Umkwc3RZOGluVjBTR0JC) |
| Proposal approval form.jpg |  | Proposal approval form | 2024/02/01 22:12:45 | [Download](file://\\j-fs04\J-PLOS-L\Production\PONE\pone.0350038\FROM_CLIENT\Accepted_manuscripts\pone_637862bf-78aa-482b-a38f-14f0a97e4dc3\download?xyz=IEsn-O0c5CEyXIggZ1rXA07wOFyzAS_6d3wZb97yzgHsycAhu-cKKcV36s-7jqmRFtDfNTANoRoP0VoYX_IGS5eklBPQ2jWK3MuHoyjf1T_UxPJQqHvcyERxb0eKeMa6&csrf_token=MTc1MjY4OTQ0Nzg0ZU1ZUGRxR2lKakZzNTN6Umkwc3RZOGluVjBTR0JC) |
| Minutes of the proposal defense meeting.pdf |  | Minutes of the proposal defense meeting | 2024/02/04 08:23:11 | [Download](file://\\j-fs04\J-PLOS-L\Production\PONE\pone.0350038\FROM_CLIENT\Accepted_manuscripts\pone_637862bf-78aa-482b-a38f-14f0a97e4dc3\download?xyz=JURItHo2nQku7WXkPN7mFUZm5MKDh1H244cKGJl6H7k4P0XYTKXVGHAjNbdinHkpTusvvGS7DWPwTuYVQzsFmwNHMmWinmjOJ4kOU6r_SEzQVIfMMhS9CIPu4NMlXzDY&csrf_token=MTc1MjY4OTQ0Nzg0ZU1ZUGRxR2lKakZzNTN6Umkwc3RZOGluVjBTR0JC) |
| Descriptions related to the quantitative part of the study (questionnaire and sample size in the quantitative part).docx |  | Descriptions related to the quantitative part of the study (questionnaire and sample size in the quantitative part) | 2024/02/07 17:52:54 | [Download](file://\\j-fs04\J-PLOS-L\Production\PONE\pone.0350038\FROM_CLIENT\Accepted_manuscripts\pone_637862bf-78aa-482b-a38f-14f0a97e4dc3\download?xyz=8BXbMYzAQmIpCK-bHF9FlCQ-mHRIAnr_IvdKadtBrUnsHvrbM0CqgWKnoidUubWyta6OeKIy0DcdJOv53-RCGZ5HyQqWBrdmd6Jmr2WtbiAnpOMp8U7wPGyFSF4pMexm&csrf_token=MTc1MjY4OTQ0Nzg0ZU1ZUGRxR2lKakZzNTN6Umkwc3RZOGluVjBTR0JC) |
| Sample interview questions.docx |  | Sample interview questions | 2024/02/07 17:53:22 | [Download](file://\\j-fs04\J-PLOS-L\Production\PONE\pone.0350038\FROM_CLIENT\Accepted_manuscripts\pone_637862bf-78aa-482b-a38f-14f0a97e4dc3\download?xyz=T3Pk7JV1yIgxYW_gFOMePSn0CDV7qGwv3_x8HAcS_sC-ysRhhu5YINratf7bFIfL5Cn3uwzo4ZkVfUTx9PIqu7KwcT8ZYziR-FG719xiuIaddJE_tJ5dfRIuZlHshkjh&csrf_token=MTc1MjY4OTQ0Nzg0ZU1ZUGRxR2lKakZzNTN6Umkwc3RZOGluVjBTR0JC) |
| proposal.docx |  | پروپوزال اصلاح شده | 2024/02/28 11:25:58 | [Download](file://\\j-fs04\J-PLOS-L\Production\PONE\pone.0350038\FROM_CLIENT\Accepted_manuscripts\pone_637862bf-78aa-482b-a38f-14f0a97e4dc3\download?xyz=1yxNX6jaPo3NOu5htp5J04s-FJ0ezwclqAbHBdQ3wp1soc5-csNa5JGktpo1GpeBMgfH1W6pLBsD2WtT6lUElhuS5lA2vcAQJbCXz3cFPVIouunJ-a_ghINRdgeOh6CK&csrf_token=MTc1MjY4OTQ0Nzg0ZU1ZUGRxR2lKakZzNTN6Umkwc3RZOGluVjBTR0JC) |
